# Association between multimorbidity and socioeconomic deprivation on short-term mortality among patients with diffuse large B-cell or follicular lymphoma in England: a nationwide cohort study

**DOI:** 10.1136/bmjopen-2021-049087

**Published:** 2021-11-30

**Authors:** Matthew James Smith, Edmund Njeru Njagi, Aurelien Belot, Clémence Leyrat, Audrey Bonaventure, Sara Benitez Majano, Bernard Rachet, Miguel Angel Luque Fernandez

**Affiliations:** 1 Non-Communicable Disease Epidemiology, London School of Hygiene and Tropical Medicine, London, UK; 2 Department of Medical Statistics, London School of Hygiene and Tropical Medicine, London, UK; 3 Epidemiology of Childhood and Adolescent Cancers Team, University of Paris, Paris, France; 4 Noncommunicable Disease and Cancer Epidemiology Group, Instituto de Investigación Biosanitaria de Granada, Granada, Spain

**Keywords:** epidemiology, statistics & research methods, lymphoma

## Abstract

**Objectives:**

We aimed to assess the association between multimorbidity and deprivation on short-term mortality among patients with diffuse large B-cell (DLBCL) and follicular lymphoma (FL) in England.

**Setting:**

The association of multimorbidity and socioeconomic deprivation on survival among patients diagnosed with DLBCL and FL in England between 2005 and 2013. We linked the English population-based cancer registry with electronic health records databases and estimated adjusted mortality rate ratios by multimorbidity and deprivation status. Using flexible hazard-based regression models, we computed DLBCL and FL standardised mortality risk by deprivation and multimorbidity at 1 year.

**Results:**

Overall, 41 422 patients aged 45–99 years were diagnosed with DLBCL or FL in England during 2005–2015. Most deprived patients with FL with multimorbidities had three times higher hazard of 1-year mortality (HR: 3.3, CI 2.48 to 4.28, p<0.001) than least deprived patients without comorbidity; among DLBCL, there was approximately twice the hazard (HR: 1.9, CI 1.70 to 2.07, p<0.001).

**Conclusions:**

Multimorbidity, deprivation and their combination are strong and independent predictors of an increased short-term mortality risk among patients with DLBCL and FL in England. Public health measures targeting the reduction of multimorbidity among most deprived patients with DLBCL and FL are needed to reduce the short-term mortality gap.

**Strengths and limitations of this study**

- Data contain a large sample size of high-quality population-based clinical records with a high national coverage of information on all patients diagnosed with diffuse large B-cell or follicular lymphomas in England during 2005–2013.
- Population-based administrative hospital discharge data were used for the assessment of comorbid conditions, and selection bias was reduced by restricting records of comorbidities to occurring between 6 and 24 months prior to the date of cancer diagnosis.
- Cumulative mortality hazard up to 1 year since cancer diagnosis was modelled using a flexible parametric modelling approach and included restricted cubic splines to account for non-linear effects of continuous variables.
- Regression standardisation was used to control for confounding of patient baseline characteristics; information on lifestyle characteristics was unavailable.
- As there were missing data, we performed a sensitivity analysis by comparing complete case analysis-based results to those after multiple imputation: the conclusions were consistent.

## Introduction

In England, non-Hodgkin’s lymphoma (NHL) is the sixth most commonly diagnosed cancer in England with an incidence rate of 23.2 per 100 000 people.^1^ Apart from lung cancer, survival estimates of NHL (79.4% survival probability at 1 year) are among the lowest of the six most common cancers.^2 3^ NHL encompasses a heterogeneous group of malignancies with diverse histological patterns; in addition, the most common NHL subtypes are diffuse large B-cell lymphoma (DLBCL) and follicular lymphoma (FL),^4^ patients of which show a large variation in survival.^5^ Cancer survival in England is lower than other European countries,^6^ but is similar when restricting to those surviving after 1 year:^7^ identifying, and influencing, the factors of short-term mortality could reduce the gap in survival.

Over the past decades, patient survival of FL has steadily improved and stagnated for DLBCL;^3^ furthermore, disparities in survival between deprivation levels remain.^8^ Public health policies have increased awareness and set targets, such as minimising the length of time from referral to treatment, to reduce the inequalities;^9^ however, since their implementation, there has been no evidence that the National Health Service (NHS) Cancer Plan had an impact on the inequalities in cancer survival.^10^ The deprivation gap in survival is still apparent, despite the NHS Cancer Plan and successive policies.^9^

Comorbidities, which refers to the presence of a long-term health condition additional, but unrelated, to the underlying cancer,^11^ tend to mask cancer symptoms, delaying cancer diagnosis and decrease survival.^12^ Older age groups and those living in more deprived areas experience more comorbidities. With a global ageing population, the prevalence of comorbidities is expected to increase.^13^ The association between multimorbidity and survival is described for other cancers,^14^ but for DLBCL and FL this relationship remains unclear.

We aim to identify the association between multimorbidity and risk of short-term mortality among patients with DLBCL and FL in England. We hypothesise that multimorbidity and deprivation, independently and combined, contribute to an increased risk of death after DLBCL or FL diagnosis.

## Methods

### Study design, participants, data and setting

We used the data from a retrospective population-based cohort study with patients with DLBCL and FL diagnosed between 1 January 2005 and 31 December 2013 and followed up to 31 December 2015. DLBCL and FL diagnoses were made according to the International Classification of Diseases for Oncology, third edition, based on codes C82.0-C85.9^15^ (online supplemental table S1 shows the subtype categorisation). Patients entered the study on the date of their diagnosis and were followed up until death or censored at 1 year, whichever occurred first.

10.1136/bmjopen-2021-049087.supp1 Supplementary data

Data were obtained from population-based cancer registries within the English National Cancer Registry and Analysis Service (CAS)^16^ and linked to patient’s electronic health records from Hospital Episode Statistics (HES). CAS contains patient and tumour variables including relevant dates (birth, diagnosis and vital status), sex, age at diagnosis, deprivation, cancer site and morphology. We used population-based administrative hospital discharge data for the assessment of comorbid conditions; we analysed HES data (containing comorbid conditions records) according to the International Classification of Diseases, 10th revision (online supplemental table S2), for the period 2003–2015. HES contains clinical, administrative and demographic information about individual patients. To avoid selection bias including cancer-related comorbidities, we restricted retrospective records of comorbidities to occurring between 6 and 24 months prior to the date of DLBCL and FL diagnosis.^17^

### Patient and public involvement

No patient or public involvement.

### Outcome, exposure and other variables

**Figure 1:**
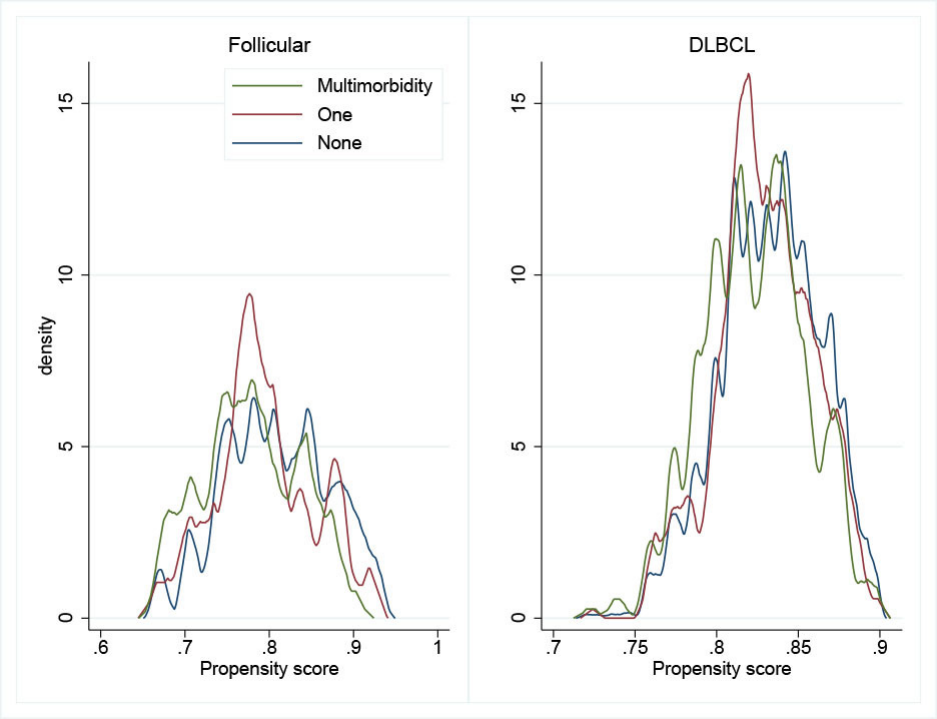
Overlap plots for the density of predicted probabilities of comorbidity status among patients (n=41 422), aged 45–99, in England diagnosed with non-Hodgkin’s lymphoma during 2005–2013. Propensity score: relates to the predicted probability of having any comorbidity level as measured by a multinomial logistic regression model conditioning on the independent variables (ie, age at diagnosis, sex, deprivation level and ethnicity). DLBCL, diffuse large B-cell lymphoma.

The outcome of this study was the time since diagnosis up to death observed within the first year after diagnosis of patients with DLBLC and FL (patients alive were censored at survival time defined with the date of last known vital status), the main exposures were multimorbidity status and deprivation. Due to data availability and clinical reasoning, we include as confounders age, sex and ethnicity. Due to the positivity assumption, and the chances of having a multimorbidity, we included patients aged above 45 years at diagnosis (figure 1). The positivity assumption states that there is a non-zero probability of receiving any level of comorbidity status for every combination of values of the independent variables among the patients in the population.^18^

Comorbidity status was classified according to the Royal College of Surgeons (RCS) Charlson score (an adaptation of the Charlson comorbidity index^19^) that includes 12 categories for comorbidities, excludes a category (peptic ulcer disease) and groups diseases together (eg, diabetes mellitus codes with or without complications were grouped into a single category). The score was categorised into those with none, one comorbidity (whatever the type) or two or more comorbidities (defined as *multimorbidity*). The score does not weight the comorbidities assuming that any comorbidity has the same impact on short-term mortality.^20^

Area-level deprivation was categorised into one of five quintiles (fifth is most deprived). Deprivation was used as a proxy of individual level socioeconomic status. We used the Index of Multiple Deprivation^21^ (IMD), which is an area-level deprivation score based on the Lower Super Output Area^22^ (LSOA) residence of the patient at the time of cancer diagnosis. LSOA is a geographical location with a median of 1500 inhabitants.

Ethnicity due to data sparsity among ethnic minorities was recorded as either white or other. Route to diagnosis (CAS dataset), although not considered in our analysis because it was on the causal pathway, is included in the imputation models for missing data.

### Statistical analysis

We described the characteristics of patients with DLBLC and FL using counts and proportions, and calculated ORs of having a lymphoma type along with Wald test p values (online supplemental table S3). We assessed the unadjusted association between having multimorbidity and patients’ characteristics, using χ^2^ tests. Then, we compute the number of deaths, person-time at risk and unadjusted rates of deaths per 100 person-years and rate ratios with 95% CIs by patients’ characteristics by DLBCL and FL subtypes.

The follow-up time for those who died is from the date of diagnosis until death, for those alive it was until administrative censoring at 12 months (no lost to follow-up before 12 months was observed). To describe the multimorbidity and deprivation short-term mortality risk among patients with DLBCL and FL, we computed the 1 year cumulative hazard obtained using the non-parametric Nelson-Aalen estimator.^23^ Then, we computed adjusted short-term mortality risk by patient characteristics using a flexible parametric modelling approach to model the non-linear change in mortality risk over 1 year. We included restricted-cubic spline to model the baseline hazard,^24^ with three knots located at the 25th, 50th and 75th percentiles of the log event times. To define the model, let t be the time since DLBCL or FL diagnosis until death or censoring. We define the log cumulative motality hazard as

$$\ln\left[H\left(t|x_{i},A_{i},Dep_{i}\right)\right]=\gamma_{0}+\gamma_{1}z_{1i}+\gamma_{2}z_{2i}+\gamma_{3}A_{1i}+\;\gamma_{4}A_{2i}+\sum_{k=2}^{5}\beta_{0k}\cdot Dep_{ik}+\sum_{m=1}^{M}\beta_{m}x_{im}$$

where $z_{i}$ are the indicators for the three knots of the baseline restricted cubic splines. The model specification included the restricted cubic splines for the continuous variable age are given by $A_{i}$ where the knots are placed at 1 and 6 months, deprivation, and the vector $x_{im}$ of categorical covariates (ie, multimorbidity, ethnicity and sex). Restricted cubic splines were included to minimise residual confounding and to account for the non-linear association between age and the cumulative hazard. From the model, we derived the cumulative incidence of death at 1 year by comorbidity status (ie, none, one or multimorbidity) standardised to the empirical distribution of age, sex, ethnicity and deprivation.^25 26^

We used the same modelling approach to evaluate the linearly combined effect of multimorbidity and derivation on short-term mortality, assuming the effect of multimorbidity is constant across levels of deprivation.^27^ We assume that both effects (multimorbidity and deprivation) are independently associated with the mortality rate and that their effect is constant and the rate is increasing linearly.

In sensitivity analysis, we evaluated the robustness of our results to the missing ethnicity records by utilising multiple imputation using chained equations (the fully conditional specification multiple imputation approach), under a missing at random assumption. We included route of cancer diagnosis and Ann Arbor^28^ cancer stage as partially observed auxiliary variables because these variables were predictive of the probability of missing values (established via exploratory analysis of the missing data indicators) and are predictive of the underlying values themselves (established from clinical and epidemiological reasoning).^29^ We generated 20 imputed datasets. The imputation model for the partially observed variable (ethnicity) was defined as a logistic regression model including all explanatory variables in the substantive model, the vital status and Nelson-Aalen estimate of the cumulative hazard, and the auxiliary variables (route to, and stage at, diagnosis). We then fit the substantive model to each of the 20 imputed datasets and from these estimates we predicted the standardised survival (using *stpm2* package), derived the cumulative hazard (−log(Survival probability)) for each imputed dataset and combined the survival probability estimates using Rubin’s rules.^30^

Analyses were performed in Stata V.16 (StataCorp); the *stpm2* package was used to estimate flexible parametric survival models, and the *standsurv* command to compute standardised mortality risks. The *mi impute* command was used for multiple imputation and the *mi estimate* command to combine estimates.

## Results

Overall, 41 422 patients in England, aged from 45 to 99 years, were diagnosed with either DLBCL or FL between 2005 and 2013 in England. Of 14 043 patients with FL, among those who died within 1 year, the proportion of patients with multimorbidity compared with no comorbidity was 3.2 times higher (22.0% vs 6.9%, respectively) (table 1); of 27 379 patients diagnosed with DLBCL, this comparison was 1.6 times higher (49.7% vs 31.5%, respectively). For both DLBCL and FL subtypes, the proportion of multimorbidity was higher among those living in more deprived areas. In DLBCL only, there was a higher proportion of having any comorbidity among men compared with women (p<0.001); for FL only, this proportion was slightly higher among women. As expected, multimorbidity was more prevalent among older age groups.

**Table 1:** Vital status, age, sex, deprivation level and ethnicity according to the comorbidity status among n=41 422 patients with non-Hodgkin’s lymphoma in England between 2005 and 2013 (27 379 diffuse large B-cell lymphoma (DLBCL) cases and 14 043 follicular lymphoma (FL) cases)

	No comorbidity N (%)	Comorbidity N (%)	Multimorbidity N (%)	P value*
**Diffuse large B-cell lymphoma (n=27 379)**				
Vital status at 1 year				
Alive	16 621 (68.5)	872 (56.7)	791 (50.3)	<0.001
Dead	7648 (31.5)	666 (43.3)	781 (49.7)	
Sex				
Male	12 904 (53.2)	794 (51.6)	954 (60.7)	<0.001
Female	11 365 (46.8)	744 (48.4)	618 (39.3)	
Age at diagnosis (years)				
45–54	2685 (11.1)	80 (5.2)	96 (6.1)	<0.001
55–64	5154 (21.2)	257 (16.7)	200 (12.7)	
65–74	7337 (30.2)	432 (28.1)	439 (27.9)	
75+	9093 (37.5)	769 (50.0)	837 (53.2)	
Deprivation				
Least deprived	5348 (22.0)	291 (18.9)	256 (16.3)	<0.001
2	5586 (23.0)	318 (20.7)	334 (21.3)	
3	5115 (21.1)	324 (21.1)	317 (20.2)	
4	4665 (19.2)	337 (21.9)	339 (21.6)	
Most deprived	3555 (14.7)	268 (17.4)	326 (20.7)	
Ethnicity				
White	17 831 (95.5)	1204 (96.6)	1169 (92.6)	<0.001
Other	848 (4.5)	43 (3.5)	93 (7.4)	
**Follicular lymphoma (n=14 043)**				
Vital status at 1 year				
Alive	12 003 (93.1)	546 (87.6)	407 (78.0)	<0.001
Dead	895 (6.9)	77 (12.4)	115 (22.0)	
Sex				
Male	5980 (46.4)	275 (44.1)	257 (49.2)	0.227
Female	6918 (53.6)	348 (55.9)	265 (50.8)	
Age at diagnosis (years)				
45–54	2246 (17.4)	42 (6.7)	33 (6.3)	<0.001
55–64	3769 (29.2)	140 (22.5)	82 (15.7)	
65–74	3952 (30.6)	199 (31.9)	159 (30.5)	
75+	2931 (22.7)	242 (38.8)	248 (47.5)	
Deprivation				
Least deprived	3091 (24.0)	113 (18.1)	80 (15.3)	<0.001
2	3025 (23.5)	122 (19.6)	81 (15.5)	
3	2759 (21.4)	136 (21.8)	118 (22.6)	
4	2356 (18.3)	123 (19.7)	130 (24.9)	
Most deprived	1667 (12.9)	129 (20.7)	113 (21.7)	
Ethnicity				
White	9226 (95.7)	498 (96.3)	386 (92.8)	0.012
Other	412 (4.3)	19 (3.7)	30 (7.2)	

Missing values: ethnicity n(%): DLBCL=6191 (22.6%), FL=3472 (24.7%).

*Χ^2^ test of association between the baseline characteristic and comorbidity status.

Tables 2 and 3 show the person-time at risk and unadjusted mortality rate of death for DLBLC and FL at 1 year after diagnosis. Among patients with FL and DLBCL, 1087 (7.7%) and 9095 (33.2%) died before 1 year, respectively (tables 2 and 3). Among FL, those with one comorbidity or multimorbidity had 1.9 (95% CI 1.46 to 2.33, p<0.001) or 3.5 (95% CI 2.90 to 4.27, p<0.001) times the mortality rate, respectively, compared with those with no comorbidity (table 2). Among DLBCL, those with one comorbidity or multimorbidity had 1.5 (95% CI 1.41 to 1.66, p<0.001) or 1.9 (95% CI 1.78 to 2.06, p<0.001) times the mortality rate, compared with no comorbidity (table 3).

**Table 2:** One-year unadjusted mortality rates and rate ratios by sex, age, deprivation, ethnicity and comorbidity status among patients with follicular lymphoma in England between 2005 and 2015 (n=14 043; 1087 deaths at 1 year)

	Deaths/person years	Mortality rate* (95% CI)	Mortality RR	95% CI	P value
**One-year mortality (n=1087)**					
Sex					
Male	526/6218.12	8.5 (7.77 to 9.21)	Ref		
Female	561/7225.93	7.8 (7.15 to 8.43)	0.92	(0.82 to 1.03)	0.157
Age at diagnosis (years)†					
10-year increase	–	–	2.20	(2.08 to 2.34)	<0.001
Age at diagnosis (years)					
45–54	46/2300.66	2.0 (1.50 to 2.67)	Ref		
55–64	129/3926.24	3.3 (2.77 to 3.90)	1.64	(1.17 to 2.30)	0.004
65–74	274/4157.52	6.6 (5.86 to 7.42)	3.30	(2.41 to 4.50)	<0.001
75+	638/3059.62	20.9 (19.30 to 22.54)	10.43	(7.73 to 14.07)	<0.001
Deprivation					
Least deprived	211/3175.55	6.7 (5.81 to 7.60)	Ref		
2	227/3102.18	7.3 (6.43 to 8.33)	1.10	(0.91 to 1.33)	0.313
3	230/2884.20	8.0 (7.01 to 9.08)	1.20	(1.00 to 1.45)	0.055
4	240/2471.30	9.7 (8.56 to 11.02)	1.46	(1.23 to 1.76)	<0.001
Most deprived	179/1810.81	9.9 (8.54 to 11.45)	1.49	(1.22 to 1.82)	<0.001
Ethnicity					
White	742/9712.99	7.6 (7.11 to 8.21)	Ref		
Other	21/450.96	4.7 (3.04 to 7.14)	0.61	(0.40 to 0.94)	0.024
Comorbidity status					
None	895/12 412.44	7.5 (7.03 to 7.97)	Ref		
One	77/577.89	13.3 (10.66 to 16.66)	1.85	(1.46 to 2.33)	<0.001
Multimorbidity	115/453.71	25.4 (21.11 to 30.43)	3.52	(2.90 to 4.27)	<0.001

Missing values: ethnicity n (%) = 3472 (24.7%).

*Per 100 person years.

†Continuous form of age (for each 10-year increase in age).

RR, rate ratio.

**Table 3:** One-year unadjusted mortality rates by sex, age, deprivation, ethnicity and comorbidity status among patients with diffuse large B-cell lymphoma in England between 2005 and 2015 (n=27 379; 9095 deaths at 1 year)

	Deaths/person years	Mortality rate* (95% CI)	Mortality RR	95% CI	P value
**One-year mortality (n=9095)**					
Sex					
Male	4867/11 318.43	43.0 (41.81 to 44.23)	Ref	Ref	Ref
Female	4228/9784.59	43.2 (41.93 to 44.53)	1.01	(0.96 to 1.05)	0.817
Age at diagnosis (years)†					
10-year increase	–	–	1.50	(1.47 to 1.53)	<0.001
Age at diagnosis (years)					
45–54	430/2615.81	16.4 (14.96 to 18.07)	Ref	Ref	Ref
55–64	1074/4937.54	21.8 (20.49 to 23.09)	1.32	(1.18 to 1.48)	<0.001
65–74	2365/6604.47	35.8 (34.39 to 37.28)	2.18	(1.97 to 2.41)	<0.001
75+	5226/6945.19	75.2 (73.23 to 77.31)	4.58	(4.15 to 5.05)	<0.001
Deprivation					
Least deprived	1765/4684.43	37.7 (35.96 to 38.48)	Ref		
2	1955/4898.09	39.9 (38.18 to 41.72)	1.06	(0.99 to 1.13)	0.079
3	1948/4424.61	44.0 (42.11 to 46.03)	1.17	(1.10 to 1.25)	<0.001
4	1908/4017.48	47.5 (45.41 to 49.67)	1.26	(1.18 to 1.35)	<0.001
Most deprived	1519/3078.40	49.3 (46.92 to 51.89)	1.31	(1.22 to 1.40)	<0.001
Ethnicity					
White	6351/15 900.73	39.9 (38.97 to 40.94)	Ref		
Other	270/808.61	33.4 (29.64 to 37.62)	0.84	(0.74 to 0.94)	0.004
Comorbidity status					
None	7648/19 007.45	40.2 (39.35 to 41.15)	Ref		
One	666/1081.20	61.6 (57.09 to 66.46)	1.53	(1.41 to 1.66)	<0.001
Multimorbidity	781/1014.36	77.0 (71.78 to 82.59)	1.91	(1.78 to 2.06)	<0.001

Missing values: ethnicity n(%) = 6,191 (22.6%).

*Per 100 person-years.

†Continuous form of age (for each 10-year increase in age).

RR, rate ratio.

The unadjusted mortality rate of death increased with each increase in deprivation level: those living in the most deprived areas had 1.5 (95% CI 1.22 to 1.82, p<0.001) and 1.3 (95% CI 1.22 to 1.40, p<0.001) times the mortality rate compared with those living in the least deprived areas, for FL and DLBCL, respectively.

Table 4 shows, before and after multiple imputation, the mortality hazard among patients with DLBCL and FL at 1 year adjusted for comorbidity status, sex, age, deprivation and ethnicity. After multiple imputation, patients with multimorbidity had 2.2 (CI 1.78 to 2.64, p<0.001) and 1.4 (CI 1.34 to 1.55, p<0.001) times the mortality hazard compared with those without a comorbidity, for FL and DLBCL, respectively. Patients in more deprived areas had 1.5 (CI 1.23 to 1.84, p<0.001) and 1.3 (CI 1.21 to 1.40, p<0.001) times the mortality hazard compared with those living in the least deprived areas, for FL and DLBCL, respectively. There was evidence of a linear trend in mortality hazard by deprivation level for FL (p<0.001) and DLBCL (p<0.001). The direction and magnitude of the HRs after multiple imputation were similar to complete case analysis.

**Table 4:** Adjusted HRs of death (before and after multiple imputation) for all patient characteristics among patients with (A) follicular or (B) diffuse large B-cell lymphoma in England between 2005 and 2015

	Complete case			After multiple imputation		
	HR*	95% CI	P value	HR*	95% CI	P value
**(A) Follicular**						
Sex						
Male	Ref	Ref	–	Ref	Ref	–
Female	1.08	0.85 to 1.38	0.540	0.84	0.74 to 0.94	<0.001
Comorbidity status						
None	Ref	Ref	–	Ref	Ref	–
One	1.52	1.15 to 2.03	<0.001	1.28	1.01 to 1.61	<0.041
Multimorbidity	2.36	1.85 to 3.02	<0.001	2.17	1.78 to 2.64	<0.001
Deprivation†						
Least	Ref	Ref	–	Ref	Ref	–
2	1.03	0.70 to 1.53	0.873	1.09	0.90 to 1.31	0.378
3	1.47	1.00 to 2.16	0.051	1.15	0.95 to 1.39	0.143
4	1.30	0.87 to 1.94	0.200	1.44	1.19 to 1.73	<0.001
Most	1.63	1.11 to 2.41	0.013	1.50	1.23 to 1.84	<0.001
Ethnicity						
White	Ref	Ref	–	Ref	Ref	–
Other	0.49	0.27 to 0.88	0.017	0.64	0.40 to 1.01	0.053
**(B) Diffuse large B-cell**						
Sex						
Male	Ref	Ref	–	Ref	Ref	–
Female	0.91	0.84 to 0.99	0.170	0.90	0.86 to 0.93	<0.001
Comorbidity status						
None	Ref	Ref	–	Ref	Ref	–
One	1.29	1.18 to 1.42		1.24	1.15 to 1.35	<0.001
Multimorbidity	1.61	1.47 to 1.76	<0.001	1.44	1.34 to 1.55	<0.001
Deprivation†						
Least	Ref	Ref	–	Ref	Ref	–
2	1.17	1.03 to 1.33	0.013	1.05	0.98 to 1.12	0.155
3	1.15	1.01 to 1.30	0.029	1.13	1.06 to 1.20	<0.001
4	1.25	1.11 to 1.42	<0.001	1.23	1.15 to 1.31	<0.001
Most	1.32	1.16 to 1.51	<0.001	1.30	1.21 to 1.40	<0.001
Ethnicity						
White	Ref	Ref	–	Ref	Ref	–
Other	0.93	0.77 to 1.13	0.463	1.03	0.91 to 1.16	0.678

Missing values: (A) ethnicity n(%)=3,472 (24.7%), (B) ethnicity n(%)=6,191 (22.6%).

*Adjusted for sex, comorbidity status, deprivation, ethnicity and the restricted cubic splines of age.

†Likelihood ratio test for the overall effect of deprivation (p<0.001).

Table 5 shows the linearly combined effect^14^ between comorbidity status and deprivation on short-term mortality by DLBCL and FL. Overall, at 1 year since diagnosis, among FL (table 5), patients who were most deprived with multimorbidity have 3.26 (CI 2.48 to 4.28) times higher short-term mortality hazard than patients without comorbidities and least deprived. Among DLBCL (table 5), and for the same comparison, the short-term mortality hazard was 1.88 (CI 1.70 to 2.07) times higher at 1 year.

**Table 5:** Linearly combined adjusted HR of comorbidity status with deprivation level on short-term mortality (after multiple imputation) among (A) follicular lymphoma (FL) (deaths at 1 year: n=1087) and (B) diffuse large B-cell lymphoma (DLBCL) (1 year: n=9095) for patients in England from 2005 to 2015

	Comorbidity status		
	None	One	Multimorbidity
	HR* (95% CI)	HR* (95% CI)	HR* (95% CI)
**(A) Follicular**			
Deprivation			
Least deprived	Ref	1.28 (1.01 to 1.61)	2.17 (1.78 to 2.64)
2	1.09 (0.90 to 1.31)	1.39 (1.03 to 1.88)	2.36 (1.80 to 3.10)
3	1.15 (0.95 to 1.39)	1.47 (1.09 to 1.98)	2.50 (1.91 to 3.26)
4	1.44 (1.19 to 1.73)	1.84 (1.36 to 2.47)	3.12 (2.40 to 4.06)
Most deprived	1.50 (1.23 to 1.84)	1.92 (1.42 to 2.59)	3.26 (2.48 to 4.28)
**(B) DLBCL**			
Deprivation			
Least deprived	Ref	1.24 (1.15 to 1.35)	1.44 (1.34 to 1.55)
2	1.05 (0.98 to 1.12)	1.30 (1.18 to 1.44)	1.51 (1.37 to 1.67)
3	1.13 (1.06 to 1.20)	1.40 (1.27 to 1.55)	1.63 (1.48 to 1.80)
4	1.23 (1.15 to 1.31)	1.52 (1.38 to 1.69)	1.78 (1.61 to 1.95)
Most deprived	1.30 (1.21 to 1.40)	1.62 (1.46 to 1.80)	1.88 (1.70 to 2.07)

*Adjusted for sex, ethnicity and the restricted cubic splines of age.

**Figure 2:**
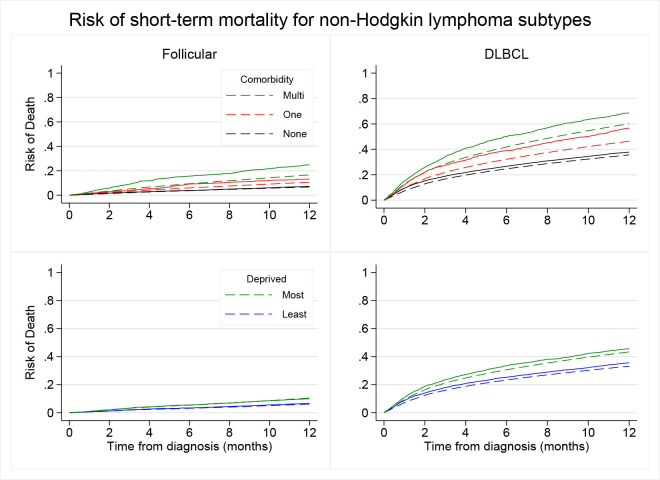
Risk of short-term mortality for follicular lymphoma (FL) (n=14 043) and diffuse large B-cell lymphoma (DLBCL) (n=27 379) by comorbidity status and deprivation level in England between 2005 and 2015 (solid: Aalen-Nelson approach, dash: standardised to the empirical distribution of age, sex and ethnicity).

Figure 2 shows the unadjusted (Nelson-Aalen non-parametric estimate) and standardised risks of death up to 1 year since diagnosis for FL and DLBCL by comorbidity status and deprivation. Online supplemental table S4A, B shows results of complete case and after multiple imputation. Standardised to age, sex, deprivation and ethnicity, the risk of death over the first year was consistently higher among those with multimorbidity compared with those with one comorbidity or none. For both FL and DLBCL, the unadjusted analysis showed that patients with multimorbidity had consistently higher cumulative incidence of death compared with those with one comorbidity or none (log rank test p<0.001).

## Discussion

We aimed to explore the association between comorbidity status, and deprivation and their combination, on short-term mortality for patients with FL or DLBCL. We found that multimorbidity and deprivation and their combined effect are strong independent predictors of short-term mortality among patients with DLBCL and FL in England during 2005–2015.

To our knowledge, this is the first study that investigates the association between multimorbidity, and deprivation, on short-term mortality among patients with DLBCL and FL in England. Despite the scarcity of research within England, our findings are consistent with previous evidence from other countries. A Swedish study found that higher comorbidity status was independently associated with a higher risk of mortality among patients with DLBCL.^31^ Additionally, more deprived, compared with least deprived, patients had a higher risk of DLBCL-related mortality and there was evidence of a significant linear trend across the quintiles of deprivation. A Danish study found that higher comorbidity status was independently associated with shorter survival lengths among patients with any type of NHL (HR 1.60, CI 1.45 to 1.75).^32^ However, these studies used a non-cancer specific comorbidity score, which underperforms (in comparison to cancer-specific scores) when using predictive models for short-term outcomes.^20^ These studies suggest that the effect of comorbidity mainly occurs prior to, and shortly after, cancer diagnosis. Further studies could assess the effect of these prognostic factors on longer term survival from DLBCL or FL, using deprivation-specific life tables to minimise the inaccuracy of expected mortality when life tables are not also stratified by comorbidity status.^33^

As the association between deprivation and NHL survival is not studied as widely as solid tumours, it was unclear whether there was an association between deprivation and short-term mortality for haematological malignancies. Previous studies have described a deprivation gap in survival comparing the least-deprived to most-deprived,^8 10 34^ but have not assessed the association. Although explored for Hodgkin’s lymphoma,^35^ to our knowledge, this is the first study to explore the association between deprivation and short-term mortality for NHL in England: our study provides evidence of a strong and independent association.

There are several dynamics that may explain the association observed in this study. First, the presence of a comorbidity is known to affect the timely diagnosis of DLBLC and FL,^36^ such that comorbidities presenting with similar symptoms to DLBLC or FL may delay the diagnosis and dissimilar symptoms may hasten the diagnosis. Moreover, the prevalence of comorbidities increases with age, and among older patients with DLBCL and FL this prevalence is consistently over 60%,^37 38^ which may partly explain the delay in diagnosis among older ages. Further research is needed to identify comorbidities that alter the timely diagnosis

Second, guidelines of lymphoma management focus on a single-disease standard regimen, but there is little guidance on multidisease management.^39^ A systematic review found the majority of patients with a comorbidity did not receive the standard regimen and were allocated alternative, less-intense treatments.^40^ Cancer care could be improved by defining clear guidelines that recommend a comorbidity-specific treatment regimen and provide an accurate definition, and a measure, of the dose intensity.

Third, differences in access to treatments, or risk of adverse effects, may partly explain the multimorbidity gap in survival from DLBCL and FL; clinicians may abstain from allocating a treatment associated with a higher risk of adverse events because it can exacerbate the complex management of cancer care. Patients without comorbidities, after receiving standard treatment regimens, still experience an increased risk of cardiovascular events.^41^ A first-line standard treatment for DLBCL is a combination of chemotherapy and immunotherapies, such as rituximab, and is known to be effective for those of an advanced age. Rituximab is often used in combination with anthracyclines (eg, doxorubicin), which is associated with an increase in the incidence of adverse events (eg, cardiotoxicity) commonly in the form of congestive heart failure.^42^

Fourth, due to the indolent nature of FL, we cannot rule out that the increase in short-term mortality we observed might be due to causes of death other than the cancer itself (eg, multimorbidity). Moreover, the risk of short-term mortality was more pronounced in FL than DLBCL, which may also indicate that other causes of death (other than the cancer) may play a larger role in FL than DLBCL.

Lastly, the association between deprivation and short-term mortality, that is not explained by patient characteristics, might be explained by the association between deprivation and use of emergency services or population density,^43 44^ or between population density and the use of emergency services.^45^ For example, population dense areas may accumulate high demands that current facilities of healthcare services are unable to accommodate. Therefore, emergency services (eg, emergency diagnostic route), which is associated with a late stage of cancer, may explain the higher mortality hazard observed among more deprived patients. Further research could investigate the demand and availability of healthcare services in densely populated areas.

The strengths of this study include the large sample size within a database of high-quality population-based clinical records with a high national coverage. We linked clinical records with the HES database, which encompasses all patients in England with a diagnosis of DLBCL and FL between 2005 and 2013. The objective data sources provide information on patients that is gathered prospectively. Furthermore, the standardised risk provides an interpretation of the risk of death that is averaged over the entire population.

Due to data availability, our study has some limitations. First, we did not include tumour stage, route to diagnosis (eg, general practitioner referral), or treatment plan; consequently, further research is needed to dissect the effects of comorbidity, stage and treatment on survival. Since tumour stage, route and treatment allocation are considered to be on the causal pathway between comorbidity status and short-term mortality, causal inference mediation analysis is required to estimate the proportion of the effect of comorbidity status on survival that is explained by said mediators.

Second, recent research highlights the interest in using individual-level socioeconomic measures for assessing patient health outcomes in addition to area-level measures of deprivation.^46^ However, information on individual-level socioeconomic measures was unavailable, so we used only an area-level measure of socioeconomic status, which encapsulates the multidimensional composition of a patient’s deprivation level in addition to the contextual level.^21^ Furthermore, there is better concordance between area-level and individual-level measures of education when assessing patient health outcomes.^46^ The observed deprivation level of a patient in our study is likely to be consistent had they been diagnosed at a different time; this is because deprivation scores have a high concordance between updates (ie, IMD of 2007, 2010 and 2015).^21^ Our results are comparable to studies using this area-level measure of deprivation.

Third, HES data contain information on all patients admitted to a hospital (secondary care) in England. It is possible that some comorbidities were not observed because they were diagnosed, and treated, during primary care (eg, general practitioner consultations). However, the RCS’ comorbidity index, among other indices, is constructed based on the impact of the comorbidity on the risk of mortality; in other words, severe comorbidities that require hospitalisation. Comorbidities of the RCS comorbidity index are those that often require hospitalisation, leading to a record within HES data. Previous research has shown that combining primary care records to secondary care data identifies a greater proportion of comorbidity within the population; however, the inclusion of comorbidities identified from primary care records does not have a large effect on predicted cancer survival beyond results obtained using secondary care data.^47^

Fourth, relative, or cause-specific, survival methods were not used in this study, which would have accounted for competing risks of death. Reliable information on the cause of death was unavailable for all patients diagnosed with DLBCL or FL in England in this study. Net survival within the relative survival setting could be an option; however, there are additional methodological issues when combining net survival with causal inference methods and multiple imputation. Moreover, net survival relies on sufficiently stratified lifetables (eg, inclusion of comorbid-specific conditions) to correctly account for the background population mortality, which are not currently available.^33^

Last, as complete case analysis may lead to selection bias, we performed multiple imputation under a missing at random assumption. We obtained the same conclusions under a complete case analysis and after multiple imputation. Since the missing at random assumption is untestable,^48^ further work could conduct a sensitivity analysis to departures from the missing at random assumption, through techniques for imputing under a missing not at random assumption.^49^

In conclusion, multimorbidity and deprivation, combined and independently, are strong predictors of an increased risk of short-term mortality at 1 year since diagnosis among patients with DLBCL or FL in England. Therefore, public health prevention strategies are needed to reduce the short-term mortality gap due to socioeconomic inequalities and comorbidities among patients with NHL.

## Supplementary Material

Author's manuscript

## Data Availability

Data may be obtained from a third party and are not publicly available. The data that support the findings of this study are available via application to the Public Health England Office for Data Release, but restrictions apply to the availability of these data. No additional data available.
