# Supplementary material for: Association between multimorbidity and socioeconomic deprivation on short-term mortality among patients with diffuse large B-cell or follicular lymphoma in England: a nationwide cohort study
Source: BMJ Open. 2021 Nov 30;11(11):e049087. doi: 10.1136/bmjopen-2021-049087 (PMC8634234; doi:10.1136/bmjopen-2021-049087)
Supplement: Supplementary data [file bmjopen-2021-049087supp001.pdf]

Supplementary Tables

This page is intentionally blank. Please move to next page.

Supplementary Table S1. Distribution of non-Hodgkin lymphoma subtypes for patients in England diagnosed from 2005-2013, with respective morphology and topography ICD-O-3 codes.

Index	Site group (subtype)	Progression	Topography	Morphology	n	%
1	CLL/SLL*	Indolent	C82.0-C85.9	9670, 9823	3,875	5.08
2	Waldenstrom macroglobulinemia	Indolent	C82.0-C85.9	9761	2,398	3.14
3	Mantle cell	Indolent	C82.0-C85.9	9673	3,458	4.53
4	Diffuse large B-cell	Aggressive	C82.0-C85.9	9680, 9688, 9737-9738	27,379	35.89
5	Burkitt	Aggressive	C82.0-C85.9	9687, 9826	695	0.91
6	Follicular	Indolent	C82.0-C85.9	9690-9691, 9695, 9698	14,043	18.41
7	Mature T-cell	Aggressive	C82.0-C85.9	9702	5,127	6.72
8	Marginal zone B-cell	Indolent	C82.0-C85.9	9689, 9699, 9760, 9764, 9699	4,277	5.61
**Subtotal**					**61,252**	**80.30**
9	Not Otherwise Specified	n/a	C82.0-C85.9	9591, 9675, 9735	9,581	12.56
10	Other***	n/a	C82.0-C85.9	9591, 9675, 9735	5,449	7.14
**Total**					**76,282**	**100.00****

n/a – not applicable; there was no subtype information
* Chronic lymphocytic leukaemia/Small-cell lymphocytic lymphoma
** Percentages may not equate to 100.0% due to rounding
*** The morphology code specifies these patients are diagnosed with NHL. However, the description states ‘other’; these patients are classified similarly to ‘Not Otherwise Specified’.

Smith MJ, *et al. BMJ Open* 2021; 11:e049087. doi: 10.1136/bmjopen-2021-049087

Supplementary Table S2: Comorbidities and their diagnostic ICD-10 codes

Comorbidity	ICD-10
Myocardial infarction	I21.x, I22.x, I25.2
Congestive heart failure	I11.0, I13.0, I13.2, I25.5, I42.0, I42.5–I42.9, I43.x, I50.x, P29.0
Peripheral vascular disease	I70.x, I71.x, I73.1, I73.8, I73.9, I77.1, I79.0, I79.2, K55.1, K55.8, K55.9, Z95.8, Z95.9
Cerebrovascular disease	G45.x, G46.x, H34.0, I60.x–I69.x
Dementia	F00.x–F03.x, F05.1, G30.x, G31.1
Chronic obstructive pulmonary disease	I27.9, J40.x–J47.x, J60.x–J67.x, J68.4, J70.1, J70.3
Rheumatic disease	M05.x, M06.x, M31.5, M32.x–M34.x, M35.1, M35.3, M36.0
Liver disease	B18.x, K70.0–K70.3, K70.9, K71.3–K71.5, K71.7, K73.x, K74.x, K76.0, K76.2–K76.4, K76.8, K76.9, Z94.4, K71.1, K72.1, K72.9, K76.5, K76.6, K76.7, I85.0, I85.9, I86.4, I98.2, K70.4,
Diabetes without chronic complication	E10.0, E10.1, E10.6, E10.8, E10.9, E11.0, E11.1, E11.6, E11.8, E11.9, E12.0, E12.1, E12.6, E12.8, E12.9, E13.0, E13.1, E13.6, E13.8, E13.9, E14.0, E14.1, E14.6, E14.8, E14.9
Diabetes with chronic complication	E10.7, E11.2–E11.5, E11.7, E12.2–E12.5, E12.7, E13.2–E13.5, E13.7, E14.2–E14.5, E14.7
Hemiplegia or paraplegia	G04.1, G11.4, G80.1, G80.2, G81.x, G82.x, G83.0–G83.4, G83.9
Renal disease	I12.0, I13.1, N03.2–N03.7, N05.2–N05.7, N18.x, N19.x, N25.0, Z49.0–Z49.2, Z94.0, Z99.2
AIDS/HIV	B20.x–B22.x, B24.x

ICD-10: International Classification of Diseases, 10th Revision
Diabetes with/without chronic complication is combined in the RCS Charlson Comorbidity Score

Smith MJ, *et al. BMJ Open* 2021; 11:e049087. doi: 10.1136/bmjopen-2021-049087

Supplementary Table S3. Sociodemographic characteristics, route of diagnosis, comorbidity status and Ann Arbor cancer stage distribution, of Follicular (n=14,043) and DLBCL (n=27,379) lymphomas in England, 2005-2013.

	Follicular	DLBCL		
	N = 14,043	N = 27,379	**OR* (95% CI)**	**p-value**
**Age** *(mean; SD)***	66.7 (11.0)	70.8 (11.3)	1.37 (1.35 – 1.40)	<0.001
**Gender**				
Male	6,512 (46.4)	14,652 (53.5)	Ref	Ref
Female	7,531 (53.6)	12,727 (46.5)	0.75 (0.72 - 0.78)	<0.001
**Deprivation**				
Least deprived	3,284 (23.4)	5,895 (21.5)	Ref	Ref
2	3,228 (23.0)	6,238 (22.8)	1.08 (1.01 – 1.14)	0.016
3	3,013 (21.5)	5,756 (21.0)	1.06 (1.00 – 1.13)	0.047
4	2,609 (18.6)	5,341 (19.5)	1.14 (1.07 – 1.22)	<0.001
Most deprived	1,909 (13.6)	4,149 (15.2)	1.21 (1.13 – 1.30)	<0.001
**Comorbidity status**				
Non	12,898 (91.9)	24,269 (88.6)	Ref	Ref
One	623 (4.4)	1,538 (5.6)	1.60 (1.45 – 1.77)	<0.001
Multimorbidity	522 (3.7)	1,572 (5.7)	1.58 (1.43 – 1.75)	<0.001
**Route**				
Elective	11,211 (86.7)	17,280 (66.3)	Ref	Ref
Emergency	1,727 (13.4)	8,799 (33.7)	3.31 (3.12 – 3.50)	<0.001
Missing	*1,105 (7.9)*	*1,300 (4.8)*	-	-
**Stage**				
I	848 (26.6)	1,680 (27.8)	Ref	Ref
II	473 (14.8)	1,169 (19.3)	1.25 (1.09 – 1.43)	0.001
III	822 (25.7)	1,046 (17.3)	0.64 (0.57 – 0.73)	<0.001
IV	1050 (32.9)	2,152 (35.6)	1.03 (0.93 – 1.16)	0.548
Missing	*10,850 (77.3)*	*21,332 (77.9)*	-	-
**Ethnicity**				
White	10,110 (95.6)	20,204 (95.4)	Ref	Ref
Other	461 (4.4)	984 (4.6)	1.07 (0.95 – 1.20)	0.254
Missing	*3,472 (24.7)*	*6,191 (22.6)*	-	-

* Odds ratio from a complete case analysis comparing the odds of DLBCL to Follicular lymphoma
** 10-year increase in age
P-values calculated from Wald tests

Smith MJ, *et al. BMJ Open* 2021; 11:e049087. doi: 10.1136/bmjopen-2021-049087

Supplementary Table S4a: Risk of short-term mortality amongst patients diagnosed with Follicular lymphomas (n=14,043) by comorbidity status in England between 2005 and 2013

	**Complete Case Analysis**						**After Multiple Imputation**					
	**Comorbidity status**						**Comorbidity status**					
	**None**		**One**		**Multimorbidity**		**None**		**One**		**Multimorbidity**	
**Month**	*CH*	*95% CI*	*CH*	*95% CI*	*CH*	*95% CI*	*CH*	*95% CI*	*CH*	*95% CI*	*CH*	*95% CI*
1	0.6	0.4 – 0.9	1.0	0.7 - 1.4	1.6	1.0 – 2.3	0.8	0.7 – 1.0	1.3	1.1 – 1.5	2.4	1.6 – 3.2
2	1.4	1.0 – 1.7	2.2	1.6 - 2.7	3.5	2.3 – 4.6	1.7	1.5 – 1.9	2.6	2.2 – 3.0	4.8	3.4 – 6.3
3	2.0	1.6 – 2.4	3.2	2.6 - 3.9	5.2	3.6 – 6.7	2.4	2.2 – 2.7	3.7	3.2 – 4.2	6.8	4.9 – 8.7
4	2.6	2.2 – 3.1	4.2	3.5 - 4.9	6.7	4.8 – 8.6	3.0	2.7 – 3.3	4.6	4.0 – 5.2	8.4	6.1 – 10.8
5	3.2	2.6 – 3.7	5.1	4.3 - 5.9	8.1	5.9 – 10.3	3.6	3.3 – 3.9	5.4	4.8 – 6.1	9.9	7.2 – 12.6
6	3.7	3.1 – 4.3	5.9	5.0 - 6.9	9.4	6.9 – 11.9	4.1	3.8 – 4.4	6.2	5.5 – 7.0	11.3	8.3 – 14.3
7	4.2	3.6 – 4.9	6.7	5.7 - 7.7	10.6	7.8 – 13.4	4.6	4.2 – 5.0	7.0	6.2 – 7.9	12.7	9.3 – 16.0
8	4.7	4.0 – 5.5	7.5	6.4 - 8.6	11.8	8.8 – 14.9	5.1	4.8 – 5.5	7.8	6.9 – 8.8	14.1	10.4 – 17.8
9	5.2	4.5 – 6.0	8.3	7.2 - 9.4	13.1	9.7 – 16.4	5.7	5.3 – 6.1	8.7	7.6 – 9.7	15.5	11.5 – 19.5
10	5.7	5.0 – 6.5	9.1	7.9 - 10.3	14.3	10.6 – 17.9	6.2	5.8 – 6.7	9.5	8.4 – 10.6	17.0	12.6 – 21.3
11	6.2	5.4 – 7.0	9.9	8.6 - 11.1	15.4	11.5 – 19.4	6.8	6.4 – 7.3	10.3	9.2 – 11.5	18.4	13.7 – 23.1
12	6.7	5.9 – 7.5	10.6	9.2 - 12.0	16.6	12.3 – 20.9	7.4	6.9 – 7.8	11.2	9.9 – 12.4	19.8	14.8 – 24.9

CH – cumulative hazard of death (e x 10^2)
95% CI – confidence interval (e x 10^2)

Supplementary Table S4b: Risk of short-term mortality amongst patients diagnosed with DLBCL (n=27,379) by comorbidity status in England between 2005 and 2013

	**Complete Case Analysis**						**After Multiple Imputation**					
	**Comorbidity status**						**Comorbidity status**					
	**None**		**One**		**Multimorbidity**		**None**		**One**		**Multimorbidity**	
**Month**	*CH*	*95% CI*	*CH*	*95% CI*	*CH*	*95% CI*	*CH*	*95% CI*	*CH*	*95% CI*	*CH*	*95% CI*
1	7.4	6.9 – 7.9	9.7	9.0 – 10.4	13.2	11.5 – 14.9	9.2	8.9 – 9.6	12.0	11.4 – 12.5	16.1	14.5 – 17.7
2	12.9	12.2 – 13.7	17.0	16.0 – 18.0	23.0	20.4 – 25.7	15.3	14.8 – 15.7	19.7	18.8 – 20.5	26.3	23.9 – 28.8
3	16.8	16.0 – 17.6	22.0	20.8 – 23.2	30.0	26.6 – 33.3	19.1	18.6 – 19.7	24.6	23.6 – 25.6	32.8	29.8 – 35.8
4	19.8	18.9 – 20.7	25.9	24.5 – 27.2	35.4	31.5 – 39.3	22.1	21.6 – 22.7	28.4	27.2 – 29.5	37.7	34.3 – 41.1
5	22.4	21.4 – 23.3	29.2	27.7 – 30.7	39.9	35.6 – 44.3	24.8	24.1 – 25.4	31.7	30.4 – 32.9	42.0	38.2 – 45.8
6	24.7	23.7 – 25.7	32.2	30.6 – 33.8	43.9	39.2 – 48.7	27.1	26.5 – 27.8	34.7	33.3 – 36.0	45.9	41.8 – 50.0
7	26.8	25.8 – 27.8	34.9	33.3 – 36.6	47.5	42.4 – 52.6	29.3	28.6 – 30.0	37.5	36.0 – 38.9	49.6	45.2 – 53.9
8	28.8	27.7 – 29.8	37.5	35.8 – 39.2	50.8	45.4 – 56.2	31.4	30.7 – 32.1	40.1	38.5 – 41.6	53.0	48.3 – 57.6
9	30.6	29.5 – 31.7	39.9	38.1 – 41.7	53.8	48.2 – 59.5	33.3	32.6 – 34.0	42.5	40.9 – 44.1	56.2	51.2 – 61.1
10	32.4	31.2 – 33.5	42.2	40.3 – 44.0	56.7	50.8 – 62.6	35.1	34.4 – 35.9	44.8	43.2 – 46.5	59.2	54.0 – 64.4
11	34.0	32.8 – 35.2	44.3	42.4 – 46.3	59.4	53.3 – 65.5	36.9	36.1 – 37.7	47.1	45.3 – 48.8	62.1	56.7 – 67.6
12	35.6	34.4 – 36.9	46.4	44.3 – 48.5	62.0	55.6 – 68.4	38.6	37.7 – 39.4	49.2	47.4 – 51.0	64.9	59.2 – 70.6

CH – cumulative hazard (e x 10^2)
95% CI – confidence interval (e x 10^2)

Smith MJ, *et al. BMJ Open* 2021; 11:e049087. doi: 10.1136/bmjopen-2021-049087
