# Supplementary material for: Association between multimorbidity and socioeconomic deprivation on short-term mortality among patients with diffuse large B-cell or follicular lymphoma in England: a nationwide cohort study
Source: BMJ Open. 2021 Nov 30;11(11):e049087. doi: 10.1136/bmjopen-2021-049087 (PMC8634234; doi:10.1136/bmjopen-2021-049087)
Supplement: Author's manuscript [file bmjopen-2021-049087.draft_revisions.pdf]

BMJ Open is committed to open peer review. As part of this commitment we make the peer review history of every article we publish publicly available.

When an article is published we post the peer reviewers' comments and the authors' responses online. We also post the versions of the paper that were used during peer review. These are the versions that the peer review comments apply to.

The versions of the paper that follow are the versions that were submitted during the peer review process. They are not the versions of record or the final published versions. They should not be cited or distributed as the published version of this manuscript.

BMJ Open is an open access journal and the full, final, typeset and author-corrected version of record of the manuscript is available on our site with no access controls, subscription charges or pay-per-view fees (http://bmjopen.bmj.com).

If you have any questions on BMJ Open's open peer review process please email info.bmjopen@bmj.com

BMJ Open

Association between multimorbidity and socioeconomic deprivation on short-term mortality amongst patients with Diffuse Large B-cells or Follicular lymphomas in England

Journal:	*BMJ Open*
Manuscript ID	bmjopen-2021-049087
Article Type:	Original research
Date Submitted by the Author:	18-Jan-2021
Complete List of Authors:	Smith, Matthew; London School of Hygiene & Tropical Medicine, Non-Communicable Disease Epidemiology<br>Njagi, Edmund Njeru; London School of Hygiene & Tropical Medicine<br>Belot, Aurelien; London School of Hygiene & Tropical Medicine<br>Leyrat, Clémence; London School of Hygiene & Tropical Medicine, Department of Non-Communicable Disease Epidemiology<br>Bonaventure, Audrey; University of Paris, Epidemiology of Childhood and Adolescent Cancers Team<br>Benitez Majano, Sara ; London School of Hygiene & Tropical Medicine, Department of Non-Communicable Disease Epidemiology<br>Rachet, Bernard; London School of Hygiene & Tropical Medicine, Non-Communicable Disease Epidemiology<br>Luque-Fernandez, Miguel-Angel; London School of Hygiene and Tropical Medicine, Epidemiology and Population Health; Instituto de Investigación Biosanitaria de Granada, Noncommunicable Disease and Cancer Epidemiology Group
Keywords:	Epidemiology < ONCOLOGY, STATISTICS & RESEARCH METHODS, Lymphoma < HAEMATOLOGY

SCHOLARONE™
Manuscripts

I, the Submitting Author has the right to grant and does grant on behalf of all authors of the Work (as defined in the below author licence), an exclusive licence and/or a non-exclusive licence for contributions from authors who are: i) UK Crown employees; ii) where BMJ has agreed a CC-BY licence shall apply, and/or iii) in accordance with the terms applicable for US Federal Government officers or employees acting as part of their official duties; on a worldwide, perpetual, irrevocable, royalty-free basis to BMJ Publishing Group Ltd ("BMJ") its licensees and where the relevant Journal is co-owned by BMJ to the co-owners of the Journal, to publish the Work in this journal and any other BMJ products and to exploit all rights, as set out in our licence.

The Submitting Author accepts and understands that any supply made under these terms is made by BMJ to the Submitting Author unless you are acting as an employee on behalf of your employer or a postgraduate student of an affiliated institution which is paying any applicable article publishing charge ("APC") for Open Access articles. Where the Submitting Author wishes to make the Work available on an Open Access basis (and intends to pay the relevant APC), the terms of reuse of such Open Access shall be governed by a Creative Commons licence – details of these licences and which Creative Commons licence will apply to this Work are set out in our licence referred to above.

Other than as permitted in any relevant BMJ Author's Self Archiving Policies, I confirm this Work has not been accepted for publication elsewhere, is not being considered for publication elsewhere and does not duplicate material already published. I confirm all authors consent to publication of this Work and authorise the granting of this licence.

For peer review only

Title

Association between multimorbidity and socioeconomic deprivation on short-term mortality amongst patients with Diffuse Large B-cells or Follicular lymphomas in England

Authors

Matthew J. Smith[1], Edmund Njeru Njagi[1], Aurélien Belot[1], Clémence Leyrat[1,2], Audrey Bonaventure[3], Sara Benitez Majano[1], Bernard Rachet[1], Miguel Angel Luque Fernandez[1,4,5*]

Authors' affiliations

[1] Inequalities in Cancer Outcomes Network, Department of Non-Communicable Disease Epidemiology, London School of Hygiene and Tropical Medicine, Keppel Street, London, WC1E 7HT, UK

[2] Department of Medical Statistics, London School of Hygiene and Tropical Medicine, Keppel Street, London, WC1E 7HT, UK

[3] CRESS, Université de Paris, INSERM, UMR 1153, Epidemiology of Childhood and Adolescent Cancers Team, Villejuif, France

[4] Noncommunicable Disease and Cancer Epidemiology Group, Instituto de Investigación Biosanitaria de Granada, Ibs.GRANADA, Andalusian School of Public Health, Granada, Spain

[5] Centro de Investigación Biomédica en Red de Epidemiología y Salud Pública (CIBER of Epidemiology and Public Health, CIBERESP), Madrid, Spain

Corresponding author*

Miguel Angel Luque-Fernandez
Keppel Street, London, WC1E 7HT, UK
ORCID: 0000-0001-6683-5164
Email: *miguel-angel.luque@lshtm.ac.uk*

Word count: Abstract: 189; Text: 2807; Tables: 4; Figures: 1

For peer review only

Abstract

Objectives: We aimed to assess the association between multimorbidity and deprivation on short-term mortality amongst DLBCL and FL patients in England.

Setting: The association of multimorbidity and socioeconomic deprivation on survival among patients diagnosed with Diffuse Large B-cell (DLBCL) and Follicular lymphoma (FL) in England between 2005 and 2013. We linked the English population-based cancer registry with electronic health records databases and estimated adjusted mortality rate ratios by multimorbidity and deprivation status. Using flexible hazard-based regression models, we computed DLBCL and FL standardised mortality risk by deprivation and multimorbidity at 1 year.

Results: Overall, 41,422 patients aged 45-99 years were diagnosed with DLBCL or FL in England during 2005-2015. Most deprived FL patients with multimorbidities had three times higher hazard (HR: 3.3, CI: 2.48 – 4.28) than least deprived patients without comorbidity; amongst DLBCL there was approximately twice the hazard (HR: 1.9, CI: 1.70– 2.07).

Conclusions: Multimorbidity, deprivation, and their combination, are strong and independent predictors of an increased short-term mortality risk amongst DLBCL and FL patients in England. Public health measures targeting the reduction of multimorbidity amongst most deprived DLBCL and FL patients are needed to reduce the short-term mortality gap.

Key words: Cancer epidemiology, Diffuse Large B-cell lymphoma, Follicular lymphoma, multimorbidity, deprivation, survival analysis

For peer review only

Strengths and limitations of this study

- Comorbidity and multimorbidity are independent factors of short-term survival amongst patients with Diffuse Large B-cell Lymphoma (DLBCL) or Follicular Lymphoma (FL) in England.
- Comorbidity and multimorbidity only partly explain the socioeconomic gap in short-term survival in patients with DLBCL or FL.
- Further research is needed in explaining the remaining inequalities in cancer survival, and warrants questions regarding the role of the health care system in explaining inequalities.

For peer review only

Introduction

In England, non-Hodgkin lymphoma (NHL) is the sixth most commonly diagnosed cancer with an incidence rate of 22.7.[1] Apart from lung cancer, survival estimates of NHL (79.4% survival probability at 1 year) are amongst the lowest of the six most common cancers.[2,3] NHL encompasses a heterogeneous group of malignancies with diverse histological patterns; in addition, the commonest NHL subtypes are diffuse large B-cell lymphoma (DLBCL) and follicular lymphoma (FL),[4] patients of which show a large variation in survival.[5] Cancer survival in England is lower than other European countries,[6] but is similar when restricting to those surviving after 1 year:[7] identifying, and influencing, the factors of short-term mortality could reduce the gap in survival.

Over the past decades, patient survival of FL has steadily improved and stagnated for DLBCL;[3] furthermore, disparities in survival between deprivation levels remains.[8] Public health policies have increased awareness and set targets, such as minimising the length of time from referral to treatment, to reduce the inequalities;[9] however, since their implementation, there has been no evidence that the National Health Service (NHS) Cancer Plan had an impact on the inequalities in cancer survival.[10] The deprivation-gap in survival is still apparent, despite the NHS Cancer Plan and successive policies.[9]

Comorbidities, which refers to the presence of a long-term health condition additional, but unrelated, to the underlying cancer,[11] tends to mask cancer symptoms, delaying cancer diagnosis and decreases survival.[12] Older age groups and those living in more deprived areas experience more comorbidities. With a global aging population, the prevalence of comorbidities is expected to increase.[13] The association between multimorbidity and survival is described for other cancers,[14] but for DLBCL and FL this relationship remains unclear.

We aim to identify the association between multimorbidity and risk of short-term mortality amongst DLBCL and FL patients in England. We hypothesise that multimorbidity and deprivation, independently and combined, contribute to an increased risk of death after DLBCL or FL diagnosis.

For peer review only

Methods

Study Design, participants, data and setting

We used the data from a retrospective population-based cohort study with DLBCL and FL patients diagnosed between 1st January 2005 and 31st December 2013 and followed up to 31st December 2015. DLBCL and FL diagnoses were made according to the International Classification of Diseases for Oncology, 3rd edition, based on codes C82.0-C85.9[15] (**Supplementary Table S1** shows the subtype categorisation). Patients entered the study on the date of their diagnosis and were followed up until death or censored at 1 year, whichever occurred first.

Data was obtained from population-based cancer registries within the English National Cancer Registry and Analysis Service (CAS)[16] and linked to patient's electronic health records from Hospital Episode Statistics (HES). CAS contains patient and tumour variables including relevant dates (birth, diagnosis, and vital status), sex, age at diagnosis, deprivation, cancer site and morphology. We used population based administrative hospital discharge data for the assessment of comorbid conditions; we analysed HES data (containing comorbid conditions records) according to the International Classification of Diseases, 10th revision (**Supplementary Table S2**), for the period 2003 to 2015. HES contains clinical, administrative, and demographic information about individual patients. To avoid selection bias including cancer related comorbidities, we restricted retrospective records of comorbidities to occurring between 6- and 24-months prior to the date of DLBCL and FL diagnosis.[17]

Patient and public involvement

No patient or public involvement.

For peer review only

Outcome, exposure and other variables

The outcome of this study was the time since diagnosis up to death observed within the first year after diagnosis of DLBLC and FL patients (patients alive were censored at survival time defined with the date of last known vital status), the main exposures were multimorbidity status and deprivation. Due to data availability and clinical reasoning, we include as confounders age, gender, and ethnicity. Due to the positivity assumption, and the chances of having a multimorbidity, we included patients aged above 45 years at diagnosis (**Figure 2**).

Comorbidity status was classified according to the Royal College of Surgeons (RCS) Charlson score (an adaptation of the Charlson comorbidity index[18]) that includes 12 categories for comorbidities, excludes a category (peptic ulcer disease) and groups diseases together (e.g. diabetes mellitus codes with or without complications were grouped into a single category). The score was categorised into those with none, one comorbidity (whatever the type), or two or more comorbidities (defined as *multimorbidity*). The score does not weight the comorbidities assuming that any comorbidity has the same impact on short-term mortality.[19]

Area-level deprivation was categorised into one of five quintiles (5th is most deprived). Deprivation was used as a proxy of individual level socioeconomic status. We used the Index of Multiple Deprivation[20] (IMD), which is an area-level deprivation score based on the Lower Super Output Area[21] (*LSOA*) residence of the patient at the time of cancer diagnosis. LSOA is a geographical location with a median of 1500 inhabitants.

Ethnicity due to data sparsity amongst ethnic minorities was recorded as either white or other. Route to diagnosis (CAS dataset), although not considered in our analysis because it was on the causal pathway, is included in the imputation models for missing data.

Statistical Analysis

We described the characteristics of DLBLC and FL patients using counts and proportions (**Supplementary Table S3**). We assessed the unadjusted association between having multimorbidity and patients' characteristics, using chi-square tests._Then, we compute the number of deaths, person-time at risk, and unadjusted rates of

For peer review only

deaths per 100 person-years and rate ratios with 95% confidence intervals (CI) by patients' characteristics by DLBCL and FL subtypes.

The follow-up time for those who died is from the date of diagnosis until death, for those alive it was until administrative censoring at 12 months (no lost to follow-up before 12 months was observed). To describe the multimorbidity and deprivation short-term mortality risk amongst DLBCL and FL patients, we computed the one year cumulative hazard obtained using the non-parametric Nelson-Aalen estimator.[22] Then, we computed adjusted short-term mortality risk by patient characteristics using a flexible parametric modelling approach to model the non-linear change in mortality risk over one year. We included restricted-cubic spline to model the baseline hazard,[23] with three knots located at the 25th, 50th, and 75th percentiles of the log event times. To define the model, let t be the time since DLBCL or FL diagnosis until death or censoring. We define the log cumulative hazard as

$$\ln\left[H(t \mid \boldsymbol{x}_i, A_i, Dep_i)\right] = \gamma_0 + \gamma_1 z_{1i} + \gamma_2 z_{2i} + \gamma_3 A_{1i} + \gamma_4 A_{2i} + \sum_{k=2}^{5} \beta_{0k} \cdot Dep_{ik} + \sum_{m=1}^{M} \boldsymbol{\beta}_m x_{im}$$

where z_i are the indicators for the three knots of the baseline restricted cubic splines. The model specification included the restricted cubic splines for the continuous variable age are given by A_i where the knots are placed at 1 and 6 months, deprivation, and the vector x_{im} of categorical covariates (i.e., multimorbidity, ethnicity and gender). From the model, we derived the cumulative incidence of death at 1 year by comorbidity status (i.e. none, one, or multimorbidity) standardized to the empirical distribution of age, gender, ethnicity and deprivation.[24,25]

We used the same modelling approach to evaluate the linearly combined effect of multimorbidity and derivation on short-term mortality, assuming the effect of multimorbidity is constant across levels of deprivation.[26] We assume that both effects (multimorbidity and deprivation) are independently associated with the mortality rate and that their effect is constant and the rate is increasing linearly.[34]

For peer review only

In sensitivity analysis, we evaluated the robustness of our results to the ethnicity missing data by utilising multiple imputation using chained equations (MICE). We assumed that the missing covariate values were missing at random, given age, gender, deprivation and comorbidity status. We include route of cancer diagnosis and Ann Arbor[27] cancer stage as partially observed auxiliary variables because these variables were predictive of the probability of missing values (established via the missing data mechanism model), and are predictive of the underlying values themselves (established from clinical and epidemiological reasoning).[28] We generated 20 imputed datasets. The imputation model for the partially observed variable ethnicity was defined as a logistic regression model including all the other explanatory variables in the substantive model, the vital status and Nelson-Aalen estimate of the cumulative hazard, and the auxiliary variables (route to, and stage at, diagnosis). We then fit the substantive model to each of the 20 imputed datasets, from these estimates we predict the standardised survival (using *stpm2* package), derived the cumulative hazard (-log[Survival probability]) for each imputed dataset and combined the survival probability estimates using Rubin's rules.[29]

Analyses were performed in Stata version 16 (StataCorp, College Station, Texas, U.S.); the *stpm2* package was used to estimate flexible parametric survival models, and the *standsurv* command to compute standardized mortality risks. The *mi impute* command was used for multiple imputation and the *mi estimate* command to combine estimates.

Results

Overall, 41,422 patients in England, aged from 45 to 99 years, were diagnosed with either DLBCL or FL between 2005 and 2013 in England. Of 14,043 patients with FL, those who died within 1-year, compared to those alive after 1-year, had approximately 3.5 the risk of multimorbidity (11.8% vs. 3.2%, respectively) (**Table 1**); of 27,379 patients diagnosed with DLBCL, this comparison was twice the risk (9.4% vs. 4.5%, respectively). For both DLBCL and FL subtypes, the risk of multimorbidity was higher amongst those living in more deprived areas. Only for patients with DLBCL was there a higher proportion of having any comorbidity amongst males: for patients with FL, this proportion was higher amongst females. As expected, multimorbidity was more prevalent amongst older age groups.

For peer review only

Table 1: Vital status, age, sex, deprivation level and ethnicity according to the comorbidity status amongst n = 41,422 patients with non-Hodgkin lymphoma in England between 2005 and 2013 (27,379 DLBCL cases and 14,043 FL cases).

	**DLBCL comorbidity status** (n = 27,379)			**FL comorbidity status** (n = 14,043)		
	**None**	**One**	**Multi**	**None**	**One**	**Multi**
	**N (%)**	**N (%)**	**N (%)**	**N (%)**	**N (%)**	**N (%)**
**Vital status at 1 year**						
Alive	16,621 (68.5)	872 (56.7)	791 (50.3)	12,003 (93.1)	546 (87.6)	407 (78.0)
Dead	7,648 (31.5)	666 (43.3)	781 (49.7)	895 (6.9)	77 (12.4)	115 (22.0)
**Sex**						
Male	12,904 (53.2)	794 (51.6)	954 (60.7)	5,980 (46.4)	275 (44.1)	257 (49.2)
Female	11,365 (46.8)	744 (48.4)	618 (39.3)	6,918 (53.6)	348 (55.9)	265 (50.8)
**Age at diagnosis (y)**						
45-54	2,685 (11.1)	80 (5.2)	96 (6.1)	2,246 (17.4)	42 (6.7)	33 (6.3)
55-64	5,154 (21.2)	257 (16.7)	200 (12.7)	3,769 (29.2)	140 (22.5)	82 (15.7)
65-74	7,337 (30.2)	432 (28.1)	439 (27.9)	3,952 (30.6)	199 (31.9)	159 (30.5)
75+	9,093 (37.5)	769 (50.0)	837 (53.2)	2,931 (22.7)	242 (38.8)	248 (47.5)
**Deprivation**						
Least deprived	5,348 (22.0)	291 (18.9)	256 (16.3)	3,091 (24.0)	113 (18.1)	80 (15.3)
2	5,586 (23.0)	318 (20.7)	334 (21.3)	3,025 (23.5)	122 (19.6)	81 (15.5)
3	5,115 (21.1)	324 (21.1)	317 (20.2)	2,759 (21.4)	136 (21.8)	118 (22.6)
4	4,665 (19.2)	337 (21.9)	339 (21.6)	2,356 (18.3)	123 (19.7)	130 (24.9)
Most deprived	3,555 (14.7)	268 (17.4)	326 (20.7)	1,667 (12.9)	129 (20.7)	113 (21.7)
**Ethnicity**						
White	17,831 (95.5)	1,204 (96.6)	1,169 (92.6)	9,226 (95.7)	498 (96.3)	386 (92.8)
Other	848 (4.5)	43 (3.5)	93 (7.4)	412 (4.3)	19 (3.7)	30 (7.2)

Missing values: Ethnicity n(%): DLBCL = 6,191 (22.6%), FL = 3,472 (24.7%)

Tables 2a and 2b show the person-time at risk and unadjusted mortality rate of death for DLBLC and FL at one year after diagnosis. Amongst FL and DBLCL patients, 1087 (7.7%) and 9,095 (33.2%) died before 1 year, respectively (**Table** 2a and 2b**)**. Amongst FL, those with one comorbidity or multimorbidity had 2 or 3.5 times the mortality rate, respectively, compared to those with no comorbidity. Amongst DLBCL, those with one comorbidity or multimorbidity had 1.5 or 2 times the mortality rate, compared to no comorbidity.

For peer review only

Table 2A: One-year unadjusted mortality rates and rate ratios by sex, age, deprivation, ethnicity, and comorbidity status amongst patients with **Follicular lymphoma** in England between 2005-2015 (n = 14,043; 1,087 deaths at one-year).

	Deaths / person years	Mortality rate* (95% CI)	Mortality RR	95% CI	p-value
**One-year mortality (n = 1,087)**					
**Sex**					
Male	526/6218.12	8.5 (7.77 – 9.21)	Ref		
Female	561/7225.93	7.8 (7.15 – 8.43)	0.92	(0.82 – 1.03)	0.157
**Age at diagnosis (y)** †					
10-year increase	-	-	2.20	(2.08 – 2.34)	<0.001
**Age at diagnosis (y)**					
45-54	46/2300.66	2.0 (1.50 – 2.67)	Ref		
55-64	129/3926.24	3.3 (2.77 – 3.90)	1.64	(1.17 – 2.30)	0.004
65-74	274/4157.52	6.6 (5.86 – 7.42)	3.30	(2.41 – 4.50)	<0.001
75+	638/3059.62	20.9 (19.30 – 22.54)	10.43	(7.73 – 14.07)	<0.001
**Deprivation**					
Least deprived	211/3175.55	6.7 (5.81 – 7.60)	Ref		
2	227/3102.18	7.3 (6.43 – 8.33)	1.10	(0.91 – 1.33)	0.313
3	230/2884.20	8.0 (7.01 – 9.08)	1.20	(1.00 – 1.45)	0.055
4	240/2471.30	9.7 (8.56 – 11.02)	1.46	(1.23 – 1.76)	<0.001
Most deprived	179/1810.81	9.9 (8.54 – 11.45)	1.49	(1.22 – 1.82)	<0.001
**Ethnicity**					
White	742/9712.99	7.6 (7.11 – 8.21)	Ref		
Other	21/450.96	4.7 (3.04 – 7.14)	0.61	(0.40 – 0.94)	0.024
**Comorbidity status**					
None	895/12412.44	7.5 (7.03 – 7.97)	Ref		
One	77/577.89	13.3 (10.66 – 16.66)	1.85	(1.46 – 2.33)	<0.001
Multimorbidity	115/453.71	25.4 (21.11 – 30.43)	3.52	(2.90 – 4.27)	<0.001

* per 100 person-years
† continuous form of age (for each 10-year increase in age)
CI: Confidence interval, **RR:** Rate Ratio, **Missing values:** ethnicity n(%) = 3,472 (24.7%)

For peer review only

Table 2B: One-year unadjusted mortality rates by sex, age, deprivation, ethnicity, and comorbidity status amongst patients with **DLBCL** in England between 2005-2015 (n = 27,379; 9,095 deaths at One-year).

	Deaths/person years	Mortality rate* (95% CI)	Mortality RR	95% CI	p-value
**One-year mortality (n = 9,095)**					
**Sex**					
Male	4867/11318.43	43.0 (41.81 – 44.23)	Ref	Ref	Ref
Female	4228/9784.59	43.2 (41.93 – 44.53)	1.01	(0.96 – 1.05)	0.817
**Age at diagnosis (y)** †					
10-year increase	-	-	1.50	(1.47 – 1.53)	<0.001
**Age at diagnosis (y)**					
45-54	430/2615.81	16.4 (14.96 – 18.07)	Ref	Ref	Ref
55-64	1074/4937.54	21.8 (20.49 – 23.09)	1.32	(1.18 – 1.48)	<0.001
65-74	2365/6604.47	35.8 (34.39 – 37.28)	2.18	(1.97 – 2.41)	<0.001
75+	5226/6945.19	75.2 (73.23 – 77.31)	4.58	(4.15 – 5.05)	<0.001
**Deprivation**					
Least deprived	1765/4684.43	37.7 (35.96 – 38.48)	Ref		
2	1955/4898.09	39.9 (38.18 – 41.72)	1.06	(0.99 – 1.13)	0.079
3	1948/4424.61	44.0 (42.11 – 46.03)	1.17	(1.10 – 1.25)	<0.001
4	1908/4017.48	47.5 (45.41 – 49.67)	1.26	(1.18 – 1.35)	<0.001
Most deprived	1519/3078.40	49.3 (46.92 – 51.89)	1.31	(1.22 – 1.40)	<0.001
**Ethnicity**					
White	6351/15900.73	39.9 (38.97 – 40.94)	Ref		
Other	270/808.61	33.4 (29.64 – 37.62)	0.84	(0.74 – 0.94)	0.004
**Comorbidity status**					
None	7648/19007.45	40.2 (39.35 – 41.15)	Ref		
One	666/1081.20	61.6 (57.09 – 66.46)	1.53	(1.41 – 1.66)	<0.001
Multimorbidity	781/1014.36	77.0 (71.78 – 82.59)	1.91	(1.78 – 2.06)	<0.001

* per 100 person-years
† continuous form of age (for each 10-year increase in age)
CI: Confidence interval, **RR**: Rate Ratio, **Missing values**: ethnicity n(%) = 6,191 (22.6%)

Table 3 shows, before and after multiple imputation, the mortality hazard amongst DLBCL and FL patients at 1 year adjusted for comorbidity status, sex, age, deprivation and ethnicity. After multiple imputation, for both FL and DLBCL, patients with multimorbidity had a higher mortality hazard compared to those with a single comorbidity or none. Patients in more deprived areas, compared to less deprived, had a higher mortality hazard over the first year. There was evidence of a linear trend in mortality hazard by deprivation level for FL (p<0.001) and DLBCL (p<0.001). The direction and magnitude of the hazard ratios after multiple imputation were similar to complete case analysis.

For peer review only

Table 3: Adjusted hazard ratios of death (before and after multiple imputation) for all patient characteristics amongst patients with (**A**) Follicular or (**B**) DLBCL in England between 2005-2015.

	**Complete Case**			**After multiple imputation**		
	**HR***	**95% CI**	**p-value**	**HR***	**95% CI**	**p-value**
**(A) Follicular**						
**Gender**						
Male	Ref	Ref	-	Ref	Ref	-
Female	1.08	0.85 – 1.38	0.540	0.84	0.74 – 0.94	<0.001
**Comorbidity status**						
None	Ref	Ref	-	Ref	Ref	-
One	1.52	1.15 – 2.03	<0.001	1.28	1.01 – 1.61	<0.041
Multimorbidity	2.36	1.85 – 3.02	<0.001	2.17	1.78 – 2.64	<0.001
**Deprivation**†						
Least	Ref	Ref	-	Ref	Ref	-
2	1.03	0.70 – 1.53	0.873	1.09	0.90 – 1.31	0.378
3	1.47	1.00 – 2.16	0.051	1.15	0.95 – 1.39	0.143
4	1.30	0.87 – 1.94	0.200	1.44	1.19 – 1.73	<0.001
Most	1.63	1.11 – 2.41	0.013	1.50	1.23 – 1.84	<0.001
**Ethnicity**						
White	Ref	Ref	-	Ref	Ref	-
Other	0.49	0.27 – 0.88	0.017	0.64	0.40 – 1.01	0.053
**(B) Diffuse large B-cell**						
**Gender**						
Male	Ref	Ref	-	Ref	Ref	-
Female	0.91	0.84 – 0.99	0.170	0.90	0.86 – 0.93	<0.001
**Comorbidity status**						
None	Ref	Ref	-	Ref	Ref	-
One	1.29	1.18 – 1.42		1.24	1.15 – 1.35	<0.001
Multimorbidity	1.61	1.47 – 1.76	<0.001	1.44	1.34 – 1.55	<0.001
**Deprivation**‡						
Least	Ref	Ref	-	Ref	Ref	-
2	1.17	1.03 – 1.33	0.013	1.05	0.98 – 1.12	0.155
3	1.15	1.01 – 1.30	0.029	1.13	1.06 – 1.20	<0.001
4	1.25	1.11 – 1.42	<0.001	1.23	1.15 – 1.31	<0.001
Most	1.32	1.16 – 1.51	<0.001	1.30	1.21 – 1.40	<0.001
**Ethnicity**						
White	Ref	Ref	-	Ref	Ref	-
Other	0.93	0.77 – 1.13	0.463	1.03	0.91 – 1.16	0.678

CI: Confidence interval, **HR**: Hazard Ratio
Missing values: **(A)** ethnicity n(%) = 3,472 (24.7%), **(B)** ethnicity n(%) = 6,191 (22.6%)
* Adjusted for gender, comorbidity status, deprivation, ethnicity and the restricted cubic splines of age
† ‡ Likelihood ratio test for the overall effect of deprivation (p<0.001)

For peer review only

Table 4 shows the linearly combined effect[14] between comorbidity status and deprivation on short-term mortality by DLBCL and FL. Overall, at 1 year since diagnosis, amongst FL (**Table** 4a), patients who were most deprived with multimorbidity have 3.26 (CI 2.48 – 4.28) times higher short-term mortality hazard than patients without comorbidities and least deprived. Amongst DLBCL (**Table** 4b), and for the same comparison, the short-term mortality hazard was 1.88 (CI 1.70 – 2.07) times higher at 1 year.

Table 4: Linearly combined adjusted hazard ratio of comorbidity status with deprivation level on short-term mortality (after multiple imputation) amongst **[A]** FL (deaths at 1 year: n = 1,087) and **[B]** DLBCL (1 year: n = 9,095) for patients in England from 2005-2015.

	Comorbidity status		
	**None HR* (95% CI)**	**One HR* (95% CI)**	**Multimorbidity HR* (95% CI)**
**[A] Follicular**			
**Deprivation**			
Least deprived	Ref	1.28 (1.01 – 1.61)	2.17 (1.78 – 2.64)
2	1.09 (0.90 – 1.31)	1.39 (1.03 – 1.88)	2.36 (1.80 – 3.10)
3	1.15 (0.95 – 1.39)	1.47 (1.09 – 1.98)	2.50 (1.91 – 3.26)
4	1.44 (1.19 – 1.73)	1.84 (1.36 – 2.47)	3.12 (2.40 – 4.06)
Most deprived	1.50 (1.23 – 1.84)	1.92 (1.42 – 2.59)	3.26 (2.48 – 4.28)
**[B] DLBCL**			
**Deprivation**			
Least deprived	Ref	1.24 (1.15 – 1.35)	1.44 (1.34 – 1.55)
2	1.05 (0.98 – 1.12)	1.30 (1.18 – 1.44)	1.51 (1.37 – 1.67)
3	1.13 (1.06 – 1.20)	1.40 (1.27 – 1.55)	1.63 (1.48 – 1.80)
4	1.23 (1.15 – 1.31)	1.52 (1.38 – 1.69)	1.78 (1.61 – 1.95)
Most deprived	1.30 (1.21 – 1.40)	1.62 (1.46 – 1.80)	1.88 (1.70 – 2.07)

CI: Confidence interval. **HR**: Hazard Ratio.
*Adjusted for gender, ethnicity and the restricted cubic splines of age.

For peer review only

Figure 1 shows the unadjusted (Nelson-Aalen non-parametric estimate) and standardised risks of death up to 1 year since diagnosis for FL and DLBCL by comorbidity status and deprivation. **Supplementary Tables** S4a and S4b show results of complete case and after multiple imputation. Standardised to age, gender, deprivation and ethnicity, the risk of death over the first year was consistently higher amongst those with multimorbidity compared to those with one comorbidity or none. For both FL and DLBCL, the unadjusted analysis showed that patients with multimorbidity had consistently higher cumulative incidence of death compared to those with one comorbidity or none (log rank test $p<0.001$).

In supplementary analysis we provide the crude overall survival for FL and DLBCL (**Figure 3**). From a log-rank test we found that there was strong evidence of unequal survivor functions between these subtypes ($p<0.001$). We found there was strong evidence of a difference ($p<0.001$) in survival within age groups, gender, deprivation, comorbidity status and ethnicity.

For peer review only

Discussion

We aimed to explore the association between comorbidity status, and deprivation and their combination, on short-term mortality for patients with FL or DLBCL. We found that multimorbidity and deprivation and their combined effect are strong independent predictors of short-term mortality amongst patients with DLBCL and FL in England during 2005-2015.

To our knowledge, this is the first study that investigates the association between multimorbidity, and deprivation, on short-term mortality amongst patients with DLBCL and FL in England. Despite the scarcity of research within England, our findings are consistent with previous evidence from other countries. A Swedish study found that higher comorbidity status was independently associated with a higher risk of mortality amongst patients with MCL;[29] studies in other countries also showed an association with diffuse large B-cell lymphoma.[30,31] Additionally, more deprived, compared to least deprived, patients had a higher risk of DLBCL-related mortality and there was evidence of a significant linear trend across the quintiles of deprivation. A Danish study found that higher comorbidity status was independently associated with shorter survival lengths amongst patients with any type of NHL (Hazard Ratio 1.60, CI 1.45-1.75).[32] However, these studies used a non-cancer specific comorbidity score, which underperforms (in comparison to cancer-specific scores) when using predictive models for short-term outcomes.[19] These studies suggest that the effect of comorbidity mainly occurs prior to, and shortly after, cancer diagnosis.

As the association between multimorbidity and NHL survival is not studied as widely as solid tumours, it was unclear whether there was an association between deprivation and short-term mortality for haematological malignancies. Previous studies have described a deprivation-gap in survival comparing the least- to most-deprived,[8,10,33] but have not assessed the association. To our knowledge, this is the first study to explore the association between deprivation and short-term mortality and providing evidence of a strong and independent association.

For peer review only

There are several dynamics that may explain the association observed in this study. Firstly, the presence of a comorbidity is known to affect the timely diagnosis of DLBLC and FL,[34] such that comorbidities presenting with similar symptoms to DLBLC or FL may delay the diagnosis and dissimilar symptoms may hasten the diagnosis; further research is needed to identify comorbidities that alter the timely diagnosis. Moreover, the prevalence of comorbidities increases with age, and amongst older patients with DLBCL and FL this prevalence is consistently over 60%,[35,36] which may partly explain the delay in diagnosis amongst older ages.

Secondly, guidelines of lymphoma management focus on a single-disease standard regimen, but there is little guidance on multi-disease management.[37] A systematic review found the majority of patients with a comorbidity did not receive the standard regimen and were allocated alternative, less-intense treatments.[38] Cancer care could be improved by defining clear guidelines that recommend a comorbidity-specific treatment regimen and provide an accurate definition, and a measure, of the dose-intensity.

Lastly, differences in access to treatments, or risk of adverse effects, may partly explain the multimorbidity gap in survival from DLBCL and FL; clinicians may abstain from allocating a treatment associated with a higher risk of adverse events because it can exacerbate the complex management of cancer care. Patients without comorbidities, after receiving standard treatment regimens, still experience an increased risk of cardiovascular events.[39] A first-line standard treatment for DLBCL and FL is a combination of chemotherapy and immunotherapies, such as rituximab, and is known to be effective for those of an advanced age. Rituximab is often used in combination with anthracyclines (e.g. doxorubicin), which is associated with an increase in the incidence of adverse events (e.g. cardiotoxicity) commonly in the form of congestive heart failure.[40]

The strengths of this study include the large sample size within a database of high-quality population-based clinical records with a high national coverage. We linked clinical records with the HES database, which encompasses all patients in England with a diagnosis of DLBCL and FL between 2005 and 2013. The objective data sources provide information on patients that is gathered prospectively. Furthermore, the standardised risk provides an interpretation of the risk of death that is averaged over the entire population.

For peer review only

Due to data availability, our study is limited to not including tumour stage, route to diagnosis (e.g., general practitioner referral), and treatment plan; consequently, further research is needed to dissect the effects of comorbidity, stage and treatment on survival. Since tumour stage, route and treatment allocation are considered to be on the causal pathway between comorbidity status and short-term mortality, causal inference mediation analysis is required to estimate the proportion of the effect of comorbidity status on survival that is explained by said mediators. Furthermore, as complete case analysis may lead to selection bias, we performed multiple imputation under a missing at random mechanism, which was plausible given the association between the fully observed variables and the probability of a partially observed variable being missing and the association between the partially- and fully observed variables. We obtained the same conclusions under a complete case analysis and after assuming missing at random.

In conclusion, multimorbidity and deprivation, combined and independently, are strong predictors of an increased risk of short-term mortality at 1 year since diagnosis amongst patients with DLBCL and FL in England. Therefore, public health prevention strategies are needed to reduce the short-term mortality gap due to socioeconomic inequalities and comorbidities amongst NHL patients.

For peer review only

Figure Legends

Figure 1. Risk of short-term mortality for Follicular lymphoma (n=14,043) and DLBCL (n=27,379) by comorbidity status and deprivation level in England between 2005-2015. (Solid: Aalen-Nelson approach, Dash: standardised to the empirical distribution of age, gender, and ethnicity).

Figure 2: Overlap plots for the density of predicted probabilities of comorbidity status amongst patients (n=41,422), aged 45-99, in England diagnosed with non-Hodgkin lymphoma during 2005-2013

Figure 3: Overall survival of patients (n=41,422) diagnosed with DLBCL or Follicular lymphoma, aged 45-99, in England during 2005-2013

For peer review only

Additional information

Acknowledgements: The authors wish to thank Professor Paul Lambert (Leicester University, UK) for his advice and comments on using Stata software packages in combination with multiple imputation. We would like to thank Adrian Turculet, Data Manager of the LSHTM Inequalities in Cancer Outcomes Network, for his support and assistance with the data linkage.

Author's contributions: MS, ENN and BR contributed to the conception of the study and designed the study. ENN, BR, MALF and MQ provided advice on statistical methods. MS conducted the analyses of the data and prepared the draft of the manuscript, tables and figures. ENN and BR supervised the study and provided comments on the manuscript draft. ENN, BR, MALF, MQ, SBM, ABe and ABo provided comments on the final draft of the manuscript. All authors read and approved the final manuscript.

Ethics approval and consent to participate: We obtained the statutory approvals required for this research from the Confidentiality Advisory Group (CAG) of the Health Research Authority (HRA): PIAG 1–05(c) 2007. Ethical approval was obtained from the Research Ethics Committee (REC) of the Health Research Authority (HRA): 07/MRE01/52. This work uses data provided by patients and collected by the National Health Service as part of their care and support. We used anonymised National Cancer Registry and Hospital Episode Statistics data. No consent to participate was sought from patients.

Availability of data and materials: The data that support the findings of this study are available via application to the Public Health England Office for Data Release, but restrictions apply to the availability of these data.

Funding: This research was funded by Cancer Research UK grant number C7923/A18525. The authors declare no support from any organisations for the submitted work. The design of the study, the analyses and the writing of the manuscript were solely the responsibility of the authors. The findings and conclusions in this manuscript are those of the authors and do not necessarily represent the views of Cancer Research UK.

For peer review only

Competing interests: The authors declare there are no competing interests.

For peer review only

References

1. Office for National Statistics. *Cancer registration statistics, England: 2017*. (2019).

2. Cancer Research UK. Non-Hodgkin lymphoma statistics. (2017). Available at: https://www.cancerresearchuk.org/health-professional/cancer-statistics/statistics-by-cancer-type/non-hodgkin-lymphoma. (Accessed: 24th April 2020)

3. Quaresma, M., Coleman, M. P. & Rachet, B. 40-year trends in an index of survival for all cancers combined and survival adjusted for age and sex for each cancer in England and Wales, 1971–2011: a population-based study. *Lancet* **385**, 1206–1218 (2015).

4. Shankland, K. R., Armitage, J. O. & Hancock, B. W. Non-Hodgkin lymphoma. *Lancet* **380**, 848–857 (2012).

5. Epidemiology & Cancer Statistics Group University of York. Haematological Malignancy Research Network. (2016).

6. Allemani, C. *et al.* Global surveillance of trends in cancer survival 2000–14 (CONCORD-3): analysis of individual records for 37,513,025 patients diagnosed with one of 18 cancers from 322 population-based registries in 71 countries. *Lancet* **391**, 1023–1075 (2018).

7. Thomson, C. S. & Forman, D. Cancer survival in England and the influence of early diagnosis: what can we learn from recent EUROCARE results? *Br. J. Cancer* **101**, S102–S109 (2009).

8. Rachet, B., Mitry, E., Shah, A., Cooper, N. & Coleman, M. P. Survival from non-Hodgkin lymphoma in England and Wales up to 2001. *Br. J. Cancer* **99**, S104–S106 (2008).

9. National Institute for Health and Care Excellence. *Haematological cancers: improving outcomes*. (2016).

10. Exarchakou, A., Rachet, B., Belot, A., Maringe, C. & Coleman, M. P. Impact of national cancer policies on cancer survival trends and socioeconomic inequalities in England, 1996-2013: population based study. *BMJ* **360**, k764–k764 (2018).

11. Porta, M. *A Dictionary of Epidemiology*. (Oxford University Press, 2014). doi:10.1093/acref/9780195314496.001.0001

12. Salika, T., Lyratzopoulos, G., Whitaker, K. L., Waller, J. & Renzi, C. Do comorbidities influence help-seeking for cancer alarm symptoms? A population-based survey in England. *J. Public Health*

For peer review only

(Bangkok). **40**, 340–349 (2017).

13. Fowler, H. *et al.* Comorbidity prevalence among cancer patients: a population-based cohort study of four cancers. *BMC Cancer* **20**, 2 (2020).

14. Luque-Fernandez, M. A. *et al.* Multimorbidity and short-term overall mortality among colorectal cancer patients in Spain: A population-based cohort study. *Eur. J. Cancer* **129**, 4–14 (2020).

15. International Agency for Research on Cancer. International Classification of Diseases for Oncology. (2013). Available at: http://codes.iarc.fr/. (Accessed: 4th October 2019)

16. gov.uk. National Cancer Registry and Analysis Service. (2017). Available at: https://www.gov.uk/guidance/national-cancer-registration-and-analysis-service-ncras. (Accessed: 4th October 2019)

17. Maringe, C., Fowler, H., Rachet, B. & Luque-Fernandez, M. A. Reproducibility, reliability and validity of population-based administrative health data for the assessment of cancer non-related comorbidities. *PLoS One* **12**, e0172814 (2017).

18. Charlson, M. E., Pompei, P., Ales, K. L. & MacKenzie, C. R. A new method of classifying prognostic comorbidity in longitudinal studies: Development and validation. *J. Chronic Dis.* **40**, 373–383 (1987).

19. Armitage, J. N. & van der Meulen, J. H. Identifying co-morbidity in surgical patients using administrative data with the Royal College of Surgeons Charlson Score. *Br J Surg* **97**, 772–781 (2010).

20. gov.uk. Indices of Mulitple Deprivation. (2015). Available at: https://www.gov.uk/government/statistics/english-indices-of-deprivation-2015. (Accessed: 4th October 2019)

21. National Health Service: data dictionary. Lower Super Output Area. (2018). Available at: https://www.datadictionary.nhs.uk/data_dictionary/nhs_business_definitions/l/lower_layer_super_output_area_de.asp?shownav=1. (Accessed: 4th October 2019)

22. Armitage, P., Berry, G. & Matthews, J. *Statistical methods in medical research*. (Blackwell Science, 2002).

23. Royston, P. & Lambert, P. *Flexible Parametric Survival Analysis Using Stata: Beyond the Cox Model.* (2011).

24. Robins, J. M., Hernán, M. Á. & Brumback, B. Marginal Structural Models and Causal Inference in

For peer review only

Epidemiology. *Epidemiology* **11**, (2000).

25. Cole, S. & Hernan, M. A. Adjusted survival curves with inverse probability weights. *Comput. Methods Programs Biomed.* **75**, 45–49 (2004).

26. Dupont, W. D. *Statistical Modeling for Biomedical Researchers: A Simple Introduction to the Analysis of Complex Data*. (Cambridge University Press, 2009). doi:DOI: 10.1017/CBO9780511575884

27. Armitage, J. O. Staging Non-Hodgkin Lymphoma. *CA. Cancer J. Clin.* **55**, 368–376 (2005).

28. Halpern, M. T. *et al.* Association of insurance status and ethnicity with cancer stage at diagnosis for 12 cancer sites: a retrospective analysis. *Lancet Oncol.* **9**, 222–231 (2008).

29. Little, R. J. & Rubin, D. B. *Statistical Analysis with Missing Data*. (John Wiley & Sons, Inc., 1987).

30. Glimelius, I., Smedby, K. E., Eloranta, S., Jerkeman, M. & Weibull, C. E. Comorbidities and sex differences in causes of death among mantle cell lymphoma patients – A nationwide population-based cohort study. *Br. J. Haematol.* **189**, 106–116 (2020).

31. Wästerlid, T. *et al.* Impact of comorbidity on disease characteristics, treatment intent and outcome in diffuse large B-cell lymphoma: a Swedish lymphoma register study. *J. Intern. Med.* **285**, 455–468 (2019).

32. Frederiksen, B. L., Dalton, S. O., Osler, M., Steding-Jessen, M. & de Nully Brown, P. Socioeconomic position, treatment, and survival of non-Hodgkin lymphoma in Denmark--a nationwide study. *Br J Cancer* **106**, 988–995 (2012).

33. Rachet, B. *et al.* Socioeconomic inequalities in cancer survival in England after the NHS cancer plan. *Br J Cancer* **103**, 446–453 (2010).

34. Howell, D. A. *et al.* Time-to-diagnosis and symptoms of myeloma, lymphomas and leukaemias: a report from the Haematological Malignancy Research Network. *BMC Blood Disord.* **13**, 9 (2013).

35. van Spronsen, D. J., Janssen-Heijnen, M. L., Breed, W. P. & Coebergh, J. W. Prevalence of co-morbidity and its relationship to treatment among unselected patients with Hodgkin's disease and non-Hodgkin's lymphoma, 1993-1996. *Ann Hematol* **78**, 315–319 (1999).

36. Janssen-Heijnen, M. L. *et al.* A population-based study of severity of comorbidity among patients with non-Hodgkin's lymphoma: prognostic impact independent of International Prognostic Index. *Br J Haematol* **129**, 597–606 (2005).

For peer review only

37. National Institute for Health and Care Excellence. *Non-Hodgkin's lymphoma: diagnosis and management*. (National Institute for Health and Care Excellence, 2016).

38. Terret, C., Albrand, G., Rainfray, M. & Soubeyran, P. Impact of comorbidities on the treatment of non-Hodgkin's lymphoma: a systematic review. *Expert Rev. Hematol.* **8**, 329–341 (2015).

39. Linschoten, M. *et al.* Cardiovascular adverse events in patients with non-Hodgkin lymphoma treated with first-line cyclophosphamide, doxorubicin, vincristine, and prednisone (CHOP) or CHOP with rituximab (R-CHOP): a systematic review and meta-analysis. *Lancet Haematol.* **7**, e295–e308 (2020).

40. McGowan, J. V *et al.* Anthracycline Chemotherapy and Cardiotoxicity. *Cardiovasc. Drugs Ther.* **31**, 63–75 (2017).

For peer review only

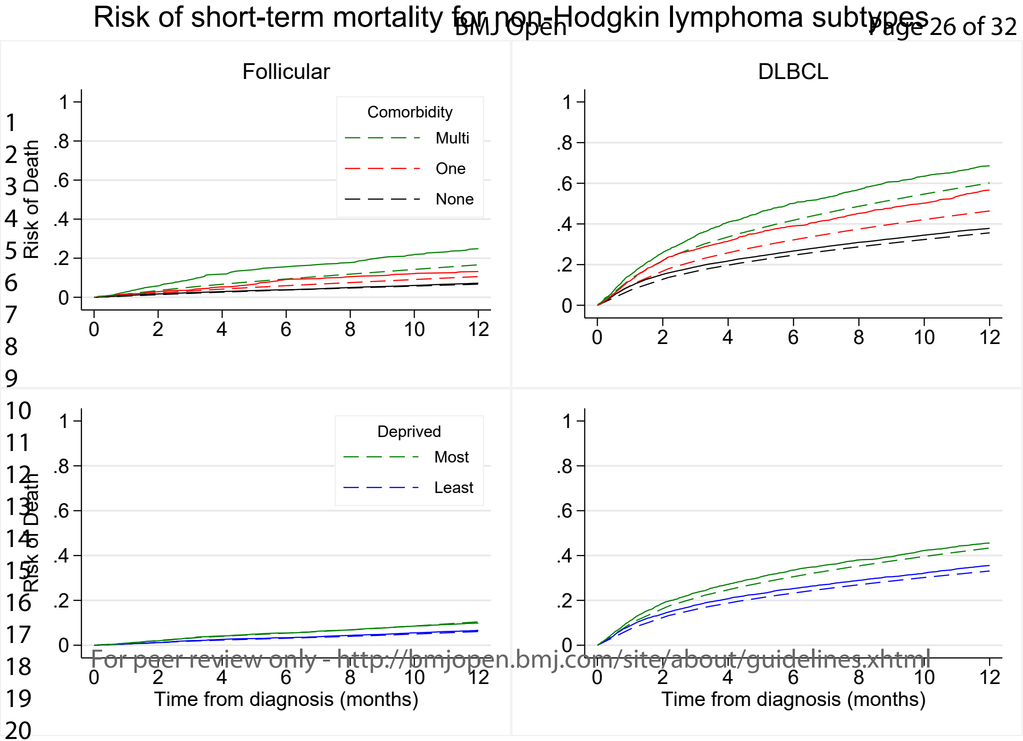

For peer review only - http://bmjopen.bmj.com/site/about/guidelines.xhtml

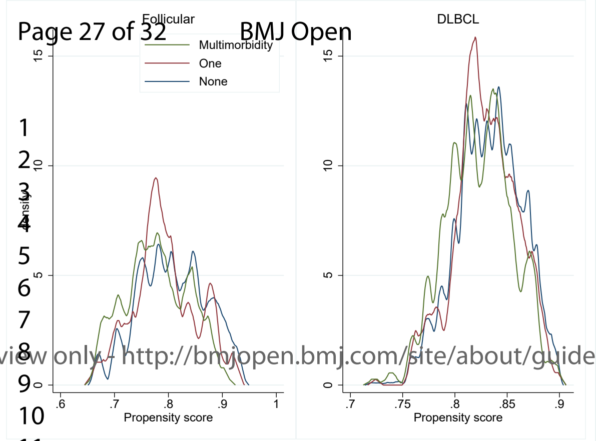

view only - http://bmjopen.bmj.com/site/about/guide

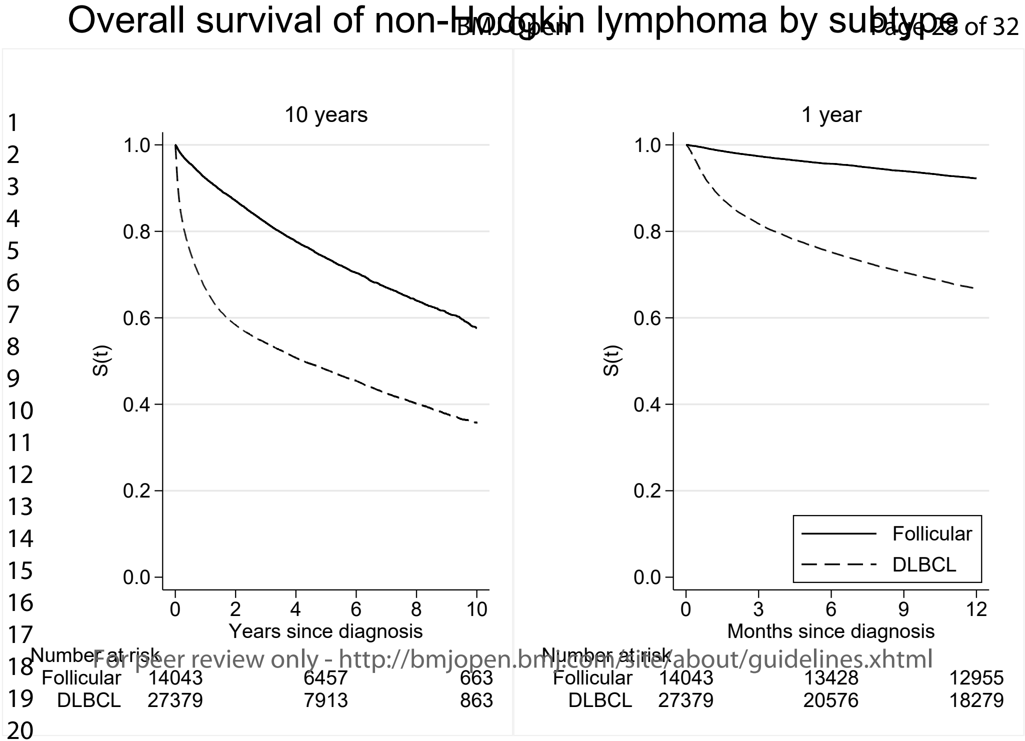

For peer review only - http://bmjopen.bmj.com/site/about/guidelines.xhtml

Supplementary Tables

This page is intentionally blank. Please move to next page.

For peer review only

Supplementary Table S1. Distribution of non-Hodgkin lymphoma subtypes for patients in England diagnosed from 2005-2013, with respective morphology and topography ICD-O-3 codes.

Index	Site group (subtype)	Progression	Topography	Morphology	n	%
1	CLL/SLL*	Indolent	C82.0-C85.9	9670, 9823	3,875	5.08
2	Waldenstrom macroglobulinemia	Indolent	C82.0-C85.9	9761	2,398	3.14
3	Mantle cell	Indolent	C82.0-C85.9	9673	3,458	4.53
4	Diffuse large B-cell	Aggressive	C82.0-C85.9	9680, 9688, 9737-9738	27,379	35.89
5	Burkitt	Aggressive	C82.0-C85.9	9687, 9826	695	0.91
6	Follicular	Indolent	C82.0-C85.9	9690-9691, 9695, 9698	14,043	18.41
7	Mature T-cell	Aggressive	C82.0-C85.9	9702	5,127	6.72
8	Marginal zone B-cell	Indolent	C82.0-C85.9	9689, 9699, 9760, 9764, 9699	4,277	5.61
**Subtotal**					**61,252**	**80.30**
9	Not Otherwise Specified	n/a	C82.0-C85.9	9591, 9675, 9735	9,581	12.56
10	Other***	n/a	C82.0-C85.9	9591, 9675, 9735	5,449	7.14
**Total**					**76,282**	**100.00****

n/a – not applicable; there was no subtype information
* Chronic lymphocytic leukaemia/Small-cell lymphocytic lymphoma
** Percentages may not equate to 100.0% due to rounding
*** The morphology code specifies these patients are diagnosed with NHL. However, the description states ‘other’; these patients are classified similarly to ‘Not Otherwise Specified’.

For peer review only - http://bmjopen.bmj.com/site/about/guidelines.xhtml

Supplementary Table S2: Comorbidities and their diagnostic ICD-10 codes

Comorbidity	ICD-10
Myocardial infarction	I21.x, I22.x, I25.2
Congestive heart failure	I11.0, I13.0, I13.2, I25.5, I42.0, I42.5–I42.9, I43.x, I50.x, P29.0
Peripheral vascular disease	I70.x, I71.x, I73.1, I73.8, I73.9, I77.1, I79.0, I79.2, K55.1, K55.8, K55.9, Z95.8, Z95.9
Cerebrovascular disease	G45.x, G46.x, H34.0, I60.x–I69.x
Dementia	F00.x–F03.x, F05.1, G30.x, G31.1
Chronic obstructive pulmonary disease	I27.9, J40.x–J47.x, J60.x–J67.x, J68.4, J70.1, J70.3
Rheumatic disease	M05.x, M06.x, M31.5, M32.x–M34.x, M35.1, M35.3, M36.0
Liver disease	B18.x, K70.0–K70.3, K70.9, K71.3–K71.5, K71.7, K73.x, K74.x, K76.0, K76.2–K76.4, K76.8, K76.9, Z94.4, K71.1, K72.1, K72.9, K76.5, K76.6, K76.7, I85.0, I85.9, I86.4, I98.2, K70.4,
Diabetes without chronic complication	E10.0, E10.1, E10.6, E10.8, E10.9, E11.0, E11.1, E11.6, E11.8, E11.9, E12.0, E12.1, E12.6, E12.8, E12.9, E13.0, E13.1, E13.6, E13.8, E13.9, E14.0, E14.1, E14.6, E14.8, E14.9
Diabetes with chronic complication	E10.7, E11.2–E11.5, E11.7, E12.2–E12.5, E12.7, E13.2–E13.5, E13.7, E14.2–E14.5, E14.7
Hemiplegia or paraplegia	G04.1, G11.4, G80.1, G80.2, G81.x, G82.x, G83.0–G83.4, G83.9
Renal disease	I12.0, I13.1, N03.2–N03.7, N05.2–N05.7, N18.x, N19.x, N25.0, Z49.0–Z49.2, Z94.0, Z99.2
AIDS/HIV	B20.x–B22.x, B24.x

ICD-10: International Classification of Diseases, 10th Revision
Diabetes with/without chronic complication is combined in the RCS Charlson Comorbidity Score

For peer review only

Supplementary Table S3. Sociodemographic characteristics, route of diagnosis, comorbidity status and Ann Arbor cancer stage distribution, of Follicular (n=14,043) and DLBCL (n=27,379) lymphomas in England, 2005-2013.

	Follicular N = 14,043	DLBCL N = 27,739	OR* (95% CI)	p-value
**Age** *(mean; SD)***	66.7 (11.0)	70.8 (11.3)	1.37 (1.35 – 1.40)	<0.001
**Gender**				
Male	6,512 (46.4)	14,652 (53.5)	Ref	Ref
Female	7,531 (53.6)	12,727 (46.5)	0.75 (0.72 - 0.78)	<0.001
**Deprivation**				
Least deprived	3,284 (23.4)	5,895 (21.5)	Ref	Ref
2	3,228 (23.0)	6,238 (22.8)	1.08 (1.01 – 1.14)	0.016
3	3,013 (21.5)	5,756 (21.0)	1.06 (1.00 – 1.13)	0.047
4	2,609 (18.6)	5,341 (19.5)	1.14 (1.07 – 1.22)	<0.001
Most deprived	1,909 (13.6)	4,149 (15.2)	1.21 (1.13 – 1.30)	<0.001
**Comorbidity status**				
Non	12,898 (91.9)	24,269 (88.6)	Ref	Ref
One	623 (4.4)	1,538 (5.6)	1.60 (1.45 – 1.77)	<0.001
Multimorbidity	522 (3.7)	1,572 (5.7)	1.58 (1.43 – 1.75)	<0.001
**Route**				
Elective	11,211 (86.7)	17,280 (66.3)	Ref	Ref
Emergency	1,727 (13.4)	8,799 (33.7)	3.31 (3.12 – 3.50)	<0.001
Missing	*1,105 (7.9)*	*1,300 (4.8)*	-	-
**Stage**				
I	848 (26.6)	1.680 (27.8)	Ref	Ref
II	473 (14.8)	1.169 (19.3)	1.25 (1.09 – 1.43)	0.001
III	822 (25.7)	1.046 (17.3)	0.64 (0.57 – 0.73)	<0.001
IV	1050 (32.9)	2.152 (35.6)	1.03 (0.93 – 1.16)	0.548
Missing	*10,850 (77.3)*	*21,332 (77.9)*	-	-
**Ethnicity**				
White	10.110 (95.6)	20.204 (95.4)	Ref	Ref
Other	461 (4.4)	984 (4.6)	1.07 (0.95 – 1.20)	0.254
Missing	*3,472 (24.7)*	*6,191 (22.6)*	-	-

* Odds ratio from a complete case analysis comparing the odds of DLBCL to Follicular lymphoma
** 10-year increase in age

For peer review only

Supplementary Table S4a: Risk of short-term mortality amongst patients diagnosed with Follicular lymphomas (n=14,043) by comorbidity status in England between 2005 and 2013

	**Complete Case Analysis**						**After Multiple Imputation**					
	**Comorbidity status**						**Comorbidity status**					
	**None**		**One**		**Multimorbidity**		**None**		**One**		**Multimorbidity**	
**Month**	*CH*	*95% CI*	*CH*	*95% CI*	*CH*	*95% CI*	*CH*	*95% CI*	*CH*	*95% CI*	*CH*	*95% CI*
1	0.6	0.4 – 0.9	1.0	0.7 - 1.4	1.6	1.0 – 2.3	0.8	0.7 – 1.0	1.3	1.1 – 1.5	2.4	1.6 – 3.2
2	1.4	1.0 – 1.7	2.2	1.6 - 2.7	3.5	2.3 – 4.6	1.7	1.5 – 1.9	2.6	2.2 – 3.0	4.8	3.4 – 6.3
3	2.0	1.6 – 2.4	3.2	2.6 - 3.9	5.2	3.6 – 6.7	2.4	2.2 – 2.7	3.7	3.2 – 4.2	6.8	4.9 – 8.7
4	2.6	2.2 – 3.1	4.2	3.5 - 4.9	6.7	4.8 – 8.6	3.0	2.7 – 3.3	4.6	4.0 – 5.2	8.4	6.1 – 10.8
5	3.2	2.6 – 3.7	5.1	4.3 - 5.9	8.1	5.9 – 10.3	3.6	3.3 – 3.9	5.4	4.8 – 6.1	9.9	7.2 – 12.6
6	3.7	3.1 – 4.3	5.9	5.0 - 6.9	9.4	6.9 – 11.9	4.1	3.8 – 4.4	6.2	5.5 – 7.0	11.3	8.3 – 14.3
7	4.2	3.6 – 4.9	6.7	5.7 - 7.7	10.6	7.8 – 13.4	4.6	4.2 – 5.0	7.0	6.2 – 7.9	12.7	9.3 – 16.0
8	4.7	4.0 – 5.5	7.5	6.4 - 8.6	11.8	8.8 – 14.9	5.1	4.8 – 5.5	7.8	6.9 – 8.8	14.1	10.4 – 17.8
9	5.2	4.5 – 6.0	8.3	7.2 - 9.4	13.1	9.7 – 16.4	5.7	5.3 – 6.1	8.7	7.6 – 9.7	15.5	11.5 – 19.5
10	5.7	5.0 – 6.5	9.1	7.9 - 10.3	14.3	10.6 – 17.9	6.2	5.8 – 6.7	9.5	8.4 – 10.6	17.0	12.6 – 21.3
11	6.2	5.4 – 7.0	9.9	8.6 - 11.1	15.4	11.5 – 19.4	6.8	6.4 – 7.3	10.3	9.2 – 11.5	18.4	13.7 – 23.1
12	6.7	5.9 – 7.5	10.6	9.2 - 12.0	16.6	12.3 – 20.9	7.4	6.9 – 7.8	11.2	9.9 – 12.4	19.8	14.8 – 24.9

CH – cumulative hazard of death (e x 10^2)
95% CI – confidence interval (e x 10^2)

Supplementary Table S4b: Risk of short-term mortality amongst patients diagnosed with DLBCL (n=27,379) by comorbidity status in England between 2005 and 2013

	**Complete Case Analysis**						**After Multiple Imputation**					
	**Comorbidity status**						**Comorbidity status**					
	**None**		**One**		**Multimorbidity**		**None**		**One**		**Multimorbidity**	
**Month**	*CH*	*95% CI*	*CH*	*95% CI*	*CH*	*95% CI*	*CH*	*95% CI*	*CH*	*95% CI*	*CH*	*95% CI*
1	7.4	6.9 – 7.9	9.7	9.0 – 10.4	13.2	11.5 – 14.9	9.2	8.9 – 9.6	12.0	11.4 – 12.5	16.1	14.5 – 17.7
2	12.9	12.2 – 13.7	17.0	16.0 – 18.0	23.0	20.4 – 25.7	15.3	14.8 – 15.7	19.7	18.8 – 20.5	26.3	23.9 – 28.8
3	16.8	16.0 – 17.6	22.0	20.8 – 23.2	30.0	26.6 – 33.3	19.1	18.6 – 19.7	24.6	23.6 – 25.6	32.8	29.8 – 35.8
4	19.8	18.9 – 20.7	25.9	24.5 – 27.2	35.4	31.5 – 39.3	22.1	21.6 – 22.7	28.4	27.2 – 29.5	37.7	34.3 – 41.1
5	22.4	21.4 – 23.3	29.2	27.7 – 30.7	39.9	35.6 – 44.3	24.8	24.1 – 25.4	31.7	30.4 – 32.9	42.0	38.2 – 45.8
6	24.7	23.7 – 25.7	32.2	30.6 – 33.8	43.9	39.2 – 48.7	27.1	26.5 – 27.8	34.7	33.3 – 36.0	45.9	41.8 – 50.0
7	26.8	25.8 – 27.8	34.9	33.3 – 36.6	47.5	42.4 – 52.6	29.3	28.6 – 30.0	37.5	36.0 – 38.9	49.6	45.2 – 53.9
8	28.8	27.7 – 29.8	37.5	35.8 – 39.2	50.8	45.4 – 56.2	31.4	30.7 – 32.1	40.1	38.5 – 41.6	53.0	48.3 – 57.6
9	30.6	29.5 – 31.7	39.9	38.1 – 41.7	53.8	48.2 – 59.5	33.3	32.6 – 34.0	42.5	40.9 – 44.1	56.2	51.2 – 61.1
10	32.4	31.2 – 33.5	42.2	40.3 – 44.0	56.7	50.8 – 62.6	35.1	34.4 – 35.9	44.8	43.2 – 46.5	59.2	54.0 – 64.4
11	34.0	32.8 – 35.2	44.3	42.4 – 46.3	59.4	53.3 – 65.5	36.9	36.1 – 37.7	47.1	45.3 – 48.8	62.1	56.7 – 67.6
12	35.6	34.4 – 36.9	46.4	44.3 – 48.5	62.0	55.6 – 68.4	38.6	37.7 – 39.4	49.2	47.4 – 51.0	64.9	59.2 – 70.6

CH – cumulative hazard (e x 10^2)
95% CI – confidence interval (e x 10^2)

For peer review only

For peer review only - http://bmjopen.bmj.com/site/about/guidelines.xhtml

BMJ Open

Association between multimorbidity and socioeconomic deprivation on short-term mortality amongst patients with Diffuse Large B-cells or Follicular lymphomas in England: a nationwide cohort study

Journal:	*BMJ Open*
Manuscript ID	bmjopen-2021-049087.R1
Article Type:	Original research
Date Submitted by the Author:	09-Aug-2021
Complete List of Authors:	Smith, Matthew; London School of Hygiene & Tropical Medicine, Non-Communicable Disease Epidemiology<br>Njagi, Edmund Njeru; London School of Hygiene & Tropical Medicine<br>Belot, Aurelien; London School of Hygiene & Tropical Medicine<br>Leyrat, Clémence; London School of Hygiene & Tropical Medicine, Department of Non-Communicable Disease Epidemiology<br>Bonaventure, Audrey; University of Paris, Epidemiology of Childhood and Adolescent Cancers Team<br>Benitez Majano, Sara ; London School of Hygiene & Tropical Medicine, Department of Non-Communicable Disease Epidemiology<br>Rachet, Bernard; London School of Hygiene & Tropical Medicine, Non-Communicable Disease Epidemiology<br>Luque Fernandez, Miguel Angel; London School of Hygiene and Tropical Medicine, Epidemiology and Population Health; Instituto de Investigación Biosanitaria de Granada, Noncommunicable Disease and Cancer Epidemiology Group
**Primary Subject Heading**:	Epidemiology
Secondary Subject Heading:	Public health, Epidemiology, Global health, Oncology, Research methods
Keywords:	Epidemiology < ONCOLOGY, STATISTICS & RESEARCH METHODS, Lymphoma < HAEMATOLOGY

SCHOLARONE™
Manuscripts

I, the Submitting Author has the right to grant and does grant on behalf of all authors of the Work (as defined in the below author licence), an exclusive licence and/or a non-exclusive licence for contributions from authors who are: i) UK Crown employees; ii) where BMJ has agreed a CC-BY licence shall apply, and/or iii) in accordance with the terms applicable for US Federal Government officers or employees acting as part of their official duties; on a worldwide, perpetual, irrevocable, royalty-free basis to BMJ Publishing Group Ltd ("BMJ") its licensees and where the relevant Journal is co-owned by BMJ to the co-owners of the Journal, to publish the Work in this journal and any other BMJ products and to exploit all rights, as set out in our licence.

The Submitting Author accepts and understands that any supply made under these terms is made by BMJ to the Submitting Author unless you are acting as an employee on behalf of your employer or a postgraduate student of an affiliated institution which is paying any applicable article publishing charge ("APC") for Open Access articles. Where the Submitting Author wishes to make the Work available on an Open Access basis (and intends to pay the relevant APC), the terms of reuse of such Open Access shall be governed by a Creative Commons licence – details of these licences and which Creative Commons licence will apply to this Work are set out in our licence referred to above.

Other than as permitted in any relevant BMJ Author's Self Archiving Policies, I confirm this Work has not been accepted for publication elsewhere, is not being considered for publication elsewhere and does not duplicate material already published. I confirm all authors consent to publication of this Work and authorise the granting of this licence.

For peer review only

Title

Association between multimorbidity and socioeconomic deprivation on short-term mortality amongst patients with Diffuse Large B-cells or Follicular lymphomas in England: a nationwide cohort study

Authors

Matthew J. Smith[1*], Edmund Njeru Njagi[1], Aurélien Belot[1], Clémence Leyrat[1,2], Audrey Bonaventure[3], Sara Benitez Majano[1], Bernard Rachet[1], Miguel Angel Luque Fernandez[1,4,5]

Authors' affiliations

[1] Inequalities in Cancer Outcomes Network, Department of Non-Communicable Disease Epidemiology, London School of Hygiene and Tropical Medicine, Keppel Street, London, WC1E 7HT, UK

[2] Department of Medical Statistics, London School of Hygiene and Tropical Medicine, Keppel Street, London, WC1E 7HT, UK

[3] CRESS, Université de Paris, INSERM, UMR 1153, Epidemiology of Childhood and Adolescent Cancers Team, Villejuif, France

[4] Noncommunicable Disease and Cancer Epidemiology Group, Instituto de Investigación Biosanitaria de Granada, Ibs.GRANADA, Andalusian School of Public Health, Granada, Spain

[5] Centro de Investigación Biomédica en Red de Epidemiología y Salud Pública (CIBER of Epidemiology and Public Health, CIBERESP), Madrid, Spain

Corresponding author*

Matthew J. Smith
Keppel Street, London, WC1E 7HT, UK
ORCID: 0000-0002-8502-0056
Email: *matthew.smith1@lshtm.ac.uk*

Word count: Abstract: 193; Text: 2807; Tables: 4; Figures: 2

For peer review only

Abstract

Objectives: We aimed to assess the association between multimorbidity and deprivation on short-term mortality amongst DLBCL and FL patients in England.

Setting: The association of multimorbidity and socioeconomic deprivation on survival among patients diagnosed with Diffuse Large B-cell (DLBCL) and Follicular lymphoma (FL) in England between 2005 and 2013. We linked the English population-based cancer registry with electronic health records databases and estimated adjusted mortality rate ratios by multimorbidity and deprivation status. Using flexible hazard-based regression models, we computed DLBCL and FL standardised mortality risk by deprivation and multimorbidity at 1 year.

Results: Overall, 41,422 patients aged 45-99 years were diagnosed with DLBCL or FL in England during 2005-2015. Most deprived FL patients with multimorbidities had three times higher hazard of 1 year mortality (HR: 3.3, CI: 2.48 – 4.28, $p<0.001$) than least deprived patients without comorbidity; amongst DLBCL there was approximately twice the hazard (HR: 1.9, CI: 1.70– 2.07, $p<0.001$).

Conclusions: Multimorbidity, deprivation, and their combination, are strong and independent predictors of an increased short-term mortality risk amongst DLBCL and FL patients in England. Public health measures targeting the reduction of multimorbidity amongst most deprived DLBCL and FL patients are needed to reduce the short-term mortality gap.

Key words: Cancer epidemiology, Diffuse Large B-cell lymphoma, Follicular lymphoma, multimorbidity, deprivation, survival analysis

For peer review only

Strengths and limitations of this study

- Data contains a large sample size of high-quality population-based clinical records with a high national coverage of information on all patients diagnosed with Diffuse Large B-cell or Follicular lymphomas in England during 2005 to 2013.
- Population based administrative hospital discharge data was used for the assessment of comorbid conditions, and selection bias was reduced by restricting records of comorbidities to occurring between 6- and 24-months prior to the date of cancer diagnosis.
- 1-year cumulative mortality hazard was modelled using a flexible parametric modelling approach and included restricted cubic splines to account for non-linear effects of continuous variables.
- Modern methods (i.e., standardisation) were used to control for confounding of patient baseline characteristics; information on lifestyle characteristics was unavailable.
- As there was missing data we performed a sensitivity analysis with multiple imputation using chained equations, we found consistency of our conclusions under different missing data assumptions.

For peer review only

Introduction

In England, non-Hodgkin lymphoma (NHL) is the sixth most commonly diagnosed cancer in England with an incidence rate of 23.2 per 100,000 people.[1] Apart from lung cancer, survival estimates of NHL (79.4% survival probability at 1 year) are amongst the lowest of the six most common cancers.[2,3] NHL encompasses a heterogeneous group of malignancies with diverse histological patterns; in addition, the commonest NHL subtypes are diffuse large B-cell lymphoma (DLBCL) and follicular lymphoma (FL),[4] patients of which show a large variation in survival.[5] Cancer survival in England is lower than other European countries,[6] but is similar when restricting to those surviving after 1 year:[7] identifying, and influencing, the factors of short-term mortality could reduce the gap in survival.

Over the past decades, patient survival of FL has steadily improved and stagnated for DLBCL;[3] furthermore, disparities in survival between deprivation levels remains.[8] Public health policies have increased awareness and set targets, such as minimising the length of time from referral to treatment, to reduce the inequalities;[9] however, since their implementation, there has been no evidence that the National Health Service (NHS) Cancer Plan had an impact on the inequalities in cancer survival.[10] The deprivation-gap in survival is still apparent, despite the NHS Cancer Plan and successive policies.[9]

Comorbidities, which refers to the presence of a long-term health condition additional, but unrelated, to the underlying cancer,[11] tends to mask cancer symptoms, delaying cancer diagnosis and decreases survival.[12] Older age groups and those living in more deprived areas experience more comorbidities. With a global aging population, the prevalence of comorbidities is expected to increase.[13] The association between multimorbidity and survival is described for other cancers,[14] but for DLBCL and FL this relationship remains unclear.

We aim to identify the association between multimorbidity and risk of short-term mortality amongst DLBCL and FL patients in England. We hypothesise that multimorbidity and deprivation, independently and combined, contribute to an increased risk of death after DLBCL or FL diagnosis.

For peer review only - http://bmjopen.bmj.com/site/about/guidelines.xhtml

Methods

Study Design, participants, data and setting

We used the data from a retrospective population-based cohort study with DLBCL and FL patients diagnosed between 1st January 2005 and 31st December 2013 and followed up to 31st December 2015. DLBCL and FL diagnoses were made according to the International Classification of Diseases for Oncology, 3rd edition, based on codes C82.0-C85.9[15] (**Supplementary Table S1** shows the subtype categorisation). Patients entered the study on the date of their diagnosis and were followed up until death or censored at 1 year, whichever occurred first.

Data was obtained from population-based cancer registries within the English National Cancer Registry and Analysis Service (CAS)[16] and linked to patient's electronic health records from Hospital Episode Statistics (HES). CAS contains patient and tumour variables including relevant dates (birth, diagnosis, and vital status), sex, age at diagnosis, deprivation, cancer site and morphology. We used population based administrative hospital discharge data for the assessment of comorbid conditions; we analysed HES data (containing comorbid conditions records) according to the International Classification of Diseases, 10th revision (**Supplementary Table S2**), for the period 2003 to 2015. HES contains clinical, administrative, and demographic information about individual patients. To avoid selection bias including cancer related comorbidities, we restricted retrospective records of comorbidities to occurring between 6- and 24-months prior to the date of DLBCL and FL diagnosis.[17]

Patient and public involvement

No patient or public involvement.

For peer review only - http://bmjopen.bmj.com/site/about/guidelines.xhtml

Outcome, exposure and other variables

The outcome of this study was the time since diagnosis up to death observed within the first year after diagnosis of DLBLC and FL patients (patients alive were censored at survival time defined with the date of last known vital status), the main exposures were multimorbidity status and deprivation. Due to data availability and clinical reasoning, we include as confounders age, sex, and ethnicity. Due to the positivity assumption, and the chances of having a multimorbidity, we included patients aged above 45 years at diagnosis (**Figure 1**). The positivity assumption states that there is a nonzero probability of receiving any level of comorbidity status for every combination of values of the independent variables among the patients in the population.[18]

Comorbidity status was classified according to the Royal College of Surgeons (RCS) Charlson score (an adaptation of the Charlson comorbidity index[19]) that includes 12 categories for comorbidities, excludes a category (peptic ulcer disease) and groups diseases together (e.g. diabetes mellitus codes with or without complications were grouped into a single category). The score was categorised into those with none, one comorbidity (whatever the type), or two or more comorbidities (defined as *multimorbidity*). The score does not weight the comorbidities assuming that any comorbidity has the same impact on short-term mortality.[20]

Area-level deprivation was categorised into one of five quintiles (5th is most deprived). Deprivation was used as a proxy of individual level socioeconomic status. We used the Index of Multiple Deprivation[21] (IMD), which is an area-level deprivation score based on the Lower Super Output Area[22] (*LSOA*) residence of the patient at the time of cancer diagnosis. LSOA is a geographical location with a median of 1500 inhabitants.

Ethnicity due to data sparsity amongst ethnic minorities was recorded as either white or other. Route to diagnosis (CAS dataset), although not considered in our analysis because it was on the causal pathway, is included in the imputation models for missing data.

Statistical Analysis

We described the characteristics of DLBLC and FL patients using counts and proportions, and calculated odds ratios of having a lymphoma type along with Wald test p-values (**Supplementary Table S3**). We assessed the

For peer review only - http://bmjopen.bmj.com/site/about/guidelines.xhtml

unadjusted association between having multimorbidity and patients' characteristics, using chi-square tests. Then, we compute the number of deaths, person-time at risk, and unadjusted rates of deaths per 100 person-years and rate ratios with 95% confidence intervals (CI) by patients' characteristics by DLBCL and FL subtypes.

The follow-up time for those who died is from the date of diagnosis until death, for those alive it was until administrative censoring at 12 months (no lost to follow-up before 12 months was observed). To describe the multimorbidity and deprivation short-term mortality risk amongst DLBCL and FL patients, we computed the one year cumulative hazard obtained using the non-parametric Nelson-Aalen estimator.[23] Then, we computed adjusted short-term mortality risk by patient characteristics using a flexible parametric modelling approach to model the non-linear change in mortality risk over one year. We included restricted-cubic spline to model the baseline hazard,[24] with three knots located at the 25th, 50th, and 75th percentiles of the log event times. To define the model, let t be the time since DLBCL or FL diagnosis until death or censoring. We define the log cumulative motality hazard as

$$\ln\left[H\left(t \,\middle|\, \boldsymbol{x}_i, A_i, Dep_i\right)\right] = \gamma_0 + \gamma_1 z_{1i} + \gamma_2 z_{2i} + \gamma_3 A_{1i} + \gamma_4 A_{2i} + \sum_{k=2}^{5} \beta_{0k} \cdot Dep_{ik} + \sum_{m=1}^{M} \boldsymbol{\beta}_m x_{im}$$

where z_i are the indicators for the three knots of the baseline restricted cubic splines. The model specification included the restricted cubic splines for the continuous variable age are given by A_i where the knots are placed at 1 and 6 months, deprivation, and the vector x_{im} of categorical covariates (i.e., multimorbidity, ethnicity and sex). Restricted cubic splines were included to minimise residual confounding and to account for the non-linear association between age and the cumulative hazard. From the model, we derived the cumulative incidence of death at 1 year by comorbidity status (i.e. none, one, or multimorbidity) standardized to the empirical distribution of age, sex, ethnicity and deprivation.[25,26]

We used the same modelling approach to evaluate the linearly combined effect of multimorbidity and derivation on short-term mortality, assuming the effect of multimorbidity is constant across levels of deprivation.[27] We assume that both effects (multimorbidity and deprivation) are independently associated with the mortality rate and that their effect is constant and the rate is increasing linearly.

For peer review only - http://bmjopen.bmj.com/site/about/guidelines.xhtml

In sensitivity analysis, we evaluated the robustness of our results to the missing ethnicity records by utilising multiple imputation using chained equations (the fully conditional specification multiple imputation approach), under a missing at random assumption. We included route of cancer diagnosis and Ann Arbor[28] cancer stage as partially observed auxiliary variables because these variables were predictive of the probability of missing values (established via exploratory analysis of the missing data indicators) and are predictive of the underlying values themselves (established from clinical and epidemiological reasoning).[29] We generated 20 imputed datasets. The imputation model for the partially observed variable (ethnicity) was defined as a logistic regression model including all explanatory variables in the substantive model, the vital status and Nelson-Aalen estimate of the cumulative hazard, and the auxiliary variables (route to, and stage at, diagnosis). We then fit the substantive model to each of the 20 imputed datasets and from these estimates we predicted the standardised survival (using *stpm2* package), derived the cumulative hazard (-log[Survival probability]) for each imputed dataset and combined the survival probability estimates using Rubin's rules.[30]

Analyses were performed in Stata version 16 (StataCorp, College Station, Texas, U.S.); the *stpm2* package was used to estimate flexible parametric survival models, and the *standsurv* command to compute standardized mortality risks. The *mi impute* command was used for multiple imputation and the *mi estimate* command to combine estimates.

Results

Overall, 41,422 patients in England, aged from 45 to 99 years, were diagnosed with either DLBCL or FL between 2005 and 2013 in England. Of 14,043 patients with FL, amongst those who died within 1-year, the proportion of patients with multimorbidity compared to no comorbidity was 3.2 times higher (22.0% vs. 6.9%, respectively) (**Table 1**); of 27,379 patients diagnosed with DLBCL, this comparison was 1.6 times higher (49.7% vs. 31.5%, respectively). For both DLBCL and FL subtypes, the proportion of multimorbidity was higher amongst those living in more deprived areas. In DLBCL only, there was a higher proportion of having any comorbidity amongst males compared to females (p<0.001); for FL only, this proportion was slightly higher amongst females. As expected, multimorbidity was more prevalent amongst older age groups.

For peer review only - http://bmjopen.bmj.com/site/about/guidelines.xhtml

For peer review only

For peer review only

For peer review only - http://bmjopen.bmj.com/site/about/guidelines.xhtml

Table 1: Vital status, age, sex, deprivation level and ethnicity according to the comorbidity status amongst n = 41,422 patients with non-Hodgkin lymphoma in England between 2005 and 2013 (27,379 DLBCL cases and 14,043 FL cases).

	No comorbidity N (%)	Comorbidity N(%)	Multimorbidity N(%)	p-value *
**Diffuse Large B-cell lymphoma** (n = 27,379)				
**Vital status at 1 year**				
Alive	16,621 (68.5)	872 (56.7)	791 (50.3)	<0.001
Dead	7,648 (31.5)	666 (43.3)	781 (49.7)	
**Sex**				
Male	12,904 (53.2)	794 (51.6)	954 (60.7)	<0.001
Female	11,365 (46.8)	744 (48.4)	618 (39.3)	
**Age at diagnosis (y)**				
45-54	2,685 (11.1)	80 (5.2)	96 (6.1)	<0.001
55-64	5,154 (21.2)	257 (16.7)	200 (12.7)	
65-74	7,337 (30.2)	432 (28.1)	439 (27.9)	
75+	9,093 (37.5)	769 (50.0)	837 (53.2)	
**Deprivation**				
Least deprived	5,348 (22.0)	291 (18.9)	256 (16.3)	<0.001
2	5,586 (23.0)	318 (20.7)	334 (21.3)	
3	5,115 (21.1)	324 (21.1)	317 (20.2)	
4	4,665 (19.2)	337 (21.9)	339 (21.6)	
Most deprived	3,555 (14.7)	268 (17.4)	326 (20.7)	
**Ethnicity**				
White	17,831 (95.5)	1,204 (96.6)	1,169 (92.6)	<0.001
Other	848 (4.5)	43 (3.5)	93 (7.4)	
**Follicular lymphoma** (n = 14,043)				
**Vital status at 1 year**				
Alive	12,003 (93.1)	546 (87.6)	407 (78.0)	<0.001
Dead	895 (6.9)	77 (12.4)	115 (22.0)	
**Sex**				
Male	5,980 (46.4)	275 (44.1)	257 (49.2)	0.227
Female	6,918 (53.6)	348 (55.9)	265 (50.8)	
**Age at diagnosis (y)**				
45-54	2,246 (17.4)	42 (6.7)	33 (6.3)	<0.001
55-64	3,769 (29.2)	140 (22.5)	82 (15.7)	
65-74	3,952 (30.6)	199 (31.9)	159 (30.5)	
75+	2,931 (22.7)	242 (38.8)	248 (47.5)	
**Deprivation**				
Least deprived	3,091 (24.0)	113 (18.1)	80 (15.3)	<0.001
2	3,025 (23.5)	122 (19.6)	81 (15.5)	
3	2,759 (21.4)	136 (21.8)	118 (22.6)	
4	2,356 (18.3)	123 (19.7)	130 (24.9)	
Most deprived	1,667 (12.9)	129 (20.7)	113 (21.7)	
**Ethnicity**				
White	9,226 (95.7)	498 (96.3)	386 (92.8)	0.012
Other	412 (4.3)	19 (3.7)	30 (7.2)	

Missing values: Ethnicity n(%): DLBCL = 6,191 (22.6%), FL = 3,472 (24.7%)
* Chi-squared test of association between the baseline characteristic and comorbidity status

For peer review only

Tables 2a and 2b show the person-time at risk and unadjusted mortality rate of death for DLBLC and FL at one year after diagnosis. Amongst FL and DLBCL patients, 1087 (7.7%) and 9,095 (33.2%) died before 1 year, respectively (**Table** 2a and 2b**)**. Amongst FL, those with one comorbidity or multimorbidity had 1.9 (95% CI: 1.46 – 2.33, p<0.001) or 3.5 (95% CI: 2.90 – 4.27, p<0.001) times the mortality rate, respectively, compared to those with no comorbidity (**Table** 2a). Amongst DLBCL, those with one comorbidity or multimorbidity had 1.5 (95% CI: 1.41 – 1.66, p<0.001) or 1.9 (95% CI: 1.78 – 2.06, p<0.001) times the mortality rate, compared to no comorbidity (**Table** 2b).

The unadjusted mortality rate of death increased with each increase in deprivation level: those living in the most deprived areas had 1.5 (95% CI: 1.22 – 1.82, p<0.001) and 1.3 (95% CI: 1.22 – 1.40, p<0.001) times the mortality rate compared to those living in the least deprived areas, for FL and DLBCL, respectively.

For peer review only

Table 2A: One-year unadjusted mortality rates and rate ratios by sex, age, deprivation, ethnicity, and comorbidity status amongst patients with **Follicular lymphoma** in England between 2005-2015 (n = 14,043; 1,087 deaths at one-year).

	Deaths / person years	Mortality rate* (95% CI)	Mortality RR	95% CI	p-value
**One-year mortality (n = 1,087)**					
**Sex**					
Male	526/6218.12	8.5 (7.77 – 9.21)	Ref		
Female	561/7225.93	7.8 (7.15 – 8.43)	0.92	(0.82 – 1.03)	0.157
**Age at diagnosis (y)** †					
10-year increase	-	-	2.20	(2.08 – 2.34)	<0.001
**Age at diagnosis (y)**					
45-54	46/2300.66	2.0 (1.50 – 2.67)	Ref		
55-64	129/3926.24	3.3 (2.77 – 3.90)	1.64	(1.17 – 2.30)	0.004
65-74	274/4157.52	6.6 (5.86 – 7.42)	3.30	(2.41 – 4.50)	<0.001
75+	638/3059.62	20.9 (19.30 – 22.54)	10.43	(7.73 – 14.07)	<0.001
**Deprivation**					
Least deprived	211/3175.55	6.7 (5.81 – 7.60)	Ref		
2	227/3102.18	7.3 (6.43 – 8.33)	1.10	(0.91 – 1.33)	0.313
3	230/2884.20	8.0 (7.01 – 9.08)	1.20	(1.00 – 1.45)	0.055
4	240/2471.30	9.7 (8.56 – 11.02)	1.46	(1.23 – 1.76)	<0.001
Most deprived	179/1810.81	9.9 (8.54 – 11.45)	1.49	(1.22 – 1.82)	<0.001
**Ethnicity**					
White	742/9712.99	7.6 (7.11 – 8.21)	Ref		
Other	21/450.96	4.7 (3.04 – 7.14)	0.61	(0.40 – 0.94)	0.024
**Comorbidity status**					
None	895/12412.44	7.5 (7.03 – 7.97)	Ref		
One	77/577.89	13.3 (10.66 – 16.66)	1.85	(1.46 – 2.33)	<0.001
Multimorbidity	115/453.71	25.4 (21.11 – 30.43)	3.52	(2.90 – 4.27)	<0.001

* per 100 person-years
† continuous form of age (for each 10-year increase in age)
CI: Confidence interval, **RR:** Rate Ratio, **Missing values:** ethnicity n(%) = 3,472 (24.7%)

For peer review only

Table 2B: One-year unadjusted mortality rates by sex, age, deprivation, ethnicity, and comorbidity status amongst patients with **DLBCL** in England between 2005-2015 (n = 27,379; 9,095 deaths at One-year).

	Deaths/person years	Mortality rate* (95% CI)	Mortality RR	95% CI	p-value
**One-year mortality (n = 9,095)**					
**Sex**					
Male	4867/11318.43	43.0 (41.81 – 44.23)	Ref	Ref	Ref
Female	4228/9784.59	43.2 (41.93 – 44.53)	1.01	(0.96 – 1.05)	0.817
**Age at diagnosis (y)** †					
10-year increase	-	-	1.50	(1.47 – 1.53)	<0.001
**Age at diagnosis (y)**					
45-54	430/2615.81	16.4 (14.96 – 18.07)	Ref	Ref	Ref
55-64	1074/4937.54	21.8 (20.49 – 23.09)	1.32	(1.18 – 1.48)	<0.001
65-74	2365/6604.47	35.8 (34.39 – 37.28)	2.18	(1.97 – 2.41)	<0.001
75+	5226/6945.19	75.2 (73.23 – 77.31)	4.58	(4.15 – 5.05)	<0.001
**Deprivation**					
Least deprived	1765/4684.43	37.7 (35.96 – 38.48)	Ref		
2	1955/4898.09	39.9 (38.18 – 41.72)	1.06	(0.99 – 1.13)	0.079
3	1948/4424.61	44.0 (42.11 – 46.03)	1.17	(1.10 – 1.25)	<0.001
4	1908/4017.48	47.5 (45.41 – 49.67)	1.26	(1.18 – 1.35)	<0.001
Most deprived	1519/3078.40	49.3 (46.92 – 51.89)	1.31	(1.22 – 1.40)	<0.001
**Ethnicity**					
White	6351/15900.73	39.9 (38.97 – 40.94)	Ref		
Other	270/808.61	33.4 (29.64 – 37.62)	0.84	(0.74 – 0.94)	0.004
**Comorbidity status**					
None	7648/19007.45	40.2 (39.35 – 41.15)	Ref		
One	666/1081.20	61.6 (57.09 – 66.46)	1.53	(1.41 – 1.66)	<0.001
Multimorbidity	781/1014.36	77.0 (71.78 – 82.59)	1.91	(1.78 – 2.06)	<0.001

* per 100 person-years
† continuous form of age (for each 10-year increase in age)
CI: Confidence interval, **RR**: Rate Ratio, **Missing values**: ethnicity n(%) = 6,191 (22.6%)

Table 3 shows, before and after multiple imputation, the mortality hazard amongst DLBCL and FL patients at 1 year adjusted for comorbidity status, sex, age, deprivation and ethnicity. After multiple imputation, patients with multimorbidity had 2.2 (CI 1.78 – 2.64, p<0.001) and 1.4 (CI 1.34 – 1.55, p<0.001) times the mortality hazard compared to those without a comorbidity, for FL and DLBCL, respectively. Patients in more deprived areas had 1.5 (CI 1.23 – 1.84, p<0.001) and 1.3 (CI 1.21 – 1.40, p<0.001) times the mortality hazard compared to those living in the least deprived areas, for FL and DLBCL, respectively. There was evidence of a linear trend in mortality hazard by deprivation level for FL (p<0.001) and DLBCL (p<0.001). The direction and magnitude of the hazard ratios after multiple imputation were similar to complete case analysis.

For peer review only

Table 3: Adjusted hazard ratios of death (before and after multiple imputation) for all patient characteristics amongst patients with (**A**) Follicular or (**B**) DLBCL in England between 2005-2015.

	**Complete Case**			**After multiple imputation**		
	**HR***	**95% CI**	**p-value**	**HR***	**95% CI**	**p-value**
**(A) Follicular**						
**Sex**						
Male	Ref	Ref	-	Ref	Ref	-
Female	1.08	0.85 – 1.38	0.540	0.84	0.74 – 0.94	<0.001
**Comorbidity status**						
None	Ref	Ref	-	Ref	Ref	-
One	1.52	1.15 – 2.03	<0.001	1.28	1.01 – 1.61	<0.041
Multimorbidity	2.36	1.85 – 3.02	<0.001	2.17	1.78 – 2.64	<0.001
**Deprivation†**						
Least	Ref	Ref	-	Ref	Ref	-
2	1.03	0.70 – 1.53	0.873	1.09	0.90 – 1.31	0.378
3	1.47	1.00 – 2.16	0.051	1.15	0.95 – 1.39	0.143
4	1.30	0.87 – 1.94	0.200	1.44	1.19 – 1.73	<0.001
Most	1.63	1.11 – 2.41	0.013	1.50	1.23 – 1.84	<0.001
**Ethnicity**						
White	Ref	Ref	-	Ref	Ref	-
Other	0.49	0.27 – 0.88	0.017	0.64	0.40 – 1.01	0.053
**(B) Diffuse large B-cell**						
**Sex**						
Male	Ref	Ref	-	Ref	Ref	-
Female	0.91	0.84 – 0.99	0.170	0.90	0.86 – 0.93	<0.001
**Comorbidity status**						
None	Ref	Ref	-	Ref	Ref	-
One	1.29	1.18 – 1.42		1.24	1.15 – 1.35	<0.001
Multimorbidity	1.61	1.47 – 1.76	<0.001	1.44	1.34 – 1.55	<0.001
**Deprivation‡**						
Least	Ref	Ref	-	Ref	Ref	-
2	1.17	1.03 – 1.33	0.013	1.05	0.98 – 1.12	0.155
3	1.15	1.01 – 1.30	0.029	1.13	1.06 – 1.20	<0.001
4	1.25	1.11 – 1.42	<0.001	1.23	1.15 – 1.31	<0.001
Most	1.32	1.16 – 1.51	<0.001	1.30	1.21 – 1.40	<0.001
**Ethnicity**						
White	Ref	Ref	-	Ref	Ref	-
Other	0.93	0.77 – 1.13	0.463	1.03	0.91 – 1.16	0.678

CI: Confidence interval, **HR**: Hazard Ratio
Missing values: **(A)** ethnicity n(%) = 3,472 (24.7%), **(B)** ethnicity n(%) = 6,191 (22.6%)
* Adjusted for sex, comorbidity status, deprivation, ethnicity and the restricted cubic splines of age
† ‡ Likelihood ratio test for the overall effect of deprivation (p<0.001)

For peer review only

Table 4 shows the linearly combined effect[14] between comorbidity status and deprivation on short-term mortality by DLBCL and FL. Overall, at 1 year since diagnosis, amongst FL (**Table** 4a), patients who were most deprived with multimorbidity have 3.26 (CI 2.48 – 4.28) times higher short-term mortality hazard than patients without comorbidities and least deprived. Amongst DLBCL (**Table** 4b), and for the same comparison, the short-term mortality hazard was 1.88 (CI 1.70 – 2.07) times higher at 1 year.

Table 4: Linearly combined adjusted hazard ratio of comorbidity status with deprivation level on short-term mortality (after multiple imputation) amongst **[A]** FL (deaths at 1 year: n = 1,087) and **[B]** DLBCL (1 year: n = 9,095) for patients in England from 2005-2015.

	**Comorbidity status**		
	**None**<br>**HR* (95% CI)**	**One**<br>**HR* (95% CI)**	**Multimorbidity**<br>**HR* (95% CI)**
**[A] Follicular**			
**Deprivation**			
Least deprived	Ref	1.28 (1.01 – 1.61)	2.17 (1.78 – 2.64)
2	1.09 (0.90 – 1.31)	1.39 (1.03 – 1.88)	2.36 (1.80 – 3.10)
3	1.15 (0.95 – 1.39)	1.47 (1.09 – 1.98)	2.50 (1.91 – 3.26)
4	1.44 (1.19 – 1.73)	1.84 (1.36 – 2.47)	3.12 (2.40 – 4.06)
Most deprived	1.50 (1.23 – 1.84)	1.92 (1.42 – 2.59)	3.26 (2.48 – 4.28)
**[B] DLBCL**			
**Deprivation**			
Least deprived	Ref	1.24 (1.15 – 1.35)	1.44 (1.34 – 1.55)
2	1.05 (0.98 – 1.12)	1.30 (1.18 – 1.44)	1.51 (1.37 – 1.67)
3	1.13 (1.06 – 1.20)	1.40 (1.27 – 1.55)	1.63 (1.48 – 1.80)
4	1.23 (1.15 – 1.31)	1.52 (1.38 – 1.69)	1.78 (1.61 – 1.95)
Most deprived	1.30 (1.21 – 1.40)	1.62 (1.46 – 1.80)	1.88 (1.70 – 2.07)

CI: Confidence interval. **HR**: Hazard Ratio.
*Adjusted for sex, ethnicity and the restricted cubic splines of age.

For peer review only

Figure 2 shows the unadjusted (Nelson-Aalen non-parametric estimate) and standardised risks of death up to 1 year since diagnosis for FL and DLBCL by comorbidity status and deprivation. **Supplementary Tables** S4a and S4b show results of complete case and after multiple imputation. Standardised to age, sex, deprivation and ethnicity, the risk of death over the first year was consistently higher amongst those with multimorbidity compared to those with one comorbidity or none. For both FL and DLBCL, the unadjusted analysis showed that patients with multimorbidity had consistently higher cumulative incidence of death compared to those with one comorbidity or none (log rank test $p<0.001$).

For peer review only

For peer review only - http://bmjopen.bmj.com/site/about/guidelines.xhtml

Discussion

We aimed to explore the association between comorbidity status, and deprivation and their combination, on short-term mortality for patients with FL or DLBCL. We found that multimorbidity and deprivation and their combined effect are strong independent predictors of short-term mortality amongst patients with DLBCL and FL in England during 2005-2015.

To our knowledge, this is the first study that investigates the association between multimorbidity, and deprivation, on short-term mortality amongst patients with DLBCL and FL in England. Despite the scarcity of research within England, our findings are consistent with previous evidence from other countries. A Swedish study found that higher comorbidity status was independently associated with a higher risk of mortality amongst patients with diffuse large B-cell lymphoma.[31] Additionally, more deprived, compared to least deprived, patients had a higher risk of DLBCL-related mortality and there was evidence of a significant linear trend across the quintiles of deprivation. A Danish study found that higher comorbidity status was independently associated with shorter survival lengths amongst patients with any type of NHL (Hazard Ratio 1.60, CI 1.45-1.75).[32] However, these studies used a non-cancer specific comorbidity score, which underperforms (in comparison to cancer-specific scores) when using predictive models for short-term outcomes.[20] These studies suggest that the effect of comorbidity mainly occurs prior to, and shortly after, cancer diagnosis. Further studies could assess the effect of these prognostic factors on longer term survival from DLBCL or FL, using deprivation-specific life tables to minimise the inaccuracy of expected mortality when life tables are not also stratified by comorbidity status.[33]

As the association between deprivation and NHL survival is not studied as widely as solid tumours, it was unclear whether there was an association between deprivation and short-term mortality for haematological malignancies. Previous studies have described a deprivation-gap in survival comparing the least- to most-deprived,[8,10,34] but have not assessed the association. Although explored for Hodgkin's lymphoma,[35] to our knowledge, this is the first study to explore the association between deprivation and short-term mortality for NHL in England: our study provides evidence of a strong and independent association.

For peer review only - http://bmjopen.bmj.com/site/about/guidelines.xhtml

There are several dynamics that may explain the association observed in this study. Firstly, the presence of a comorbidity is known to affect the timely diagnosis of DLBLC and FL,[36] such that comorbidities presenting with similar symptoms to DLBLC or FL may delay the diagnosis and dissimilar symptoms may hasten the diagnosis. Moreover, the prevalence of comorbidities increases with age, and amongst older patients with DLBCL and FL this prevalence is consistently over 60%,[37,38] which may partly explain the delay in diagnosis amongst older ages. Further research is needed to identify comorbidities that alter the timely diagnosis

Secondly, guidelines of lymphoma management focus on a single-disease standard regimen, but there is little guidance on multi-disease management.[39] A systematic review found the majority of patients with a comorbidity did not receive the standard regimen and were allocated alternative, less-intense treatments.[40] Cancer care could be improved by defining clear guidelines that recommend a comorbidity-specific treatment regimen and provide an accurate definition, and a measure, of the dose-intensity.

Thirdly, differences in access to treatments, or risk of adverse effects, may partly explain the multimorbidity gap in survival from DLBCL and FL; clinicians may abstain from allocating a treatment associated with a higher risk of adverse events because it can exacerbate the complex management of cancer care. Patients without comorbidities, after receiving standard treatment regimens, still experience an increased risk of cardiovascular events.[41] A first-line standard treatment for DLBCL and FL is a combination of chemotherapy and immunotherapies, such as rituximab, and is known to be effective for those of an advanced age. Rituximab is often used in combination with anthracyclines (e.g. doxorubicin), which is associated with an increase in the incidence of adverse events (e.g. cardiotoxicity) commonly in the form of congestive heart failure.[42]

Lastly, the association between deprivation and short-term mortality, that is not explained by patient characteristics, might be explained by the association between deprivation and use of emergency services or population density,[43,44] or between population density and the use of emergency services.[45] For example, population dense areas may accumulate high demands that current facilities of healthcare services are unable to accommodate. Therefore, emergency services (e.g., emergency diagnostic route), which is associated with a

For peer review only

late stage of cancer, may explain the higher mortality hazard observed amongst more deprived patients. Further research could investigate the demand and availability of healthcare services in densely populated areas.

The strengths of this study include the large sample size within a database of high-quality population-based clinical records with a high national coverage. We linked clinical records with the HES database, which encompasses all patients in England with a diagnosis of DLBCL and FL between 2005 and 2013. The objective data sources provide information on patients that is gathered prospectively. Furthermore, the standardised risk provides an interpretation of the risk of death that is averaged over the entire population.

Due to data availability, our study has some limitations. Firstly, we did not include tumour stage, route to diagnosis (e.g., general practitioner referral), or treatment plan; consequently, further research is needed to dissect the effects of comorbidity, stage and treatment on survival. Since tumour stage, route and treatment allocation are considered to be on the causal pathway between comorbidity status and short-term mortality, causal inference mediation analysis is required to estimate the proportion of the effect of comorbidity status on survival that is explained by said mediators.

Secondly, recent research highlights the interest in using individual-level socioeconomic measures for assessing patient health outcomes in addition to area-lvel measures of deprivation.[46] However, information on individual-level socioeconomic measures were unavailable, so we used only an area-level measure of socioeconomic status, which encapsulates the multidimensional composition of a patient's deprivation level in addition to the contextual level.[21] Furthermore, there is better concordance between area- and individual-level measures of education when assessing patient health outcomes.[46] The observed deprivation level of a patient in our study is likely to be consistent had they been diagnosed at a different time; this is because deprivation scores have a high concordance between updates (i.e., IMD of 2007, 2010, and 2015).[21] Our results are comparable to studies using this area-level measure of deprivation.

Thirdly, Hospital Episode Statistics (HES) data contains information on all patients admitted to a hospital (secondary care) in England. It is possible that some comorbidities were not observed because they were

For peer review only

diagnosed, and treated, during primary care (e.g., general practitioner consultations). However, the Royal College of Surgeons' comorbidity index, amongst other indices, are constructed based on the impact of the comorbidity on the risk of mortality; in other words, severe comorbidities that require hospitalisation. Comorbidities of the RCS comorbidity index are those that often require hospitalisation, leading to a record within HES data. Previous research has shown that combining primary care records to secondary care data identifies a greater proportion of comorbidity within the population; however, the inclusion of comorbidities identified from primary care records does not have a large effect on predicted cancer survival beyond results obtained using secondary care data.[47]

Lastly, as complete case analysis may lead to selection bias, we performed multiple imputation under a missing at random assumption. We obtained the same conclusions under a complete case analysis and after multiple imputation. Since the missing at random assumption is untestable,[48] further work could conduct a sensitivity analysis to departures from the missing at random assumption, through techniques for imputing under a missing not at random assumption.[49]

In conclusion, multimorbidity and deprivation, combined and independently, are strong predictors of an increased risk of short-term mortality at 1 year since diagnosis amongst patients with DLBCL or FL in England. Therefore, public health prevention strategies are needed to reduce the short-term mortality gap due to socioeconomic inequalities and comorbidities amongst NHL patients.

For peer review only

Figure Legends

Figure 1: Overlap plots for the density of predicted probabilities of comorbidity status amongst patients (n=41,422), aged 45-99, in England diagnosed with non-Hodgkin lymphoma during 2005-2013. Propensity score: relates to the predicted probability of having any comorbidity level as measured by a multinomial logistic regression model conditioning on the independent variables (i.e., age at diagnosis, sex, deprivation level, and ethnicity).

Figure 2. Risk of short-term mortality for Follicular lymphoma (n=14,043) and DLBCL (n=27,379) by comorbidity status and deprivation level in England between 2005-2015. (Solid: Aalen-Nelson approach, Dash: standardised to the empirical distribution of age, sex, and ethnicity).

For peer review only

For peer review only - http://bmjopen.bmj.com/site/about/guidelines.xhtml

Additional information

Acknowledgements: We thank Professor Paul Lambert (Leicester University, UK) for his advice on using the *stpm2* Stata software package in combination with multiple imputation. We also thank Adrian Turculet (Data Manager, LSHTM Inequalities in Cancer Outcomes Network), for his support with the data linkage.

Author's contributions: MS, ENN and BR contributed to the conception of the study and designed the study. ENN, BR, CL, and MALF provided advice on statistical methods. MS conducted the analyses of the data and prepared the draft of the manuscript, tables and figures. ENN and BR supervised the study and provided comments on the manuscript draft. ENN, BR, MALF, CL, SBM, ABe and ABo provided comments on the final draft of the manuscript. All authors read and approved the final manuscript.

Ethics approval and consent to participate: We obtained the statutory approvals required for this research from the Confidentiality Advisory Group (CAG) of the Health Research Authority (HRA): PIAG 1–05(c) 2007. Ethical approval was obtained from the Research Ethics Committee (REC) of the Health Research Authority (HRA): 07/MRE01/52. This work uses data provided by patients and collected by the National Health Service as part of their care and support. We used anonymised National Cancer Registry and Hospital Episode Statistics data. No consent to participate was sought from patients.

Availability of data and materials: The data that support the findings of this study are available via application to the Public Health England Office for Data Release, but restrictions apply to the availability of these data.

Funding: This research was funded by Cancer Research UK grant number C7923/A18525. The authors declare no support from any organisations for the submitted work. The design of the study, the analyses and the writing of the manuscript were solely the responsibility of the authors. The findings and conclusions in this manuscript are those of the authors and do not necessarily represent the views of Cancer Research UK.

Competing interests: The authors declare there are no competing interests.

For peer review only

References

1. Office for National Statistics. *Cancer registration statistics, England: 2017*. (2019).
2. Cancer Research UK. Non-Hodgkin lymphoma statistics. (2017). Available at: https://www.cancerresearchuk.org/health-professional/cancer-statistics/statistics-by-cancer-type/non-hodgkin-lymphoma. (Accessed: 24th April 2020)
3. Quaresma, M., Coleman, M. P. & Rachet, B. 40-year trends in an index of survival for all cancers combined and survival adjusted for age and sex for each cancer in England and Wales, 1971–2011: a population-based study. *Lancet* **385**, 1206–1218 (2015).
4. Shankland, K. R., Armitage, J. O. & Hancock, B. W. Non-Hodgkin lymphoma. *Lancet* **380**, 848–857 (2012).
5. Epidemiology & Cancer Statistics Group University of York. Haematological Malignancy Research Network. (2016).
6. Allemani, C. *et al.* Global surveillance of trends in cancer survival 2000–14 (CONCORD-3): analysis of individual records for 37,513,025 patients diagnosed with one of 18 cancers from 322 population-based registries in 71 countries. *Lancet* **391**, 1023–1075 (2018).
7. Thomson, C. S. & Forman, D. Cancer survival in England and the influence of early diagnosis: what can we learn from recent EUROCARE results? *Br. J. Cancer* **101**, S102–S109 (2009).
8. Rachet, B., Mitry, E., Shah, A., Cooper, N. & Coleman, M. P. Survival from non-Hodgkin lymphoma in England and Wales up to 2001. *Br. J. Cancer* **99**, S104–S106 (2008).
9. National Institute for Health and Care Excellence. *Haematological cancers: improving outcomes*. (2016).
10. Exarchakou, A., Rachet, B., Belot, A., Maringe, C. & Coleman, M. P. Impact of national cancer policies on cancer survival trends and socioeconomic inequalities in England, 1996-2013: population based study. *BMJ* **360**, k764–k764 (2018).
11. Porta, M. *A Dictionary of Epidemiology*. (Oxford University Press, 2014).
12. Salika, T., Lyratzopoulos, G., Whitaker, K. L., Waller, J. & Renzi, C. Do comorbidities influence help-seeking for cancer alarm symptoms? A population-based survey in England. *J. Public Health (Bangkok).* **40**, 340–349 (2017).

For peer review only - http://bmjopen.bmj.com/site/about/guidelines.xhtml

13. Fowler, H. *et al.* Comorbidity prevalence among cancer patients: a population-based cohort study of four cancers. *BMC Cancer* **20**, 2 (2020).

14. Luque-Fernandez, M. A. *et al.* Multimorbidity and short-term overall mortality among colorectal cancer patients in Spain: A population-based cohort study. *Eur. J. Cancer* **129**, 4–14 (2020).

15. International Agency for Research on Cancer. International Classification of Diseases for Oncology. (2013). Available at: http://codes.iarc.fr/. (Accessed: 4th October 2019)

16. gov.uk. National Cancer Registry and Analysis Service. (2017). Available at: https://www.gov.uk/guidance/national-cancer-registration-and-analysis-service-ncras. (Accessed: 4th October 2019)

17. Maringe, C., Fowler, H., Rachet, B. & Luque-Fernandez, M. A. Reproducibility, reliability and validity of population-based administrative health data for the assessment of cancer non-related comorbidities. *PLoS One* **12**, e0172814 (2017).

18. Hernán, M. Á. & Robins, J. M. Estimating causal effects from epidemiological data. *J. Epidemiol. Community Health* **60**, 578–586 (2006).

19. Charlson, M. E., Pompei, P., Ales, K. L. & MacKenzie, C. R. A new method of classifying prognostic comorbidity in longitudinal studies: Development and validation. *J. Chronic Dis.* **40**, 373–383 (1987).

20. Armitage, J. N. & van der Meulen, J. H. Identifying co-morbidity in surgical patients using administrative data with the Royal College of Surgeons Charlson Score. *Br J Surg* **97**, 772–781 (2010).

21. gov.uk. Indices of Multiple Deprivation. (2015). Available at: https://www.gov.uk/government/statistics/english-indices-of-deprivation-2015. (Accessed: 4th October 2019)

22. National Health Service: data dictionary. Lower Super Output Area. (2018). Available at: https://www.datadictionary.nhs.uk/data_dictionary. (Accessed: 4th October 2019)

23. Armitage, P., Berry, G. & Matthews, J. *Statistical methods in medical research*. (Blackwell Science, 2002).

24. Royston, P. & Lambert, P. *Flexible Parametric Survival Analysis Using Stata: Beyond the Cox Model.* (2011).

25. Robins, J. M., Hernán, M. Á. & Brumback, B. Marginal Structural Models and Causal Inference in

For peer review only

Epidemiology. *Epidemiology* **11**, (2000).

26. Cole, S. & Hernan, M. A. Adjusted survival curves with inverse probability weights. *Comput. Methods Programs Biomed.* **75**, 45–49 (2004).

27. Dupont, W. D. *Statistical Modeling for Biomedical Researchers: A Simple Introduction to the Analysis of Complex Data*. (Cambridge University Press, 2009).

28. Armitage, J. O. Staging Non-Hodgkin Lymphoma. *CA. Cancer J. Clin.* **55**, 368–376 (2005).

29. Halpern, M. T. *et al.* Association of insurance status and ethnicity with cancer stage at diagnosis for 12 cancer sites: a retrospective analysis. *Lancet Oncol.* **9**, 222–231 (2008).

30. Little, R. J. & Rubin, D. B. *Statistical Analysis with Missing Data*. (John Wiley & Sons, Inc., 1987).

31. Wästerlid, T. *et al.* Impact of comorbidity on disease characteristics, treatment intent and outcome in diffuse large B-cell lymphoma: a Swedish lymphoma register study. *J. Intern. Med.* **285**, 455–468 (2019).

32. Frederiksen, B. L., Dalton, S. O., Osler, M., Steding-Jessen, M. & de Nully Brown, P. Socioeconomic position, treatment, and survival of non-Hodgkin lymphoma in Denmark--a nationwide study. *Br J Cancer* **106**, 988–995 (2012).

33. Rubio, F. J., Rachet, B., Giorgi, R., Maringe, C. & Belot, A. On models for the estimation of the excess mortality hazard in case of insufficiently stratified life tables. *Biostatistics* **22**, 51–67 (2021).

34. Rachet, B. *et al.* Socioeconomic inequalities in cancer survival in England after the NHS cancer plan. *Br J Cancer* **103**, 446–453 (2010).

35. Rafiq, M. *et al.* Socioeconomic deprivation and regional variation in Hodgkin's lymphoma incidence in the UK: A population-based cohort study of 10 million individuals. *BMJ Open* **9**, (2019).

36. Howell, D. A. *et al.* Time-to-diagnosis and symptoms of myeloma, lymphomas and leukaemias: a report from the Haematological Malignancy Research Network. *BMC Blood Disord.* **13**, 9 (2013).

37. van Spronsen, D. J., Janssen-Heijnen, M. L., Breed, W. P. & Coebergh, J. W. Prevalence of co-morbidity and its relationship to treatment among unselected patients with Hodgkin's disease and non-Hodgkin's lymphoma, 1993-1996. *Ann Hematol* **78**, 315–319 (1999).

38. Janssen-Heijnen, M. L. *et al.* A population-based study of severity of comorbidity among patients with non-Hodgkin's lymphoma: prognostic impact independent of International Prognostic Index. *Br J*

For peer review only

Haematol **129**, 597–606 (2005).

39. National Institute for Health and Care Excellence. *Non-Hodgkin's lymphoma: diagnosis and management*. (National Institute for Health and Care Excellence, 2016).

40. Terret, C., Albrand, G., Rainfray, M. & Soubeyran, P. Impact of comorbidities on the treatment of non-Hodgkin's lymphoma: a systematic review. *Expert Rev. Hematol.* **8**, 329–341 (2015).

41. Linschoten, M. *et al.* Cardiovascular adverse events in patients with non-Hodgkin lymphoma treated with first-line cyclophosphamide, doxorubicin, vincristine, and prednisone (CHOP) or CHOP with rituximab (R-CHOP): a systematic review and meta-analysis. *Lancet Haematol.* **7**, e295–e308 (2020).

42. McGowan, J. V *et al.* Anthracycline Chemotherapy and Cardiotoxicity. *Cardiovasc. Drugs Ther.* **31**, 63–75 (2017).

43. Carlisle, R., Groom, L. M., Avery, A. J., Boot, D. & Earwicker, S. Relation of Out of Hours Activity by General Practice and Accident and Emergency Services with Deprivation in Nottingham: Longitudinal Survey on JSTOR. *BMJ Br. Med. J.* **316**, 520–523 (1998).

44. Venerandi, A., Quattrone, G. & Capra, L. A scalable method to quantify the relationship between urban form and socio-economic indexes. *EPJ Data Sci.* **7**, 4 (2018).

45. Peacock, P. J. & Peacock, J. L. Emergency call work-load, deprivation and population density: An investigation into ambulance services across England. *J. Public Health (Bangkok).* **28**, 111–115 (2006).

46. Ingleby, F. C. *et al.* Assessment of the concordance between individual-level and area-level measures of socio-economic deprivation in a cancer patient cohort in England and Wales. *BMJ Open* **10**, e041714 (2020).

47. Crooks, C. J., West, J. & Card, T. R. A comparison of the recording of comorbidity in primary and secondary care by using the Charlson Index to predict short-term and long-term survival in a routine linked data cohort. *BMJ Open* **5**, e007974 (2015).

48. Carpenter, J. R. & Kenward, M. G. *Multiple Imputation and Its Application*. (John Wiley & Sons, Ltd, 2013).

49. Gachau, S. *et al.* Handling missing data in modelling quality of clinician-prescribed routine care: Sensitivity analysis of departure from Missing at Random (MAR) assumption. *Stat. Methods Med. Res.*

For peer review only

29, 3076 (2020).

For peer review only

For peer review only - http://bmjopen.bmj.com/site/about/guidelines.xhtml

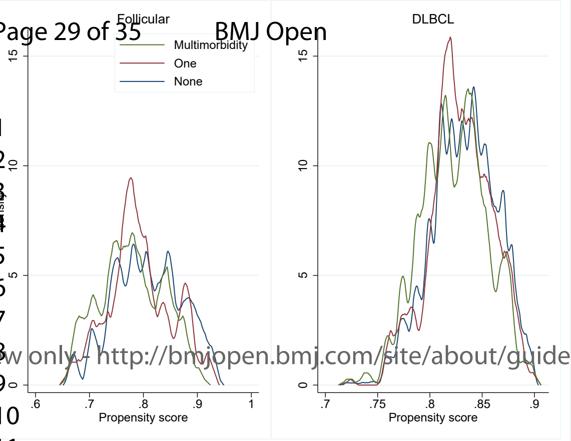

1
2
3
4
5
6
7
8
9
10
11

view only - http://bmjopen.bmj.com/site/about/guide

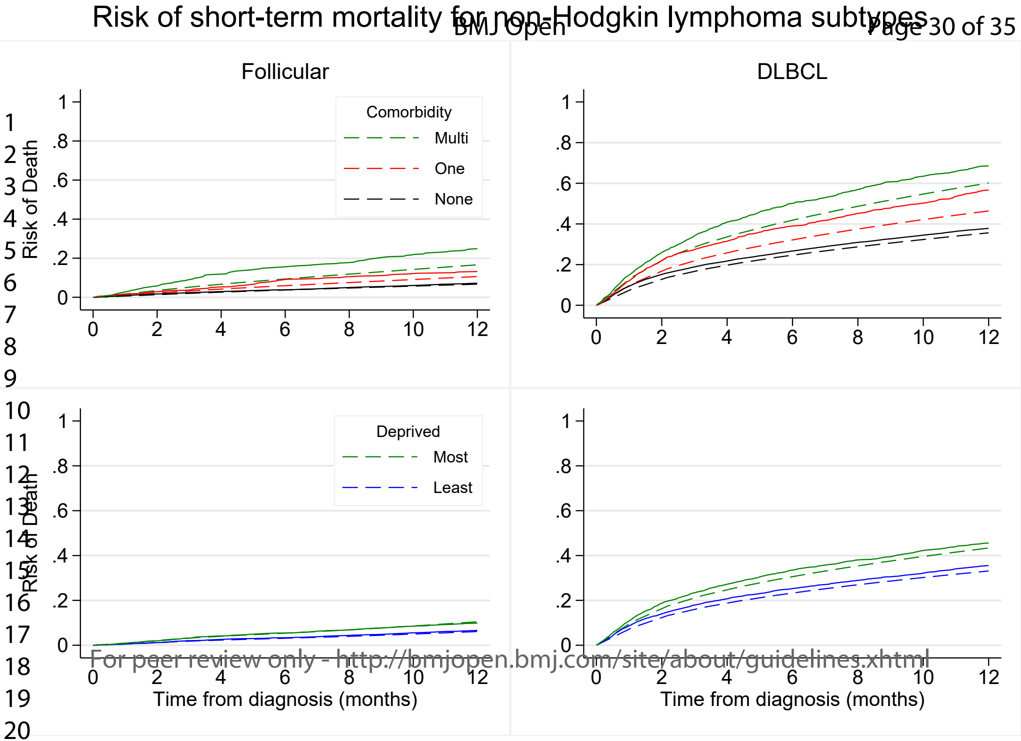

For peer review only - http://bmjopen.bmj.com/site/about/guidelines.xhtml

Supplementary Tables

This page is intentionally blank. Please move to next page.

For peer review only

Supplementary Table S1. Distribution of non-Hodgkin lymphoma subtypes for patients in England diagnosed from 2005-2013, with respective morphology and topography ICD-O-3 codes.

Index	Site group (subtype)	Progression	Topography	Morphology	n	%
1	CLL/SLL*	Indolent	C82.0-C85.9	9670, 9823	3,875	5.08
2	Waldenstrom macroglobulinemia	Indolent	C82.0-C85.9	9761	2,398	3.14
3	Mantle cell	Indolent	C82.0-C85.9	9673	3,458	4.53
4	Diffuse large B-cell	Aggressive	C82.0-C85.9	9680, 9688, 9737-9738	27,379	35.89
5	Burkitt	Aggressive	C82.0-C85.9	9687, 9826	695	0.91
6	Follicular	Indolent	C82.0-C85.9	9690-9691, 9695, 9698	14,043	18.41
7	Mature T-cell	Aggressive	C82.0-C85.9	9702	5,127	6.72
8	Marginal zone B-cell	Indolent	C82.0-C85.9	9689, 9699, 9760, 9764, 9699	4,277	5.61
**Subtotal**					**61,252**	**80.30**
9	Not Otherwise Specified	n/a	C82.0-C85.9	9591, 9675, 9735	9,581	12.56
10	Other***	n/a	C82.0-C85.9	9591, 9675, 9735	5,449	7.14
**Total**					**76,282**	**100.00****

n/a – not applicable; there was no subtype information
* Chronic lymphocytic leukaemia/Small-cell lymphocytic lymphoma
** Percentages may not equate to 100.0% due to rounding
*** The morphology code specifies these patients are diagnosed with NHL. However, the description states 'other'; these patients are classified similarly to 'Not Otherwise Specified'.

For peer review only - http://bmjopen.bmj.com/site/about/guidelines.xhtml

Supplementary Table S2: Comorbidities and their diagnostic ICD-10 codes

Comorbidity	ICD-10
Myocardial infarction	I21.x, I22.x, I25.2
Congestive heart failure	I11.0, I13.0, I13.2, I25.5, I42.0, I42.5–I42.9, I43.x, I50.x, P29.0
Peripheral vascular disease	I70.x, I71.x, I73.1, I73.8, I73.9, I77.1, I79.0, I79.2, K55.1, K55.8, K55.9, Z95.8, Z95.9
Cerebrovascular disease	G45.x, G46.x, H34.0, I60.x–I69.x
Dementia	F00.x–F03.x, F05.1, G30.x, G31.1
Chronic obstructive pulmonary disease	I27.9, J40.x–J47.x, J60.x–J67.x, J68.4, J70.1, J70.3
Rheumatic disease	M05.x, M06.x, M31.5, M32.x–M34.x, M35.1, M35.3, M36.0
Liver disease	B18.x, K70.0–K70.3, K70.9, K71.3–K71.5, K71.7, K73.x, K74.x, K76.0, K76.2–K76.4, K76.8, K76.9, Z94.4, K71.1, K72.1, K72.9, K76.5, K76.6, K76.7, I85.0, I85.9, I86.4, I98.2, K70.4,
Diabetes without chronic complication	E10.0, E10.1, E10.6, E10.8, E10.9, E11.0, E11.1, E11.6, E11.8, E11.9, E12.0, E12.1, E12.6, E12.8, E12.9, E13.0, E13.1, E13.6, E13.8, E13.9, E14.0, E14.1, E14.6, E14.8, E14.9
Diabetes with chronic complication	E10.7, E11.2–E11.5, E11.7, E12.2–E12.5, E12.7, E13.2–E13.5, E13.7, E14.2–E14.5, E14.7
Hemiplegia or paraplegia	G04.1, G11.4, G80.1, G80.2, G81.x, G82.x, G83.0–G83.4, G83.9
Renal disease	I12.0, I13.1, N03.2–N03.7, N05.2–N05.7, N18.x, N19.x, N25.0, Z49.0–Z49.2, Z94.0, Z99.2
AIDS/HIV	B20.x–B22.x, B24.x

ICD-10: International Classification of Diseases, 10th Revision
Diabetes with/without chronic complication is combined in the RCS Charlson Comorbidity Score

For peer review only - http://bmjopen.bmj.com/site/about/guidelines.xhtml

Supplementary Table S3. Sociodemographic characteristics, route of diagnosis, comorbidity status and Ann Arbor cancer stage distribution, of Follicular (n=14,043) and DLBCL (n=27,379) lymphomas in England, 2005-2013.

	Follicular	DLBCL		
	N = 14,043	N = 27,379	**OR* (95% CI)**	**p-value**
**Age** *(mean; SD)***	66.7 (11.0)	70.8 (11.3)	1.37 (1.35 – 1.40)	<0.001
**Gender**				
Male	6,512 (46.4)	14,652 (53.5)	Ref	Ref
Female	7,531 (53.6)	12,727 (46.5)	0.75 (0.72 - 0.78)	<0.001
**Deprivation**				
Least deprived	3,284 (23.4)	5,895 (21.5)	Ref	Ref
2	3,228 (23.0)	6,238 (22.8)	1.08 (1.01 – 1.14)	0.016
3	3,013 (21.5)	5,756 (21.0)	1.06 (1.00 – 1.13)	0.047
4	2,609 (18.6)	5,341 (19.5)	1.14 (1.07 – 1.22)	<0.001
Most deprived	1,909 (13.6)	4,149 (15.2)	1.21 (1.13 – 1.30)	<0.001
**Comorbidity status**				
Non	12,898 (91.9)	24,269 (88.6)	Ref	Ref
One	623 (4.4)	1,538 (5.6)	1.60 (1.45 – 1.77)	<0.001
Multimorbidity	522 (3.7)	1,572 (5.7)	1.58 (1.43 – 1.75)	<0.001
**Route**				
Elective	11,211 (86.7)	17,280 (66.3)	Ref	Ref
Emergency	1,727 (13.4)	8,799 (33.7)	3.31 (3.12 – 3.50)	<0.001
Missing	*1,105 (7.9)*	*1,300 (4.8)*	-	-
**Stage**				
I	848 (26.6)	1,680 (27.8)	Ref	Ref
II	473 (14.8)	1,169 (19.3)	1.25 (1.09 – 1.43)	0.001
III	822 (25.7)	1,046 (17.3)	0.64 (0.57 – 0.73)	<0.001
IV	1050 (32.9)	2,152 (35.6)	1.03 (0.93 – 1.16)	0.548
Missing	*10,850 (77.3)*	*21,332 (77.9)*	-	-
**Ethnicity**				
White	10,110 (95.6)	20,204 (95.4)	Ref	Ref
Other	461 (4.4)	984 (4.6)	1.07 (0.95 – 1.20)	0.254
Missing	*3,472 (24.7)*	*6,191 (22.6)*	-	-

* Odds ratio from a complete case analysis comparing the odds of DLBCL to Follicular lymphoma
** 10-year increase in age
P-values calculated from Wald tests

For peer review only

Supplementary Table S4a: Risk of short-term mortality amongst patients diagnosed with Follicular lymphomas (n=14,043) by comorbidity status in England between 2005 and 2013

	Complete Case Analysis						After Multiple Imputation					
	Comorbidity status						Comorbidity status					
	None		One		Multimorbidity		None		One		Multimorbidity	
	CH	*95% CI*	*CH*	*95% CI*	*CH*	*95% CI*	*CH*	*95% CI*	*CH*	*95% CI*	*CH*	*95% CI*
**Month**												
1	0.6	0.4 – 0.9	1.0	0.7 - 1.4	1.6	1.0 – 2.3	0.8	0.7 – 1.0	1.3	1.1 – 1.5	2.4	1.6 – 3.2
2	1.4	1.0 – 1.7	2.2	1.6 - 2.7	3.5	2.3 – 4.6	1.7	1.5 – 1.9	2.6	2.2 – 3.0	4.8	3.4 – 6.3
3	2.0	1.6 – 2.4	3.2	2.6 - 3.9	5.2	3.6 – 6.7	2.4	2.2 – 2.7	3.7	3.2 – 4.2	6.8	4.9 – 8.7
4	2.6	2.2 – 3.1	4.2	3.5 - 4.9	6.7	4.8 – 8.6	3.0	2.7 – 3.3	4.6	4.0 – 5.2	8.4	6.1 – 10.8
5	3.2	2.6 – 3.7	5.1	4.3 - 5.9	8.1	5.9 – 10.3	3.6	3.3 – 3.9	5.4	4.8 – 6.1	9.9	7.2 – 12.6
6	3.7	3.1 – 4.3	5.9	5.0 - 6.9	9.4	6.9 – 11.9	4.1	3.8 – 4.4	6.2	5.5 – 7.0	11.3	8.3 – 14.3
7	4.2	3.6 – 4.9	6.7	5.7 - 7.7	10.6	7.8 – 13.4	4.6	4.2 – 5.0	7.0	6.2 – 7.9	12.7	9.3 – 16.0
8	4.7	4.0 – 5.5	7.5	6.4 - 8.6	11.8	8.8 – 14.9	5.1	4.8 – 5.5	7.8	6.9 – 8.8	14.1	10.4 – 17.8
9	5.2	4.5 – 6.0	8.3	7.2 - 9.4	13.1	9.7 – 16.4	5.7	5.3 – 6.1	8.7	7.6 – 9.7	15.5	11.5 – 19.5
10	5.7	5.0 – 6.5	9.1	7.9 - 10.3	14.3	10.6 – 17.9	6.2	5.8 – 6.7	9.5	8.4 – 10.6	17.0	12.6 – 21.3
11	6.2	5.4 – 7.0	9.9	8.6 - 11.1	15.4	11.5 – 19.4	6.8	6.4 – 7.3	10.3	9.2 – 11.5	18.4	13.7 – 23.1
12	6.7	5.9 – 7.5	10.6	9.2 - 12.0	16.6	12.3 – 20.9	7.4	6.9 – 7.8	11.2	9.9 – 12.4	19.8	14.8 – 24.9

CH – cumulative hazard of death (e x 10^2)
95% CI – confidence interval (e x 10^2)

Supplementary Table S4b: Risk of short-term mortality amongst patients diagnosed with DLBCL (n=27,379) by comorbidity status in England between 2005 and 2013

	Complete Case Analysis						After Multiple Imputation					
	Comorbidity status						Comorbidity status					
	None		One		Multimorbidity		None		One		Multimorbidity	
	CH	*95% CI*	*CH*	*95% CI*	*CH*	*95% CI*	*CH*	*95% CI*	*CH*	*95% CI*	*CH*	*95% CI*
**Month**												
1	7.4	6.9 – 7.9	9.7	9.0 – 10.4	13.2	11.5 – 14.9	9.2	8.9 – 9.6	12.0	11.4 – 12.5	16.1	14.5 – 17.7
2	12.9	12.2 – 13.7	17.0	16.0 – 18.0	23.0	20.4 – 25.7	15.3	14.8 – 15.7	19.7	18.8 – 20.5	26.3	23.9 – 28.8
3	16.8	16.0 – 17.6	22.0	20.8 – 23.2	30.0	26.6 – 33.3	19.1	18.6 – 19.7	24.6	23.6 – 25.6	32.8	29.8 – 35.8
4	19.8	18.9 – 20.7	25.9	24.5 – 27.2	35.4	31.5 – 39.3	22.1	21.6 – 22.7	28.4	27.2 – 29.5	37.7	34.3 – 41.1
5	22.4	21.4 – 23.3	29.2	27.7 – 30.7	39.9	35.6 – 44.3	24.8	24.1 – 25.4	31.7	30.4 – 32.9	42.0	38.2 – 45.8
6	24.7	23.7 – 25.7	32.2	30.6 – 33.8	43.9	39.2 – 48.7	27.1	26.5 – 27.8	34.7	33.3 – 36.0	45.9	41.8 – 50.0
7	26.8	25.8 – 27.8	34.9	33.3 – 36.6	47.5	42.4 – 52.6	29.3	28.6 – 30.0	37.5	36.0 – 38.9	49.6	45.2 – 53.9
8	28.8	27.7 – 29.8	37.5	35.8 – 39.2	50.8	45.4 – 56.2	31.4	30.7 – 32.1	40.1	38.5 – 41.6	53.0	48.3 – 57.6
9	30.6	29.5 – 31.7	39.9	38.1 – 41.7	53.8	48.2 – 59.5	33.3	32.6 – 34.0	42.5	40.9 – 44.1	56.2	51.2 – 61.1
10	32.4	31.2 – 33.5	42.2	40.3 – 44.0	56.7	50.8 – 62.6	35.1	34.4 – 35.9	44.8	43.2 – 46.5	59.2	54.0 – 64.4
11	34.0	32.8 – 35.2	44.3	42.4 – 46.3	59.4	53.3 – 65.5	36.9	36.1 – 37.7	47.1	45.3 – 48.8	62.1	56.7 – 67.6
12	35.6	34.4 – 36.9	46.4	44.3 – 48.5	62.0	55.6 – 68.4	38.6	37.7 – 39.4	49.2	47.4 – 51.0	64.9	59.2 – 70.6

CH – cumulative hazard (e x 10^2)
95% CI – confidence interval (e x 10^2)

For peer review only

For peer review only

BMJ Open

Association between multimorbidity and socioeconomic deprivation on short-term mortality amongst patients with Diffuse Large B-cell or Follicular lymphoma in England: a nationwide cohort study

Journal:	*BMJ Open*
Manuscript ID	bmjopen-2021-049087.R2
Article Type:	Original research
Date Submitted by the Author:	25-Sep-2021
Complete List of Authors:	Smith, Matthew; London School of Hygiene & Tropical Medicine, Non-Communicable Disease Epidemiology<br>Njagi, Edmund Njeru; London School of Hygiene & Tropical Medicine<br>Belot, Aurelien; London School of Hygiene & Tropical Medicine<br>Leyrat, Clémence; London School of Hygiene & Tropical Medicine, Department of Non-Communicable Disease Epidemiology<br>Bonaventure, Audrey; University of Paris, Epidemiology of Childhood and Adolescent Cancers Team<br>Benitez Majano, Sara ; London School of Hygiene & Tropical Medicine, Department of Non-Communicable Disease Epidemiology<br>Rachet, Bernard; London School of Hygiene & Tropical Medicine, Non-Communicable Disease Epidemiology<br>Luque Fernandez, Miguel Angel; London School of Hygiene and Tropical Medicine, Epidemiology and Population Health; Instituto de Investigación Biosanitaria de Granada, Noncommunicable Disease and Cancer Epidemiology Group
**Primary Subject Heading**:	Epidemiology
Secondary Subject Heading:	Public health, Epidemiology, Global health, Oncology, Research methods
Keywords:	Epidemiology < ONCOLOGY, STATISTICS & RESEARCH METHODS, Lymphoma < HAEMATOLOGY

SCHOLARONE™
Manuscripts

I, the Submitting Author has the right to grant and does grant on behalf of all authors of the Work (as defined in the below author licence), an exclusive licence and/or a non-exclusive licence for contributions from authors who are: i) UK Crown employees; ii) where BMJ has agreed a CC-BY licence shall apply, and/or iii) in accordance with the terms applicable for US Federal Government officers or employees acting as part of their official duties; on a worldwide, perpetual, irrevocable, royalty-free basis to BMJ Publishing Group Ltd ("BMJ") its licensees and where the relevant Journal is co-owned by BMJ to the co-owners of the Journal, to publish the Work in this journal and any other BMJ products and to exploit all rights, as set out in our licence.

The Submitting Author accepts and understands that any supply made under these terms is made by BMJ to the Submitting Author unless you are acting as an employee on behalf of your employer or a postgraduate student of an affiliated institution which is paying any applicable article publishing charge ("APC") for Open Access articles. Where the Submitting Author wishes to make the Work available on an Open Access basis (and intends to pay the relevant APC), the terms of reuse of such Open Access shall be governed by a Creative Commons licence – details of these licences and which Creative Commons licence will apply to this Work are set out in our licence referred to above.

Other than as permitted in any relevant BMJ Author's Self Archiving Policies, I confirm this Work has not been accepted for publication elsewhere, is not being considered for publication elsewhere and does not duplicate material already published. I confirm all authors consent to publication of this Work and authorise the granting of this licence.

For peer review only

Title

Association between multimorbidity and socioeconomic deprivation on short-term mortality amongst patients with Diffuse Large B-cell or Follicular lymphoma in England: a nationwide cohort study

Authors

Matthew J. Smith[1*], Edmund Njeru Njagi[1], Aurélien Belot[1], Clémence Leyrat[1,2], Audrey Bonaventure[3], Sara Benitez Majano[1], Bernard Rachet[1], Miguel Angel Luque Fernandez[1,4,5]

Authors' affiliations

[1] Inequalities in Cancer Outcomes Network, Department of Non-Communicable Disease Epidemiology, London School of Hygiene and Tropical Medicine, Keppel Street, London, WC1E 7HT, UK

[2] Department of Medical Statistics, London School of Hygiene and Tropical Medicine, Keppel Street, London, WC1E 7HT, UK

[3] CRESS, Université de Paris, INSERM, UMR 1153, Epidemiology of Childhood and Adolescent Cancers Team, Villejuif, France

[4] Noncommunicable Disease and Cancer Epidemiology Group, Instituto de Investigación Biosanitaria de Granada, Ibs.GRANADA, Andalusian School of Public Health, Granada, Spain

[5] Centro de Investigación Biomédica en Red de Epidemiología y Salud Pública (CIBER of Epidemiology and Public Health, CIBERESP), Madrid, Spain

Corresponding author*

Matthew J. Smith
Keppel Street, London, WC1E 7HT, UK
ORCID: 0000-0002-8502-0056
Email: *matthew.smith1@lshtm.ac.uk*

Word count: Abstract: 193; Text: 2807; Tables: 4; Figures: 2

For peer review only

Abstract

Objectives: We aimed to assess the association between multimorbidity and deprivation on short-term mortality amongst DLBCL and FL patients in England.

Setting: The association of multimorbidity and socioeconomic deprivation on survival among patients diagnosed with Diffuse Large B-cell (DLBCL) and Follicular lymphoma (FL) in England between 2005 and 2013. We linked the English population-based cancer registry with electronic health records databases and estimated adjusted mortality rate ratios by multimorbidity and deprivation status. Using flexible hazard-based regression models, we computed DLBCL and FL standardised mortality risk by deprivation and multimorbidity at 1 year.

Results: Overall, 41,422 patients aged 45-99 years were diagnosed with DLBCL or FL in England during 2005-2015. Most deprived FL patients with multimorbidities had three times higher hazard of 1 year mortality (HR: 3.3, CI: 2.48 – 4.28, p<0.001) than least deprived patients without comorbidity; amongst DLBCL there was approximately twice the hazard (HR: 1.9, CI: 1.70– 2.07, p<0.001).

Conclusions: Multimorbidity, deprivation, and their combination, are strong and independent predictors of an increased short-term mortality risk amongst DLBCL and FL patients in England. Public health measures targeting the reduction of multimorbidity amongst most deprived DLBCL and FL patients are needed to reduce the short-term mortality gap.

Key words: Cancer epidemiology, Diffuse Large B-cell lymphoma, Follicular lymphoma, multimorbidity, deprivation, survival analysis

For peer review only

Strengths and limitations of this study

- Data contains a large sample size of high-quality population-based clinical records with a high national coverage of information on all patients diagnosed with Diffuse Large B-cell or Follicular lymphomas in England during 2005 to 2013.
- Population based administrative hospital discharge data was used for the assessment of comorbid conditions, and selection bias was reduced by restricting records of comorbidities to occurring between 6- and 24-months prior to the date of cancer diagnosis.
- Cumulative mortality hazard up to 1 year since cancer diagnosis was modelled using a flexible parametric modelling approach and included restricted cubic splines to account for non-linear effects of continuous variables.
- Regression standardisation was used to control for confounding of patient baseline characteristics; information on lifestyle characteristics was unavailable.
- As there was missing data we performed a sensitivity analysis by comparing complete case analysis-based results to those after multiple imputation: the conclusions were consistent.

For peer review only

Introduction

In England, non-Hodgkin lymphoma (NHL) is the sixth most commonly diagnosed cancer in England with an incidence rate of 23.2 per 100,000 people.[1] Apart from lung cancer, survival estimates of NHL (79.4% survival probability at 1 year) are amongst the lowest of the six most common cancers.[2,3] NHL encompasses a heterogeneous group of malignancies with diverse histological patterns; in addition, the commonest NHL subtypes are diffuse large B-cell lymphoma (DLBCL) and follicular lymphoma (FL),[4] patients of which show a large variation in survival.[5] Cancer survival in England is lower than other European countries,[6] but is similar when restricting to those surviving after 1 year:[7] identifying, and influencing, the factors of short-term mortality could reduce the gap in survival.

Over the past decades, patient survival of FL has steadily improved and stagnated for DLBCL;[3] furthermore, disparities in survival between deprivation levels remains.[8] Public health policies have increased awareness and set targets, such as minimising the length of time from referral to treatment, to reduce the inequalities;[9] however, since their implementation, there has been no evidence that the National Health Service (NHS) Cancer Plan had an impact on the inequalities in cancer survival.[10] The deprivation-gap in survival is still apparent, despite the NHS Cancer Plan and successive policies.[9]

Comorbidities, which refers to the presence of a long-term health condition additional, but unrelated, to the underlying cancer,[11] tends to mask cancer symptoms, delaying cancer diagnosis and decreases survival.[12] Older age groups and those living in more deprived areas experience more comorbidities. With a global aging population, the prevalence of comorbidities is expected to increase.[13] The association between multimorbidity and survival is described for other cancers,[14] but for DLBCL and FL this relationship remains unclear.

We aim to identify the association between multimorbidity and risk of short-term mortality amongst DLBCL and FL patients in England. We hypothesise that multimorbidity and deprivation, independently and combined, contribute to an increased risk of death after DLBCL or FL diagnosis.

For peer review only

Methods

Study Design, participants, data and setting

We used the data from a retrospective population-based cohort study with DLBCL and FL patients diagnosed between 1st January 2005 and 31st December 2013 and followed up to 31st December 2015. DLBCL and FL diagnoses were made according to the International Classification of Diseases for Oncology, 3rd edition, based on codes C82.0-C85.9[15] (**Supplementary Table S1** shows the subtype categorisation). Patients entered the study on the date of their diagnosis and were followed up until death or censored at 1 year, whichever occurred first.

Data was obtained from population-based cancer registries within the English National Cancer Registry and Analysis Service (CAS)[16] and linked to patient's electronic health records from Hospital Episode Statistics (HES). CAS contains patient and tumour variables including relevant dates (birth, diagnosis, and vital status), sex, age at diagnosis, deprivation, cancer site and morphology. We used population based administrative hospital discharge data for the assessment of comorbid conditions; we analysed HES data (containing comorbid conditions records) according to the International Classification of Diseases, 10th revision (**Supplementary Table S2**), for the period 2003 to 2015. HES contains clinical, administrative, and demographic information about individual patients. To avoid selection bias including cancer related comorbidities, we restricted retrospective records of comorbidities to occurring between 6- and 24-months prior to the date of DLBCL and FL diagnosis.[17]

Patient and public involvement

No patient or public involvement.

For peer review only - http://bmjopen.bmj.com/site/about/guidelines.xhtml

Outcome, exposure and other variables

The outcome of this study was the time since diagnosis up to death observed within the first year after diagnosis of DLBLC and FL patients (patients alive were censored at survival time defined with the date of last known vital status), the main exposures were multimorbidity status and deprivation. Due to data availability and clinical reasoning, we include as confounders age, sex, and ethnicity. Due to the positivity assumption, and the chances of having a multimorbidity, we included patients aged above 45 years at diagnosis (**Figure 1**). The positivity assumption states that there is a nonzero probability of receiving any level of comorbidity status for every combination of values of the independent variables among the patients in the population.[18]

Comorbidity status was classified according to the Royal College of Surgeons (RCS) Charlson score (an adaptation of the Charlson comorbidity index[19]) that includes 12 categories for comorbidities, excludes a category (peptic ulcer disease) and groups diseases together (e.g. diabetes mellitus codes with or without complications were grouped into a single category). The score was categorised into those with none, one comorbidity (whatever the type), or two or more comorbidities (defined as *multimorbidity*). The score does not weight the comorbidities assuming that any comorbidity has the same impact on short-term mortality.[20]

Area-level deprivation was categorised into one of five quintiles (5th is most deprived). Deprivation was used as a proxy of individual level socioeconomic status. We used the Index of Multiple Deprivation[21] (IMD), which is an area-level deprivation score based on the Lower Super Output Area[22] (*LSOA*) residence of the patient at the time of cancer diagnosis. LSOA is a geographical location with a median of 1500 inhabitants.

Ethnicity due to data sparsity amongst ethnic minorities was recorded as either white or other. Route to diagnosis (CAS dataset), although not considered in our analysis because it was on the causal pathway, is included in the imputation models for missing data.

Statistical Analysis

We described the characteristics of DLBLC and FL patients using counts and proportions, and calculated odds ratios of having a lymphoma type along with Wald test p-values (**Supplementary Table S3**). We assessed the

For peer review only

unadjusted association between having multimorbidity and patients' characteristics, using chi-square tests. Then, we compute the number of deaths, person-time at risk, and unadjusted rates of deaths per 100 person-years and rate ratios with 95% confidence intervals (CI) by patients' characteristics by DLBCL and FL subtypes.

The follow-up time for those who died is from the date of diagnosis until death, for those alive it was until administrative censoring at 12 months (no lost to follow-up before 12 months was observed). To describe the multimorbidity and deprivation short-term mortality risk amongst DLBCL and FL patients, we computed the one year cumulative hazard obtained using the non-parametric Nelson-Aalen estimator.[23] Then, we computed adjusted short-term mortality risk by patient characteristics using a flexible parametric modelling approach to model the non-linear change in mortality risk over one year. We included restricted-cubic spline to model the baseline hazard,[24] with three knots located at the 25th, 50th, and 75th percentiles of the log event times. To define the model, let t be the time since DLBCL or FL diagnosis until death or censoring. We define the log cumulative motality hazard as

$$\ln\left[H(t \mid \boldsymbol{x}_i, A_i, Dep_i)\right] = \gamma_0 + \gamma_1 z_{1i} + \gamma_2 z_{2i} + \gamma_3 A_{1i} + \gamma_4 A_{2i} + \sum_{k=2}^{5} \beta_{0k} \cdot Dep_{ik} + \sum_{m=1}^{M} \boldsymbol{\beta}_m x_{im}$$

where z_i are the indicators for the three knots of the baseline restricted cubic splines. The model specification included the restricted cubic splines for the continuous variable age are given by A_i where the knots are placed at 1 and 6 months, deprivation, and the vector x_{im} of categorical covariates (i.e., multimorbidity, ethnicity and sex). Restricted cubic splines were included to minimise residual confounding and to account for the non-linear association between age and the cumulative hazard. From the model, we derived the cumulative incidence of death at 1 year by comorbidity status (i.e. none, one, or multimorbidity) standardized to the empirical distribution of age, sex, ethnicity and deprivation.[25,26]

We used the same modelling approach to evaluate the linearly combined effect of multimorbidity and derivation on short-term mortality, assuming the effect of multimorbidity is constant across levels of deprivation.[27] We assume that both effects (multimorbidity and deprivation) are independently associated with the mortality rate and that their effect is constant and the rate is increasing linearly.

For peer review only - http://bmjopen.bmj.com/site/about/guidelines.xhtml

In sensitivity analysis, we evaluated the robustness of our results to the missing ethnicity records by utilising multiple imputation using chained equations (the fully conditional specification multiple imputation approach), under a missing at random assumption. We included route of cancer diagnosis and Ann Arbor[28] cancer stage as partially observed auxiliary variables because these variables were predictive of the probability of missing values (established via exploratory analysis of the missing data indicators) and are predictive of the underlying values themselves (established from clinical and epidemiological reasoning).[29] We generated 20 imputed datasets. The imputation model for the partially observed variable (ethnicity) was defined as a logistic regression model including all explanatory variables in the substantive model, the vital status and Nelson-Aalen estimate of the cumulative hazard, and the auxiliary variables (route to, and stage at, diagnosis). We then fit the substantive model to each of the 20 imputed datasets and from these estimates we predicted the standardised survival (using *stpm2* package), derived the cumulative hazard (-log[Survival probability]) for each imputed dataset and combined the survival probability estimates using Rubin's rules.[30]

Analyses were performed in Stata version 16 (StataCorp, College Station, Texas, U.S.); the *stpm2* package was used to estimate flexible parametric survival models, and the *standsurv* command to compute standardized mortality risks. The *mi impute* command was used for multiple imputation and the *mi estimate* command to combine estimates.

Results

Overall, 41,422 patients in England, aged from 45 to 99 years, were diagnosed with either DLBCL or FL between 2005 and 2013 in England. Of 14,043 patients with FL, amongst those who died within 1-year, the proportion of patients with multimorbidity compared to no comorbidity was 3.2 times higher (22.0% vs. 6.9%, respectively) (**Table 1**); of 27,379 patients diagnosed with DLBCL, this comparison was 1.6 times higher (49.7% vs. 31.5%, respectively). For both DLBCL and FL subtypes, the proportion of multimorbidity was higher amongst those living in more deprived areas. In DLBCL only, there was a higher proportion of having any comorbidity amongst males compared to females ($p<0.001$); for FL only, this proportion was slightly higher amongst females. As expected, multimorbidity was more prevalent amongst older age groups.

For peer review only - http://bmjopen.bmj.com/site/about/guidelines.xhtml

For peer review only

For peer review only

Table 1: Vital status, age, sex, deprivation level and ethnicity according to the comorbidity status amongst n = 41,422 patients with non-Hodgkin lymphoma in England between 2005 and 2013 (27,379 DLBCL cases and 14,043 FL cases).

	No comorbidity N (%)	Comorbidity N(%)	Multimorbidity N(%)	p-value *
**Diffuse Large B-cell lymphoma** (n = 27,379)				
**Vital status at 1 year**				
Alive	16,621 (68.5)	872 (56.7)	791 (50.3)	<0.001
Dead	7,648 (31.5)	666 (43.3)	781 (49.7)	
**Sex**				
Male	12,904 (53.2)	794 (51.6)	954 (60.7)	<0.001
Female	11,365 (46.8)	744 (48.4)	618 (39.3)	
**Age at diagnosis (y)**				
45-54	2,685 (11.1)	80 (5.2)	96 (6.1)	<0.001
55-64	5,154 (21.2)	257 (16.7)	200 (12.7)	
65-74	7,337 (30.2)	432 (28.1)	439 (27.9)	
75+	9,093 (37.5)	769 (50.0)	837 (53.2)	
**Deprivation**				
Least deprived	5,348 (22.0)	291 (18.9)	256 (16.3)	<0.001
2	5,586 (23.0)	318 (20.7)	334 (21.3)	
3	5,115 (21.1)	324 (21.1)	317 (20.2)	
4	4,665 (19.2)	337 (21.9)	339 (21.6)	
Most deprived	3,555 (14.7)	268 (17.4)	326 (20.7)	
**Ethnicity**				
White	17,831 (95.5)	1,204 (96.6)	1,169 (92.6)	<0.001
Other	848 (4.5)	43 (3.5)	93 (7.4)	
**Follicular lymphoma** (n = 14,043)				
**Vital status at 1 year**				
Alive	12,003 (93.1)	546 (87.6)	407 (78.0)	<0.001
Dead	895 (6.9)	77 (12.4)	115 (22.0)	
**Sex**				
Male	5,980 (46.4)	275 (44.1)	257 (49.2)	0.227
Female	6,918 (53.6)	348 (55.9)	265 (50.8)	
**Age at diagnosis (y)**				
45-54	2,246 (17.4)	42 (6.7)	33 (6.3)	<0.001
55-64	3,769 (29.2)	140 (22.5)	82 (15.7)	
65-74	3,952 (30.6)	199 (31.9)	159 (30.5)	
75+	2,931 (22.7)	242 (38.8)	248 (47.5)	
**Deprivation**				
Least deprived	3,091 (24.0)	113 (18.1)	80 (15.3)	<0.001
2	3,025 (23.5)	122 (19.6)	81 (15.5)	
3	2,759 (21.4)	136 (21.8)	118 (22.6)	
4	2,356 (18.3)	123 (19.7)	130 (24.9)	
Most deprived	1,667 (12.9)	129 (20.7)	113 (21.7)	
**Ethnicity**				
White	9,226 (95.7)	498 (96.3)	386 (92.8)	0.012
Other	412 (4.3)	19 (3.7)	30 (7.2)	

Missing values: Ethnicity n(%): DLBCL = 6,191 (22.6%), FL = 3,472 (24.7%)
* Chi-squared test of association between the baseline characteristic and comorbidity status

For peer review only

For peer review only - http://bmjopen.bmj.com/site/about/guidelines.xhtml

Tables 2a and 2b show the person-time at risk and unadjusted mortality rate of death for DLBLC and FL at one year after diagnosis. Amongst FL and DLBCL patients, 1087 (7.7%) and 9,095 (33.2%) died before 1 year, respectively (**Table** 2a and 2b**)**. Amongst FL, those with one comorbidity or multimorbidity had 1.9 (95% CI: 1.46 – 2.33, p<0.001) or 3.5 (95% CI: 2.90 – 4.27, p<0.001) times the mortality rate, respectively, compared to those with no comorbidity (**Table** 2a). Amongst DLBCL, those with one comorbidity or multimorbidity had 1.5 (95% CI: 1.41 – 1.66, p<0.001) or 1.9 (95% CI: 1.78 – 2.06, p<0.001) times the mortality rate, compared to no comorbidity (**Table** 2b).

The unadjusted mortality rate of death increased with each increase in deprivation level: those living in the most deprived areas had 1.5 (95% CI: 1.22 – 1.82, p<0.001) and 1.3 (95% CI: 1.22 – 1.40, p<0.001) times the mortality rate compared to those living in the least deprived areas, for FL and DLBCL, respectively.

For peer review only

For peer review only - http://bmjopen.bmj.com/site/about/guidelines.xhtml

Table 2A: One-year unadjusted mortality rates and rate ratios by sex, age, deprivation, ethnicity, and comorbidity status amongst patients with **Follicular lymphoma** in England between 2005-2015 (n = 14,043; 1,087 deaths at one-year).

	Deaths / person years	Mortality rate* (95% CI)	Mortality RR	95% CI	p-value
**One-year mortality (n = 1,087)**					
**Sex**					
Male	526/6218.12	8.5 (7.77 – 9.21)	Ref		
Female	561/7225.93	7.8 (7.15 – 8.43)	0.92	(0.82 – 1.03)	0.157
**Age at diagnosis (y)** †					
10-year increase	-	-	2.20	(2.08 – 2.34)	<0.001
**Age at diagnosis (y)**					
45-54	46/2300.66	2.0 (1.50 – 2.67)	Ref		
55-64	129/3926.24	3.3 (2.77 – 3.90)	1.64	(1.17 – 2.30)	0.004
65-74	274/4157.52	6.6 (5.86 – 7.42)	3.30	(2.41 – 4.50)	<0.001
75+	638/3059.62	20.9 (19.30 – 22.54)	10.43	(7.73 – 14.07)	<0.001
**Deprivation**					
Least deprived	211/3175.55	6.7 (5.81 – 7.60)	Ref		
2	227/3102.18	7.3 (6.43 – 8.33)	1.10	(0.91 – 1.33)	0.313
3	230/2884.20	8.0 (7.01 – 9.08)	1.20	(1.00 – 1.45)	0.055
4	240/2471.30	9.7 (8.56 – 11.02)	1.46	(1.23 – 1.76)	<0.001
Most deprived	179/1810.81	9.9 (8.54 – 11.45)	1.49	(1.22 – 1.82)	<0.001
**Ethnicity**					
White	742/9712.99	7.6 (7.11 – 8.21)	Ref		
Other	21/450.96	4.7 (3.04 – 7.14)	0.61	(0.40 – 0.94)	0.024
**Comorbidity status**					
None	895/12412.44	7.5 (7.03 – 7.97)	Ref		
One	77/577.89	13.3 (10.66 – 16.66)	1.85	(1.46 – 2.33)	<0.001
Multimorbidity	115/453.71	25.4 (21.11 – 30.43)	3.52	(2.90 – 4.27)	<0.001

* per 100 person-years
† continuous form of age (for each 10-year increase in age)
CI: Confidence interval, **RR:** Rate Ratio, **Missing values:** ethnicity n(%) = 3,472 (24.7%)

For peer review only

Table 2B: One-year unadjusted mortality rates by sex, age, deprivation, ethnicity, and comorbidity status amongst patients with **DLBCL** in England between 2005-2015 (n = 27,379; 9,095 deaths at One-year).

	Deaths/person years	Mortality rate* (95% CI)	Mortality RR	95% CI	p-value
**One-year mortality (n = 9,095)**					
**Sex**					
Male	4867/11318.43	43.0 (41.81 – 44.23)	Ref	Ref	Ref
Female	4228/9784.59	43.2 (41.93 – 44.53)	1.01	(0.96 – 1.05)	0.817
**Age at diagnosis (y)** †					
10-year increase	-	-	1.50	(1.47 – 1.53)	<0.001
**Age at diagnosis (y)**					
45-54	430/2615.81	16.4 (14.96 – 18.07)	Ref	Ref	Ref
55-64	1074/4937.54	21.8 (20.49 – 23.09)	1.32	(1.18 – 1.48)	<0.001
65-74	2365/6604.47	35.8 (34.39 – 37.28)	2.18	(1.97 – 2.41)	<0.001
75+	5226/6945.19	75.2 (73.23 – 77.31)	4.58	(4.15 – 5.05)	<0.001
**Deprivation**					
Least deprived	1765/4684.43	37.7 (35.96 – 38.48)	Ref		
2	1955/4898.09	39.9 (38.18 – 41.72)	1.06	(0.99 – 1.13)	0.079
3	1948/4424.61	44.0 (42.11 – 46.03)	1.17	(1.10 – 1.25)	<0.001
4	1908/4017.48	47.5 (45.41 – 49.67)	1.26	(1.18 – 1.35)	<0.001
Most deprived	1519/3078.40	49.3 (46.92 – 51.89)	1.31	(1.22 – 1.40)	<0.001
**Ethnicity**					
White	6351/15900.73	39.9 (38.97 – 40.94)	Ref		
Other	270/808.61	33.4 (29.64 – 37.62)	0.84	(0.74 – 0.94)	0.004
**Comorbidity status**					
None	7648/19007.45	40.2 (39.35 – 41.15)	Ref		
One	666/1081.20	61.6 (57.09 – 66.46)	1.53	(1.41 – 1.66)	<0.001
Multimorbidity	781/1014.36	77.0 (71.78 – 82.59)	1.91	(1.78 – 2.06)	<0.001

* per 100 person-years
† continuous form of age (for each 10-year increase in age)
CI: Confidence interval, **RR**: Rate Ratio, **Missing values**: ethnicity n(%) = 6,191 (22.6%)

For peer review only

Table 3 shows, before and after multiple imputation, the mortality hazard amongst DLBCL and FL patients at 1 year adjusted for comorbidity status, sex, age, deprivation and ethnicity. After multiple imputation, patients with multimorbidity had 2.2 (CI 1.78 – 2.64, p<0.001) and 1.4 (CI 1.34 – 1.55, p<0.001) times the mortality hazard compared to those without a comorbidity, for FL and DLBCL, respectively. Patients in more deprived areas had 1.5 (CI 1.23 – 1.84, p<0.001) and 1.3 (CI 1.21 – 1.40, p<0.001) times the mortality hazard compared to those living in the least deprived areas, for FL and DLBCL, respectively. There was evidence of a linear trend in mortality hazard by deprivation level for FL (p<0.001) and DLBCL (p<0.001). The direction and magnitude of the hazard ratios after multiple imputation were similar to complete case analysis.

For peer review only

For peer review only - http://bmjopen.bmj.com/site/about/guidelines.xhtml

Table 3: Adjusted hazard ratios of death (before and after multiple imputation) for all patient characteristics amongst patients with (**A**) Follicular or (**B**) DLBCL in England between 2005-2015.

	Complete Case			After multiple imputation		
	**HR***	**95% CI**	**p-value**	**HR***	**95% CI**	**p-value**
**(A) Follicular**						
**Sex**						
Male	Ref	Ref	-	Ref	Ref	-
Female	1.08	0.85 – 1.38	0.540	0.84	0.74 – 0.94	<0.001
**Comorbidity status**						
None	Ref	Ref	-	Ref	Ref	-
One	1.52	1.15 – 2.03	<0.001	1.28	1.01 – 1.61	<0.041
Multimorbidity	2.36	1.85 – 3.02	<0.001	2.17	1.78 – 2.64	<0.001
**Deprivation**†						
Least	Ref	Ref	-	Ref	Ref	-
2	1.03	0.70 – 1.53	0.873	1.09	0.90 – 1.31	0.378
3	1.47	1.00 – 2.16	0.051	1.15	0.95 – 1.39	0.143
4	1.30	0.87 – 1.94	0.200	1.44	1.19 – 1.73	<0.001
Most	1.63	1.11 – 2.41	0.013	1.50	1.23 – 1.84	<0.001
**Ethnicity**						
White	Ref	Ref	-	Ref	Ref	-
Other	0.49	0.27 – 0.88	0.017	0.64	0.40 – 1.01	0.053
**(B) Diffuse large B-cell**						
**Sex**						
Male	Ref	Ref	-	Ref	Ref	-
Female	0.91	0.84 – 0.99	0.170	0.90	0.86 – 0.93	<0.001
**Comorbidity status**						
None	Ref	Ref	-	Ref	Ref	-
One	1.29	1.18 – 1.42		1.24	1.15 – 1.35	<0.001
Multimorbidity	1.61	1.47 – 1.76	<0.001	1.44	1.34 – 1.55	<0.001
**Deprivation**‡						
Least	Ref	Ref	-	Ref	Ref	-
2	1.17	1.03 – 1.33	0.013	1.05	0.98 – 1.12	0.155
3	1.15	1.01 – 1.30	0.029	1.13	1.06 – 1.20	<0.001
4	1.25	1.11 – 1.42	<0.001	1.23	1.15 – 1.31	<0.001
Most	1.32	1.16 – 1.51	<0.001	1.30	1.21 – 1.40	<0.001
**Ethnicity**						
White	Ref	Ref	-	Ref	Ref	-
Other	0.93	0.77 – 1.13	0.463	1.03	0.91 – 1.16	0.678

CI: Confidence interval, **HR**: Hazard Ratio
Missing values: **(A)** ethnicity n(%) = 3,472 (24.7%), **(B)** ethnicity n(%) = 6,191 (22.6%)
* Adjusted for sex, comorbidity status, deprivation, ethnicity and the restricted cubic splines of age
† ‡ Likelihood ratio test for the overall effect of deprivation (p<0.001)

For peer review only

For peer review only - http://bmjopen.bmj.com/site/about/guidelines.xhtml

Table 4 shows the linearly combined effect[14] between comorbidity status and deprivation on short-term mortality by DLBCL and FL. Overall, at 1 year since diagnosis, amongst FL (**Table** 4a), patients who were most deprived with multimorbidity have 3.26 (CI 2.48 – 4.28) times higher short-term mortality hazard than patients without comorbidities and least deprived. Amongst DLBCL (**Table** 4b), and for the same comparison, the short-term mortality hazard was 1.88 (CI 1.70 – 2.07) times higher at 1 year.

Table 4: Linearly combined adjusted hazard ratio of comorbidity status with deprivation level on short-term mortality (after multiple imputation) amongst **[A]** FL (deaths at 1 year: n = 1,087) and **[B]** DLBCL (1 year: n = 9,095) for patients in England from 2005-2015.

	**Comorbidity status**		
	**None**<br>**HR* (95% CI)**	**One**<br>**HR* (95% CI)**	**Multimorbidity**<br>**HR* (95% CI)**
**[A] Follicular**			
**Deprivation**			
Least deprived	Ref	1.28 (1.01 – 1.61)	2.17 (1.78 – 2.64)
2	1.09 (0.90 – 1.31)	1.39 (1.03 – 1.88)	2.36 (1.80 – 3.10)
3	1.15 (0.95 – 1.39)	1.47 (1.09 – 1.98)	2.50 (1.91 – 3.26)
4	1.44 (1.19 – 1.73)	1.84 (1.36 – 2.47)	3.12 (2.40 – 4.06)
Most deprived	1.50 (1.23 – 1.84)	1.92 (1.42 – 2.59)	3.26 (2.48 – 4.28)
**[B] DLBCL**			
**Deprivation**			
Least deprived	Ref	1.24 (1.15 – 1.35)	1.44 (1.34 – 1.55)
2	1.05 (0.98 – 1.12)	1.30 (1.18 – 1.44)	1.51 (1.37 – 1.67)
3	1.13 (1.06 – 1.20)	1.40 (1.27 – 1.55)	1.63 (1.48 – 1.80)
4	1.23 (1.15 – 1.31)	1.52 (1.38 – 1.69)	1.78 (1.61 – 1.95)
Most deprived	1.30 (1.21 – 1.40)	1.62 (1.46 – 1.80)	1.88 (1.70 – 2.07)

CI: Confidence interval. **HR**: Hazard Ratio.
*Adjusted for sex, ethnicity and the restricted cubic splines of age.

For peer review only

Figure 2 shows the unadjusted (Nelson-Aalen non-parametric estimate) and standardised risks of death up to 1 year since diagnosis for FL and DLBCL by comorbidity status and deprivation. **Supplementary Tables** S4a and S4b show results of complete case and after multiple imputation. Standardised to age, sex, deprivation and ethnicity, the risk of death over the first year was consistently higher amongst those with multimorbidity compared to those with one comorbidity or none. For both FL and DLBCL, the unadjusted analysis showed that patients with multimorbidity had consistently higher cumulative incidence of death compared to those with one comorbidity or none (log rank test $p<0.001$).

For peer review only

For peer review only - http://bmjopen.bmj.com/site/about/guidelines.xhtml

Discussion

We aimed to explore the association between comorbidity status, and deprivation and their combination, on short-term mortality for patients with FL or DLBCL. We found that multimorbidity and deprivation and their combined effect are strong independent predictors of short-term mortality amongst patients with DLBCL and FL in England during 2005-2015.

To our knowledge, this is the first study that investigates the association between multimorbidity, and deprivation, on short-term mortality amongst patients with DLBCL and FL in England. Despite the scarcity of research within England, our findings are consistent with previous evidence from other countries. A Swedish study found that higher comorbidity status was independently associated with a higher risk of mortality amongst patients with diffuse large B-cell lymphoma.[31] Additionally, more deprived, compared to least deprived, patients had a higher risk of DLBCL-related mortality and there was evidence of a significant linear trend across the quintiles of deprivation. A Danish study found that higher comorbidity status was independently associated with shorter survival lengths amongst patients with any type of NHL (Hazard Ratio 1.60, CI 1.45-1.75).[32] However, these studies used a non-cancer specific comorbidity score, which underperforms (in comparison to cancer-specific scores) when using predictive models for short-term outcomes.[20] These studies suggest that the effect of comorbidity mainly occurs prior to, and shortly after, cancer diagnosis. Further studies could assess the effect of these prognostic factors on longer term survival from DLBCL or FL, using deprivation-specific life tables to minimise the inaccuracy of expected mortality when life tables are not also stratified by comorbidity status.[33]

As the association between deprivation and NHL survival is not studied as widely as solid tumours, it was unclear whether there was an association between deprivation and short-term mortality for haematological malignancies. Previous studies have described a deprivation-gap in survival comparing the least- to most-deprived,[8,10,34] but have not assessed the association. Although explored for Hodgkin's lymphoma,[35] to our knowledge, this is the first study to explore the association between deprivation and short-term mortality for NHL in England: our study provides evidence of a strong and independent association.

For peer review only

There are several dynamics that may explain the association observed in this study. Firstly, the presence of a comorbidity is known to affect the timely diagnosis of DLBLC and FL,[36] such that comorbidities presenting with similar symptoms to DLBLC or FL may delay the diagnosis and dissimilar symptoms may hasten the diagnosis. Moreover, the prevalence of comorbidities increases with age, and amongst older patients with DLBCL and FL this prevalence is consistently over 60%,[37,38] which may partly explain the delay in diagnosis amongst older ages. Further research is needed to identify comorbidities that alter the timely diagnosis

Secondly, guidelines of lymphoma management focus on a single-disease standard regimen, but there is little guidance on multi-disease management.[39] A systematic review found the majority of patients with a comorbidity did not receive the standard regimen and were allocated alternative, less-intense treatments.[40] Cancer care could be improved by defining clear guidelines that recommend a comorbidity-specific treatment regimen and provide an accurate definition, and a measure, of the dose-intensity.

Thirdly, differences in access to treatments, or risk of adverse effects, may partly explain the multimorbidity gap in survival from DLBCL and FL; clinicians may abstain from allocating a treatment associated with a higher risk of adverse events because it can exacerbate the complex management of cancer care. Patients without comorbidities, after receiving standard treatment regimens, still experience an increased risk of cardiovascular events.[41] A first-line standard treatment for DLBCL is a combination of chemotherapy and immunotherapies, such as rituximab, and is known to be effective for those of an advanced age. Rituximab is often used in combination with anthracyclines (e.g. doxorubicin), which is associated with an increase in the incidence of adverse events (e.g. cardiotoxicity) commonly in the form of congestive heart failure.[42]

Fourthly, due to the indolent nature of FL, we cannot rule out that the increase in short-term mortality we observed might be due to causes of death other than the cancer itself (e.g., multimorbidity). Moreover, the risk of short-term mortality was more pronounced in FL than DLBCL, which may also indicate that other causes of death (other than the cancer) may play a larger role in FL than DLBCL.

For peer review only - http://bmjopen.bmj.com/site/about/guidelines.xhtml

Lastly, the association between deprivation and short-term mortality, that is not explained by patient characteristics, might be explained by the association between deprivation and use of emergency services or population density,[43,44] or between population density and the use of emergency services.[45] For example, population dense areas may accumulate high demands that current facilities of healthcare services are unable to accommodate. Therefore, emergency services (e.g., emergency diagnostic route), which is associated with a late stage of cancer, may explain the higher mortality hazard observed amongst more deprived patients. Further research could investigate the demand and availability of healthcare services in densely populated areas.

The strengths of this study include the large sample size within a database of high-quality population-based clinical records with a high national coverage. We linked clinical records with the HES database, which encompasses all patients in England with a diagnosis of DLBCL and FL between 2005 and 2013. The objective data sources provide information on patients that is gathered prospectively. Furthermore, the standardised risk provides an interpretation of the risk of death that is averaged over the entire population.

Due to data availability, our study has some limitations. Firstly, we did not include tumour stage, route to diagnosis (e.g., general practitioner referral), or treatment plan; consequently, further research is needed to dissect the effects of comorbidity, stage and treatment on survival. Since tumour stage, route and treatment allocation are considered to be on the causal pathway between comorbidity status and short-term mortality, causal inference mediation analysis is required to estimate the proportion of the effect of comorbidity status on survival that is explained by said mediators.

Secondly, recent research highlights the interest in using individual-level socioeconomic measures for assessing patient health outcomes in addition to area-level measures of deprivation.[46] However, information on individual-level socioeconomic measures were unavailable, so wec used only an area-level measure of socioeconomic status, which encapsulates the multidimensional composition of a patient's deprivation level in addition to the contextual level.[21] Furthermore, there is better concordance between area- and individual-level measures of education when assessing patient health outcomes.[46] The observed deprivation level of a patient in our study is likely to be consistent had they been diagnosed at a different time; this is because deprivation scores have a high

For peer review only - http://bmjopen.bmj.com/site/about/guidelines.xhtml

concordance between updates (i.e., IMD of 2007, 2010, and 2015).[21] Our results are comparable to studies using this area-level measure of deprivation.

Thirdly, Hospital Episode Statistics (HES) data contains information on all patients admitted to a hospital (secondary care) in England. It is possible that some comorbidities were not observed because they were diagnosed, and treated, during primary care (e.g., general practitioner consultations). However, the Royal College of Surgeons' comorbidity index, amongst other indices, are constructed based on the impact of the comorbidity on the risk of mortality; in other words, severe comorbidities that require hospitalisation. Comorbidities of the RCS comorbidity index are those that often require hospitalisation, leading to a record within HES data. Previous research has shown that combining primary care records to secondary care data identifies a greater proportion of comorbidity within the population; however, the inclusion of comorbidities identified from primary care records does not have a large effect on predicted cancer survival beyond results obtained using secondary care data.[47]

Fourthly, relative, or cause-specific, survival methods were not used in this study, which would have accounted for competing risks of death. Reliable information on the cause of death was unavailable for all patients diagnosed with DLBCL or FL in England in this study. Net survival within the relative survival setting could be an option, however, there are additional methodological issues when combining net survival with causal inference methods and multiple imputation. Moreover, net survival relies on sufficiently stratified lifetables (e.g., inclusion of comorbid-specific conditions) to correctly account for the background population mortality, which are not currently available.[33]

Lastly, as complete case analysis may lead to selection bias, we performed multiple imputation under a missing at random assumption. We obtained the same conclusions under a complete case analysis and after multiple imputation. Since the missing at random assumption is untestable,[48] further work could conduct a sensitivity analysis to departures from the missing at random assumption, through techniques for imputing under a missing not at random assumption.[49]

For peer review only

In conclusion, multimorbidity and deprivation, combined and independently, are strong predictors of an increased risk of short-term mortality at 1 year since diagnosis amongst patients with DLBCL or FL in England. Therefore, public health prevention strategies are needed to reduce the short-term mortality gap due to socioeconomic inequalities and comorbidities amongst NHL patients.

For peer review only

For peer review only - http://bmjopen.bmj.com/site/about/guidelines.xhtml

Figure Legends

Figure 1: Overlap plots for the density of predicted probabilities of comorbidity status amongst patients (n=41,422), aged 45-99, in England diagnosed with non-Hodgkin lymphoma during 2005-2013. Propensity score: relates to the predicted probability of having any comorbidity level as measured by a multinomial logistic regression model conditioning on the independent variables (i.e., age at diagnosis, sex, deprivation level, and ethnicity).

Figure 2. Risk of short-term mortality for Follicular lymphoma (n=14,043) and DLBCL (n=27,379) by comorbidity status and deprivation level in England between 2005-2015. (Solid: Aalen-Nelson approach, Dash: standardised to the empirical distribution of age, sex, and ethnicity).

For peer review only

For peer review only - http://bmjopen.bmj.com/site/about/guidelines.xhtml

Additional information

Acknowledgements: We thank Professor Paul Lambert (Leicester University, UK) for his advice on using the *stpm2* Stata software package in combination with multiple imputation. We also thank Adrian Turculet (Data Manager, LSHTM Inequalities in Cancer Outcomes Network), for his support with the data linkage.

Author's contributions: MS, ENN and BR contributed to the conception of the study and designed the study. ENN, BR, CL, and MALF provided advice on statistical methods. MS conducted the analyses of the data and prepared the draft of the manuscript, tables and figures. ENN and BR supervised the study and provided comments on the manuscript draft. ENN, BR, MALF, CL, SBM, ABe and ABo provided comments on the final draft of the manuscript. All authors read and approved the final manuscript.

Ethics approval and consent to participate: We obtained the statutory approvals required for this research from the Confidentiality Advisory Group (CAG) of the Health Research Authority (HRA): PIAG 1–05(c) 2007. Ethical approval was obtained from the Research Ethics Committee (REC) of the Health Research Authority (HRA): 07/MRE01/52. This work uses data provided by patients and collected by the National Health Service as part of their care and support. We used anonymised National Cancer Registry and Hospital Episode Statistics data. No consent to participate was sought from patients.

Availability of data and materials: The data that support the findings of this study are available via application to the Public Health England Office for Data Release, but restrictions apply to the availability of these data.

Funding: This research was funded by Cancer Research UK grant number C7923/A18525. The authors declare no support from any organisations for the submitted work. The design of the study, the analyses and the writing of the manuscript were solely the responsibility of the authors. The findings and conclusions in this manuscript are those of the authors and do not necessarily represent the views of Cancer Research UK.

Competing interests: The authors declare there are no competing interests.

For peer review only

References

1. Office for National Statistics. *Cancer registration statistics, England: 2017*. (2019).

2. Cancer Research UK. Non-Hodgkin lymphoma statistics. (2017). Available at: https://www.cancerresearchuk.org/health-professional/cancer-statistics/statistics-by-cancer-type/non-hodgkin-lymphoma. (Accessed: 24th April 2020)

3. Quaresma, M., Coleman, M. P. & Rachet, B. 40-year trends in an index of survival for all cancers combined and survival adjusted for age and sex for each cancer in England and Wales, 1971–2011: a population-based study. *Lancet* **385**, 1206–1218 (2015).

4. Shankland, K. R., Armitage, J. O. & Hancock, B. W. Non-Hodgkin lymphoma. *Lancet* **380**, 848–857 (2012).

5. Epidemiology & Cancer Statistics Group University of York. Haematological Malignancy Research Network. (2016).

6. Allemani, C. *et al.* Global surveillance of trends in cancer survival 2000–14 (CONCORD-3): analysis of individual records for 37,513,025 patients diagnosed with one of 18 cancers from 322 population-based registries in 71 countries. *Lancet* **391**, 1023–1075 (2018).

7. Thomson, C. S. & Forman, D. Cancer survival in England and the influence of early diagnosis: what can we learn from recent EUROCARE results? *Br. J. Cancer* **101**, S102–S109 (2009).

8. Rachet, B., Mitry, E., Shah, A., Cooper, N. & Coleman, M. P. Survival from non-Hodgkin lymphoma in England and Wales up to 2001. *Br. J. Cancer* **99**, S104–S106 (2008).

9. National Institute for Health and Care Excellence. *Haematological cancers: improving outcomes*. (2016).

10. Exarchakou, A., Rachet, B., Belot, A., Maringe, C. & Coleman, M. P. Impact of national cancer policies on cancer survival trends and socioeconomic inequalities in England, 1996-2013: population based study. *BMJ* **360**, k764–k764 (2018).

11. Porta, M. *A Dictionary of Epidemiology*. (Oxford University Press, 2014).

12. Salika, T., Lyratzopoulos, G., Whitaker, K. L., Waller, J. & Renzi, C. Do comorbidities influence help-seeking for cancer alarm symptoms? A population-based survey in England. *J. Public Health (Bangkok)*. **40**, 340–349 (2017).

For peer review only

13. Fowler, H. *et al.* Comorbidity prevalence among cancer patients: a population-based cohort study of four cancers. *BMC Cancer* **20**, 2 (2020).

14. Luque-Fernandez, M. A. *et al.* Multimorbidity and short-term overall mortality among colorectal cancer patients in Spain: A population-based cohort study. *Eur. J. Cancer* **129**, 4–14 (2020).

15. International Agency for Research on Cancer. International Classification of Diseases for Oncology. (2013). Available at: http://codes.iarc.fr/. (Accessed: 4th October 2019)

16. gov.uk. National Cancer Registry and Analysis Service. (2017). Available at: https://www.gov.uk/guidance/national-cancer-registration-and-analysis-service-ncras. (Accessed: 4th October 2019)

17. Maringe, C., Fowler, H., Rachet, B. & Luque-Fernandez, M. A. Reproducibility, reliability and validity of population-based administrative health data for the assessment of cancer non-related comorbidities. *PLoS One* **12**, e0172814 (2017).

18. Hernán, M. Á. & Robins, J. M. Estimating causal effects from epidemiological data. *J. Epidemiol. Community Health* **60**, 578–586 (2006).

19. Charlson, M. E., Pompei, P., Ales, K. L. & MacKenzie, C. R. A new method of classifying prognostic comorbidity in longitudinal studies: Development and validation. *J. Chronic Dis.* **40**, 373–383 (1987).

20. Armitage, J. N. & van der Meulen, J. H. Identifying co-morbidity in surgical patients using administrative data with the Royal College of Surgeons Charlson Score. *Br J Surg* **97**, 772–781 (2010).

21. gov.uk. Indices of Multiple Deprivation. (2015). Available at: https://www.gov.uk/government/statistics/english-indices-of-deprivation-2015. (Accessed: 4th October 2019)

22. National Health Service: data dictionary. Lower Super Output Area. (2018). Available at: https://www.datadictionary.nhs.uk/data_dictionary. (Accessed: 4th October 2019)

23. Armitage, P., Berry, G. & Matthews, J. *Statistical methods in medical research*. (Blackwell Science, 2002).

24. Royston, P. & Lambert, P. *Flexible Parametric Survival Analysis Using Stata: Beyond the Cox Model.* (2011).

25. Robins, J. M., Hernán, M. Á. & Brumback, B. Marginal Structural Models and Causal Inference in

For peer review only

For peer review only - http://bmjopen.bmj.com/site/about/guidelines.xhtml

Epidemiology. *Epidemiology* **11**, (2000).

26. Cole, S. & Hernan, M. A. Adjusted survival curves with inverse probability weights. *Comput. Methods Programs Biomed.* **75**, 45–49 (2004).

27. Dupont, W. D. *Statistical Modeling for Biomedical Researchers: A Simple Introduction to the Analysis of Complex Data*. (Cambridge University Press, 2009).

28. Armitage, J. O. Staging Non-Hodgkin Lymphoma. *CA. Cancer J. Clin.* **55**, 368–376 (2005).

29. Halpern, M. T. *et al.* Association of insurance status and ethnicity with cancer stage at diagnosis for 12 cancer sites: a retrospective analysis. *Lancet Oncol.* **9**, 222–231 (2008).

30. Little, R. J. & Rubin, D. B. *Statistical Analysis with Missing Data*. (John Wiley & Sons, Inc., 1987).

31. Wästerlid, T. *et al.* Impact of comorbidity on disease characteristics, treatment intent and outcome in diffuse large B-cell lymphoma: a Swedish lymphoma register study. *J. Intern. Med.* **285**, 455–468 (2019).

32. Frederiksen, B. L., Dalton, S. O., Osler, M., Steding-Jessen, M. & de Nully Brown, P. Socioeconomic position, treatment, and survival of non-Hodgkin lymphoma in Denmark--a nationwide study. *Br J Cancer* **106**, 988–995 (2012).

33. Rubio, F. J., Rachet, B., Giorgi, R., Maringe, C. & Belot, A. On models for the estimation of the excess mortality hazard in case of insufficiently stratified life tables. *Biostatistics* **22**, 51–67 (2021).

34. Rachet, B. *et al.* Socioeconomic inequalities in cancer survival in England after the NHS cancer plan. *Br J Cancer* **103**, 446–453 (2010).

35. Rafiq, M. *et al.* Socioeconomic deprivation and regional variation in Hodgkin's lymphoma incidence in the UK: A population-based cohort study of 10 million individuals. *BMJ Open* **9**, (2019).

36. Howell, D. A. *et al.* Time-to-diagnosis and symptoms of myeloma, lymphomas and leukaemias: a report from the Haematological Malignancy Research Network. *BMC Blood Disord.* **13**, 9 (2013).

37. van Spronsen, D. J., Janssen-Heijnen, M. L., Breed, W. P. & Coebergh, J. W. Prevalence of co-morbidity and its relationship to treatment among unselected patients with Hodgkin's disease and non-Hodgkin's lymphoma, 1993-1996. *Ann Hematol* **78**, 315–319 (1999).

38. Janssen-Heijnen, M. L. *et al.* A population-based study of severity of comorbidity among patients with non-Hodgkin's lymphoma: prognostic impact independent of International Prognostic Index. *Br J*

For peer review only

For peer review only - http://bmjopen.bmj.com/site/about/guidelines.xhtml

Haematol **129**, 597–606 (2005).

39. National Institute for Health and Care Excellence. *Non-Hodgkin's lymphoma: diagnosis and management*. (National Institute for Health and Care Excellence, 2016).

40. Terret, C., Albrand, G., Rainfray, M. & Soubeyran, P. Impact of comorbidities on the treatment of non-Hodgkin's lymphoma: a systematic review. *Expert Rev. Hematol.* **8**, 329–341 (2015).

41. Linschoten, M. *et al.* Cardiovascular adverse events in patients with non-Hodgkin lymphoma treated with first-line cyclophosphamide, doxorubicin, vincristine, and prednisone (CHOP) or CHOP with rituximab (R-CHOP): a systematic review and meta-analysis. *Lancet Haematol.* **7**, e295–e308 (2020).

42. McGowan, J. V *et al.* Anthracycline Chemotherapy and Cardiotoxicity. *Cardiovasc. Drugs Ther.* **31**, 63–75 (2017).

43. Carlisle, R., Groom, L. M., Avery, A. J., Boot, D. & Earwicker, S. Relation of Out of Hours Activity by General Practice and Accident and Emergency Services with Deprivation in Nottingham: Longitudinal Survey on JSTOR. *BMJ Br. Med. J.* **316**, 520–523 (1998).

44. Venerandi, A., Quattrone, G. & Capra, L. A scalable method to quantify the relationship between urban form and socio-economic indexes. *EPJ Data Sci.* **7**, 4 (2018).

45. Peacock, P. J. & Peacock, J. L. Emergency call work-load, deprivation and population density: An investigation into ambulance services across England. *J. Public Health (Bangkok).* **28**, 111–115 (2006).

46. Ingleby, F. C. *et al.* Assessment of the concordance between individual-level and area-level measures of socio-economic deprivation in a cancer patient cohort in England and Wales. *BMJ Open* **10**, e041714 (2020).

47. Crooks, C. J., West, J. & Card, T. R. A comparison of the recording of comorbidity in primary and secondary care by using the Charlson Index to predict short-term and long-term survival in a routine linked data cohort. *BMJ Open* **5**, e007974 (2015).

48. Carpenter, J. R. & Kenward, M. G. *Multiple Imputation and Its Application*. (John Wiley & Sons, Ltd, 2013).

49. Gachau, S. *et al.* Handling missing data in modelling quality of clinician-prescribed routine care: Sensitivity analysis of departure from Missing at Random (MAR) assumption. *Stat. Methods Med. Res.*

For peer review only

29, 3076 (2020).

For peer review only

For peer review only - http://bmjopen.bmj.com/site/about/guidelines.xhtml

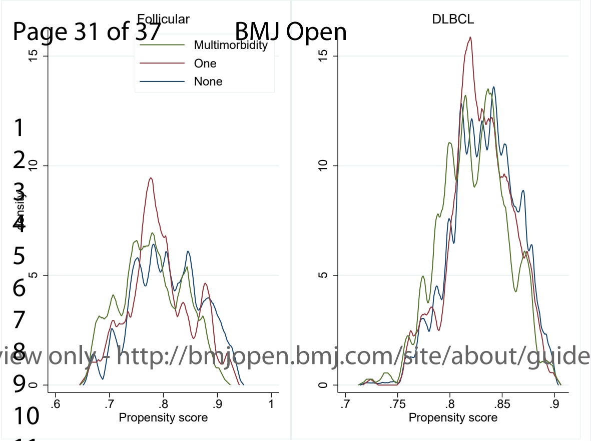

view only - http://bmjopen.bmj.com/site/about/guide

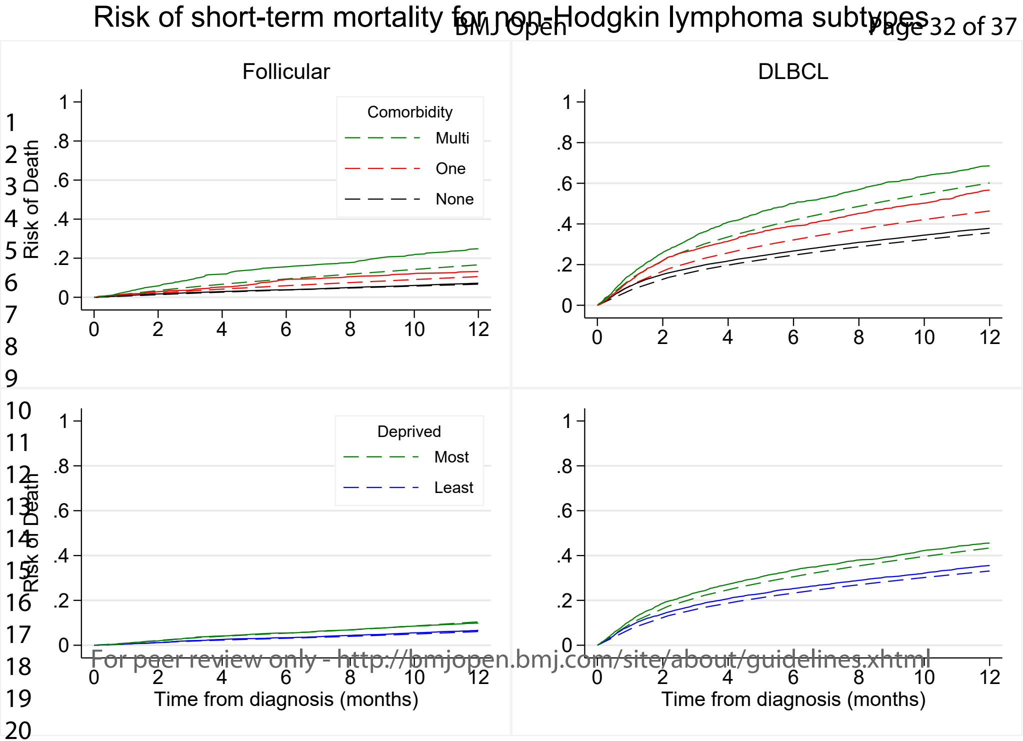

For peer review only - http://bmjopen.bmj.com/site/about/guidelines.xhtml

Supplementary Tables

This page is intentionally blank. Please move to next page.

For peer review only

Supplementary Table S1. Distribution of non-Hodgkin lymphoma subtypes for patients in England diagnosed from 2005-2013, with respective morphology and topography ICD-O-3 codes.

Index	Site group (subtype)	Progression	Topography	Morphology	n	%
1	CLL/SLL*	Indolent	C82.0-C85.9	9670, 9823	3,875	5.08
2	Waldenstrom macroglobulinemia	Indolent	C82.0-C85.9	9761	2,398	3.14
3	Mantle cell	Indolent	C82.0-C85.9	9673	3,458	4.53
4	Diffuse large B-cell	Aggressive	C82.0-C85.9	9680, 9688, 9737-9738	27,379	35.89
5	Burkitt	Aggressive	C82.0-C85.9	9687, 9826	695	0.91
6	Follicular	Indolent	C82.0-C85.9	9690-9691, 9695, 9698	14,043	18.41
7	Mature T-cell	Aggressive	C82.0-C85.9	9702	5,127	6.72
8	Marginal zone B-cell	Indolent	C82.0-C85.9	9689, 9699, 9760, 9764, 9699	4,277	5.61
**Subtotal**					**61,252**	**80.30**
9	Not Otherwise Specified	n/a	C82.0-C85.9	9591, 9675, 9735	9,581	12.56
10	Other***	n/a	C82.0-C85.9	9591, 9675, 9735	5,449	7.14
**Total**					**76,282**	**100.00****

n/a – not applicable; there was no subtype information
* Chronic lymphocytic leukaemia/Small-cell lymphocytic lymphoma
** Percentages may not equate to 100.0% due to rounding
*** The morphology code specifies these patients are diagnosed with NHL. However, the description states ‘other’; these patients are classified similarly to ‘Not Otherwise Specified’.

For peer review only

Supplementary Table S2: Comorbidities and their diagnostic ICD-10 codes

Comorbidity	ICD-10
Myocardial infarction	I21.x, I22.x, I25.2
Congestive heart failure	I11.0, I13.0, I13.2, I25.5, I42.0, I42.5–I42.9, I43.x, I50.x, P29.0
Peripheral vascular disease	I70.x, I71.x, I73.1, I73.8, I73.9, I77.1, I79.0, I79.2, K55.1, K55.8, K55.9, Z95.8, Z95.9
Cerebrovascular disease	G45.x, G46.x, H34.0, I60.x–I69.x
Dementia	F00.x–F03.x, F05.1, G30.x, G31.1
Chronic obstructive pulmonary disease	I27.9, J40.x–J47.x, J60.x–J67.x, J68.4, J70.1, J70.3
Rheumatic disease	M05.x, M06.x, M31.5, M32.x–M34.x, M35.1, M35.3, M36.0
Liver disease	B18.x, K70.0–K70.3, K70.9, K71.3–K71.5, K71.7, K73.x, K74.x, K76.0, K76.2–K76.4, K76.8, K76.9, Z94.4, K71.1, K72.1, K72.9, K76.5, K76.6, K76.7, I85.0, I85.9, I86.4, I98.2, K70.4,
Diabetes without chronic complication	E10.0, E10.1, E10.6, E10.8, E10.9, E11.0, E11.1, E11.6, E11.8, E11.9, E12.0, E12.1, E12.6, E12.8, E12.9, E13.0, E13.1, E13.6, E13.8, E13.9, E14.0, E14.1, E14.6, E14.8, E14.9
Diabetes with chronic complication	E10.7, E11.2–E11.5, E11.7, E12.2–E12.5, E12.7, E13.2–E13.5, E13.7, E14.2–E14.5, E14.7
Hemiplegia or paraplegia	G04.1, G11.4, G80.1, G80.2, G81.x, G82.x, G83.0–G83.4, G83.9
Renal disease	I12.0, I13.1, N03.2–N03.7, N05.2–N05.7, N18.x, N19.x, N25.0, Z49.0–Z49.2, Z94.0, Z99.2
AIDS/HIV	B20.x–B22.x, B24.x

ICD-10: International Classification of Diseases, 10th Revision
Diabetes with/without chronic complication is combined in the RCS Charlson Comorbidity Score

For peer review only

Supplementary Table S3. Sociodemographic characteristics, route of diagnosis, comorbidity status and Ann Arbor cancer stage distribution, of Follicular (n=14,043) and DLBCL (n=27,379) lymphomas in England, 2005-2013.

	Follicular N = 14,043	DLBCL N = 27,379	OR* (95% CI)	p-value
**Age** *(mean; SD)***	66.7 (11.0)	70.8 (11.3)	1.37 (1.35 – 1.40)	<0.001
**Gender**				
Male	6,512 (46.4)	14,652 (53.5)	Ref	Ref
Female	7,531 (53.6)	12,727 (46.5)	0.75 (0.72 - 0.78)	<0.001
**Deprivation**				
Least deprived	3,284 (23.4)	5,895 (21.5)	Ref	Ref
2	3,228 (23.0)	6,238 (22.8)	1.08 (1.01 – 1.14)	0.016
3	3,013 (21.5)	5,756 (21.0)	1.06 (1.00 – 1.13)	0.047
4	2,609 (18.6)	5,341 (19.5)	1.14 (1.07 – 1.22)	<0.001
Most deprived	1,909 (13.6)	4,149 (15.2)	1.21 (1.13 – 1.30)	<0.001
**Comorbidity status**				
Non	12,898 (91.9)	24,269 (88.6)	Ref	Ref
One	623 (4.4)	1,538 (5.6)	1.60 (1.45 – 1.77)	<0.001
Multimorbidity	522 (3.7)	1,572 (5.7)	1.58 (1.43 – 1.75)	<0.001
**Route**				
Elective	11,211 (86.7)	17,280 (66.3)	Ref	Ref
Emergency	1,727 (13.4)	8,799 (33.7)	3.31 (3.12 – 3.50)	<0.001
Missing	*1,105 (7.9)*	*1,300 (4.8)*	-	-
**Stage**				
I	848 (26.6)	1,680 (27.8)	Ref	Ref
II	473 (14.8)	1,169 (19.3)	1.25 (1.09 – 1.43)	0.001
III	822 (25.7)	1,046 (17.3)	0.64 (0.57 – 0.73)	<0.001
IV	1050 (32.9)	2,152 (35.6)	1.03 (0.93 – 1.16)	0.548
Missing	*10,850 (77.3)*	*21,332 (77.9)*	-	-
**Ethnicity**				
White	10,110 (95.6)	20,204 (95.4)	Ref	Ref
Other	461 (4.4)	984 (4.6)	1.07 (0.95 – 1.20)	0.254
Missing	*3,472 (24.7)*	*6,191 (22.6)*	-	-

* Odds ratio from a complete case analysis comparing the odds of DLBCL to Follicular lymphoma
** 10-year increase in age
P-values calculated from Wald tests

For peer review only

Supplementary Table S4a: Risk of short-term mortality amongst patients diagnosed with Follicular lymphomas (n=14,043) by comorbidity status in England between 2005 and 2013

	Complete Case Analysis						After Multiple Imputation					
	Comorbidity status						Comorbidity status					
	None		One		Multimorbidity		None		One		Multimorbidity	
	CH	*95% CI*	*CH*	*95% CI*	*CH*	*95% CI*	*CH*	*95% CI*	*CH*	*95% CI*	*CH*	*95% CI*
**Month**												
1	0.6	0.4 – 0.9	1.0	0.7 - 1.4	1.6	1.0 – 2.3	0.8	0.7 – 1.0	1.3	1.1 – 1.5	2.4	1.6 – 3.2
2	1.4	1.0 – 1.7	2.2	1.6 - 2.7	3.5	2.3 – 4.6	1.7	1.5 – 1.9	2.6	2.2 – 3.0	4.8	3.4 – 6.3
3	2.0	1.6 – 2.4	3.2	2.6 - 3.9	5.2	3.6 – 6.7	2.4	2.2 – 2.7	3.7	3.2 – 4.2	6.8	4.9 – 8.7
4	2.6	2.2 – 3.1	4.2	3.5 - 4.9	6.7	4.8 – 8.6	3.0	2.7 – 3.3	4.6	4.0 – 5.2	8.4	6.1 – 10.8
5	3.2	2.6 – 3.7	5.1	4.3 - 5.9	8.1	5.9 – 10.3	3.6	3.3 – 3.9	5.4	4.8 – 6.1	9.9	7.2 – 12.6
6	3.7	3.1 – 4.3	5.9	5.0 - 6.9	9.4	6.9 – 11.9	4.1	3.8 – 4.4	6.2	5.5 – 7.0	11.3	8.3 – 14.3
7	4.2	3.6 – 4.9	6.7	5.7 - 7.7	10.6	7.8 – 13.4	4.6	4.2 – 5.0	7.0	6.2 – 7.9	12.7	9.3 – 16.0
8	4.7	4.0 – 5.5	7.5	6.4 - 8.6	11.8	8.8 – 14.9	5.1	4.8 – 5.5	7.8	6.9 – 8.8	14.1	10.4 – 17.8
9	5.2	4.5 – 6.0	8.3	7.2 - 9.4	13.1	9.7 – 16.4	5.7	5.3 – 6.1	8.7	7.6 – 9.7	15.5	11.5 – 19.5
10	5.7	5.0 – 6.5	9.1	7.9 - 10.3	14.3	10.6 – 17.9	6.2	5.8 – 6.7	9.5	8.4 – 10.6	17.0	12.6 – 21.3
11	6.2	5.4 – 7.0	9.9	8.6 - 11.1	15.4	11.5 – 19.4	6.8	6.4 – 7.3	10.3	9.2 – 11.5	18.4	13.7 – 23.1
12	6.7	5.9 – 7.5	10.6	9.2 - 12.0	16.6	12.3 – 20.9	7.4	6.9 – 7.8	11.2	9.9 – 12.4	19.8	14.8 – 24.9

CH – cumulative hazard of death (e x 10^2)
95% CI – confidence interval (e x 10^2)

Supplementary Table S4b: Risk of short-term mortality amongst patients diagnosed with DLBCL (n=27,379) by comorbidity status in England between 2005 and 2013

	Complete Case Analysis						After Multiple Imputation					
	Comorbidity status						Comorbidity status					
	None		One		Multimorbidity		None		One		Multimorbidity	
	CH	*95% CI*	*CH*	*95% CI*	*CH*	*95% CI*	*CH*	*95% CI*	*CH*	*95% CI*	*CH*	*95% CI*
**Month**												
1	7.4	6.9 – 7.9	9.7	9.0 – 10.4	13.2	11.5 – 14.9	9.2	8.9 – 9.6	12.0	11.4 – 12.5	16.1	14.5 – 17.7
2	12.9	12.2 – 13.7	17.0	16.0 – 18.0	23.0	20.4 – 25.7	15.3	14.8 – 15.7	19.7	18.8 – 20.5	26.3	23.9 – 28.8
3	16.8	16.0 – 17.6	22.0	20.8 – 23.2	30.0	26.6 – 33.3	19.1	18.6 – 19.7	24.6	23.6 – 25.6	32.8	29.8 – 35.8
4	19.8	18.9 – 20.7	25.9	24.5 – 27.2	35.4	31.5 – 39.3	22.1	21.6 – 22.7	28.4	27.2 – 29.5	37.7	34.3 – 41.1
5	22.4	21.4 – 23.3	29.2	27.7 – 30.7	39.9	35.6 – 44.3	24.8	24.1 – 25.4	31.7	30.4 – 32.9	42.0	38.2 – 45.8
6	24.7	23.7 – 25.7	32.2	30.6 – 33.8	43.9	39.2 – 48.7	27.1	26.5 – 27.8	34.7	33.3 – 36.0	45.9	41.8 – 50.0
7	26.8	25.8 – 27.8	34.9	33.3 – 36.6	47.5	42.4 – 52.6	29.3	28.6 – 30.0	37.5	36.0 – 38.9	49.6	45.2 – 53.9
8	28.8	27.7 – 29.8	37.5	35.8 – 39.2	50.8	45.4 – 56.2	31.4	30.7 – 32.1	40.1	38.5 – 41.6	53.0	48.3 – 57.6
9	30.6	29.5 – 31.7	39.9	38.1 – 41.7	53.8	48.2 – 59.5	33.3	32.6 – 34.0	42.5	40.9 – 44.1	56.2	51.2 – 61.1
10	32.4	31.2 – 33.5	42.2	40.3 – 44.0	56.7	50.8 – 62.6	35.1	34.4 – 35.9	44.8	43.2 – 46.5	59.2	54.0 – 64.4
11	34.0	32.8 – 35.2	44.3	42.4 – 46.3	59.4	53.3 – 65.5	36.9	36.1 – 37.7	47.1	45.3 – 48.8	62.1	56.7 – 67.6
12	35.6	34.4 – 36.9	46.4	44.3 – 48.5	62.0	55.6 – 68.4	38.6	37.7 – 39.4	49.2	47.4 – 51.0	64.9	59.2 – 70.6

CH – cumulative hazard (e x 10^2)
95% CI – confidence interval (e x 10^2)

For peer review only

For peer review only

For peer review only - http://bmjopen.bmj.com/site/about/guidelines.xhtml
